# The Open acidification Tank Controller: An open-source device for the control of pH and temperature in ocean acidification experiments

**DOI:** 10.1016/j.ohx.2023.e00435

**Published:** 2023-05-30

**Authors:** Kirt L Onthank, James Foster, E. Preston Carman Jr, John E. Foster, Monica Culler-Juarez, Eliam Calvo, Wesley Duerksen, Trevor Natiuk, Lucas Saca

**Affiliations:** a Department of Biological Sciences, Walla Walla University, College Place, WA, United States; b Department of Computer Science, Walla Walla University, College Place, WA, United States; c Department of Mathematics, Walla Walla University, College Place, WA, United States

**Keywords:** pH stat device, Arduino, Lab sensor, Ocean acidification

## Abstract

Ocean acidification is the process by which the increase in atmospheric CO_2_ causes a corresponding increase in seawater CO_2_ and results in lowering the seawater pH. While this process is likely to have substantial impacts on marine ecosystems, research into the effect of ocean acidification has been limited by the high costs of quality tools to perform ocean acidification treatments in the lab. The Open Acidification Tank Controller is designed to reduce the cost of ocean acidification research by providing a device that can monitor and control pH and temperature of aquaria as well as or better than commercially available research-grade devices, but for less than $250 USD per aquarium. The device is centered around an Arduino Mega 2560 and is assembled into a 3D printed housing. It monitors pH using a BNC glass pH probe and temperature using a three-wire waterproof PT100 temperature sensor. The Open Acidification Tank Controller also features web-based parameter reporting, and data storage to a micro-SD card. This device can hold aquarium pH and temperature at given setpoints, ramp between two values over a user-defined time period, or produce a sine-wave fluctuation in values.

## Graphical abstract

**Figure:**
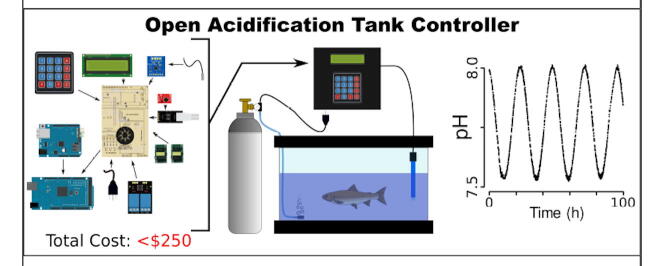

## Specifications table

**Table:** 

Hardware name	The Open Acidification Tank Controller
Subject area	• Biological Sciences • Environmental, Planetary and Agricultural Sciences • Educational Tools and Open-Source Alternatives to Existing Infrastructure
Hardware type	• Measuring physical properties and in-lab sensors • Biological sample handling and preparation • Electrical engineering and computer science
Closest commercial analog	Loligo Systems pH and CO_2_ Control System
Open-Source License	Software: MPL v2.0 Housing: CERN-OHL-W PCB: CERN-OHL-W
Cost of Hardware	<$250
Source File Repository	Software: https://doi.org/10.5281/zenodo.5508206Housing : https://doi.org/10.5281/zenodo.5348212PCB : https://doi.org/10.5281/zenodo.5348218
OSHWA Certification UID	US002053

## Hardware in context

Since the Industrial Revolution carbon dioxide has risen in the atmosphere from 280 ppm to over 420 ppm. Much of this anthropogenic carbon dioxide becomes dissolved in oceans where it reacts with seawater to create carbonic acid and then bicarbonate and carbonate (releasing a proton with each step of that reaction). This process is known as ocean acidification [1], [2]. If CO_2_ emissions are not abated, ocean pH will drop from its pre-industrial level of 8.2 to approximately 7.8 by the end of this century, a 250% increase in acidity. Ocean acidification will undoubtedly have dramatic effects on marine organisms and ecosystems [3], [4]. While ocean acidification research has increased over the last couple decades, there is still far too little research to fully comprehend the dramatic effects of ocean acidification. Much more research is needed.

Unfortunately, a limiting factor to the initiation of most lines of research in ocean acidification is the cost of equipment. At a bare minimum, most ocean acidification experiments need some methods of manipulating seawater carbon dioxide content in an aquarium in which experimental organisms are held. It is recommended that ocean acidification experiments target two values: a CO_2_ partial pressure (pCO_2_) of 750 μatm, the projected average pCO_2_ at sea level in 2100, and the current pCO_2_ of approximately 400 μatm [5]. In seawater of alkalinity of 2300 μmol kg^−1^, salinity of 35 psu, and temperature of 19C (all approximately ocean average), these pCO_2_ targets would result in seawater pH of 7.810 and 8.064, respectively. In these seawater conditions, a deviation of 0.1 pH units from 7.810 to 7.710 corresponds to an increase in pCO_2_ of 218 μatm. Therefore, pH in ocean acidification experiments must be controlled within a few hundredths of the setpoint.

This control can be accomplished in one of several ways. Strong acid can be added to the seawater, but this method only changes seawater alkalinity, without changing the total dissolved inorganic carbon and thus does not appropriately simulate future ocean conditions [6], [7], [8]. Alternatively, carbonate or bicarbonate could be added to the seawater, followed by a strong acid, but this is technically difficult due to the materials used and this method cannot be used directly in the aquarium holding the organisms because of the substantial swings in seawater chemistry during additions [8]. Another approach is to place the aquaria in an environmental chamber with a manipulated carbon dioxide level and let the water absorb CO_2_ from the air [8]. But the method that best simulates future ocean conditions and is relatively easy to accomplish is the continuous or intermittent bubbling of gas in seawater [8]. One of the most common methods is to use a pH-stat system in which the pH of an aquarium is measured with some type of pH probe and pure gaseous CO_2_ is intermittently bubbled either directly into the aquarium or into a header tank to obtain the desired pH. This method tends to be the most cost-effective operationally due to the lower cost of pure carbon dioxide compared to custom mixed gas and the lower amount of gas needed to manipulate the water pH. Yet it is still costly in terms of controlling equipment.

The cost problem is compounded by the issue of pseudoreplication. That is, in order to have statistically significant results, researchers need a fair number of samples for tests that can run for days or weeks. Organisms housed in the same pH-controlled aquarium at the same time, or that even share a common header tank, cannot be considered independent replicates. This means that organisms must be held in controlled aquaria sequentially (greatly extending the time of the experiment), or in parallel (greatly increasing the cost of equipment) [9].

There are a few commercially available systems that allow the control of temperature and pH in aquaria. Loligo Systems produces a system intended for scientific research [10], while a number of devices are produced for the hobbyist aquarium [11]. The Loligo Systems OmniCTRL (formerly CapCTRL) system costs approximately $7,500 USD (7,108 euros) to control a single aquarium, plus about $2100 (1,893 euros) for each additional tank. At the top end of the hobbyist market is the Neptune ApexEL, costing $560 USD, which is often used for ocean acidification experiments [12]. The most inexpensive hobbyist pH controller unit is the American Marine Inc. Pinpoint pH Controller costing $200 [13]. More recently, open-source applications have been published to control aquarium pH, but these devices have invariably been feature-poor, relied on expensive external equipment, or both [14], [15], [16].

The purpose of the Open Acidification Tank Controller is to provide plans for a device that can monitor and control pH and temperature of aquaria as well as or better than commercially available research-grade devices and do so for less than $250 USD.

## Hardware description

The Open Acidification Tank Controller is designed to support ocean acidification research by providing the following functionality:

• Monitor pH and temperature for an aquarium
• Control pH by adding CO2 if the pH is above a configured value
• Control temperature by turning on a heater or chiller (depending on the configuration)
• Provide a user interface for setting configuration values and observing current values
• Record configurations and observations to a micro-SD card for later analysis
• Allow web-based management and reporting

The most expensive part of most ocean acidification tank control systems is the pH probe and meter. A variety of pH probes are available that have a BNC male connector, so researchers can use their preferred probe (costs range from $30 to well over $500). The pH probe connects to the Tank Controller via a BNC female connector on an Electrically Isolated EZO™ Carrier Board (P10; $42) to which is attached an EZO™ pH Circuit (P9; $42) that provides pH readings to the CPU from 0.001 to 14.000 with a resolution of 0.001 and an accuracy of +/- 0.002. This brings the cost of the pH meter assembly to under $90, while other open-source projects have employed high-end external pH meters, such as the Honeywell UDA 2182 Dual Analyzer which costs approximately $1500 [15]. However, this pH meter system performs admirably and was selected from an array of other possible pH meter circuits because, in our testing while developing this system, it could perform high accuracy pH measurements with inexpensive pH probes.

To monitor the temperature, we use a three-wire waterproof PT100 temperature sensor (P4; $13) attached to a (male) 3.5 mm TRS plug (P25; $7). The sensor connects to the Tank Controller with a (female) 3.5 mm jack which leads to a MAX31865 Temperature Sensor Amplifier (P3; $15) that provides resistance data to the CPU from which it calculates a temperature.

The *control* aspect of the system is provided by switching on and off standard 110-volt AC power to attached external devices using the 2-channel relay module. These modules use two Songle SRD-05VDC-SL-C relays, which are rated for 125VAC/10A resistive load (aquarium heaters) and 250VAC/10A inductive load (aquarium chillers). To change the water temperature, the researcher provides a heater or chiller (depending on whether the aquarium is to be kept above or below ambient room temperature) and plugs that device into the Tank Controller. If the temperature set-point of the tank is close to the temperature of the room in which it is contained, both a heater and a chiller may need to be employed with only one connected to the Tank Controller. Typically, in these situations we use a low-power aquarium heater connected to constant power and a higher power aquarium chiller connected to the Tank Controller. To change pH, the researcher provides a 12 V solenoid valve (typically using 2.5 W) attached to a CO_2_ regulator controlled by an AC line plugged into the Tank Controller. (We only support *adding* CO_2_ to the water, not removing it.).

The Tank Controller has a single male 3-prong AC power entry module (P18) that is connected to 110 V AC with a standard power cable (like that used for a desktop computer or monitor) and two 3-prong AC female outlets (P19) used to control the pH and temperature (as described above). Internally the Tank Controller has a 2 channel DC 5 V relay module (P7; $7) to control the high-voltage AC outlets.

A basic user interface is provided on the device with a 4x4 keypad (P6; $1) for input and a 1602 LCD display module (P5; $5) for output. Data storage and external communication is provided by an Ethernet shield with a mini-SD card slot (P2; $19). The Tank Controller is managed by an Arduino Mega 2560 or compatible CPU board (P1; $21) with a real time clock breakout board (P8; $7). Power to the internal components is supplied by external 110 V AC through a pair of buck converter step down modules, one providing 5 V DC (P40; $4) to the relays and one providing 9 V DC (P41; $4) to the Arduino which is then stepped down to 7 V using a switch voltage regulator on the custom PCB to avoid overheating the Arduino’s step-down converter.

While the Ethernet shield attaches directly to the Arduino board, the remaining pieces attach to a custom printed-circuit board (PCB) and the pieces are assembled into a 3D printed housing (see the design files and build instructions below). Software is installed using the Arduino IDE on an external computer (Linux, macOS, or Windows) connected to the device using a USB cable. When connected by Ethernet to a network, the device settings can be viewed and configured using a web browser.

The Open Acidification Tank Controller can hold a pH setpoint, as is common among all comparable devices, but can also ramp pH linearly between two setpoints, which is only available on high-end research-grade systems such as the Loligo Systems OmiCTRL, and can also rhythmically modulate pH in a sine wave–a functionality not currently available in any comparable device to our knowledge. Ocean acidification researchers are increasingly becoming aware that pH variability, and not just a static pH value, may influence organismal responses to environmental pH change [17]. Therefore, devices that can model pH fluctuations will be essential to future ocean acidification research.

## Design files summary

**Table:** 

Design file name	File type	Open-source license	Location of the file
TankController-21.09.1.zip	Software source code	MPL v2.0	Zenodo
Back_Plate.FCStd	FreeCAD CAD file	CERN-OHL-W	Zenodo
Front_Plate.FCStd	FreeCAD CAD file	CERN-OHL-W	Zenodo
back_plate.stl	3D mesh	CERN-OHL-W	Zenodo
front_plate.stl	3D mesh	CERN-OHL-W	Zenodo
skirt.stl	3D mesh	CERN-OHL-W	Zenodo
Half_Shield.kicad_pcb	KiCad PCB CAD file	CERN-OHL-W	Zenodo
Half_Shield.pro	KiCad PCB CAD file	CERN-OHL-W	Zenodo
Half_Shield.sch	KiCad PCB CAD file	CERN-OHL-W	Zenodo

• TankController-21.09.1.zip – Software installed on the device.
• Back_Plate.FCStd – FreeCad file to modify the backplate
• Front_Plate.FCStd – FreeCad file to modify the frontplate
• back_plate.stl – Stereolithography file describing the back (bottom) of the device housing.
• front_plate.stl – Stereolithography file describing the front (top) of the device housing.
• skirt.stl – Stereolithography file describing the skirt (four sides) of the device housing.
• Half_Shield.kicad_pcb – The KiCad file describing the PCB.
• Half_Shield.pro – The KiCad file describing the project.
• Half-Shield.sch – The KiCad file describing the schematics.

## Bill of materials

Prices shown below are per unit, though the selected source might have multiple units. For example, the 4x4 keypad (P6) comes in quantity 5 and items like screws and nuts (P42-P45) come in quantities of 25–100. Prices are effective January 1, 2023.

## Bill of materials

**Table:** 

Designator	Component	Number	$ / unit	Total $	Source of materials	Material type
P1	Elegoo Arduino Mega clone	1	$20.99	$20.99	Amazon	other
P2	KEYESTUDIO Ethernet shield	1	$18.99	$18.99	Amazon	other
P3	Adafruit MAX31865	1	$14.95	$14.95	Adafruit	other
P4	PT100 temp sensor (3-wire)	1	$12.98	$12.98	Amazon	other
P5	1602 LCD	1	$4.50	$4.50	Amazon	other
P6	4x4 keypad	1	$1.80	$1.80	Amazon	other
P7	2 channel relay module	1	$6.79	$6.79	Amazon	other
P8	Adafruit PCF8523 Real Time Clock	1	$6.95	$6.95	Adafruit	other
P9	Atlas Scientific EZO pH circuit	1	$45.99	$45.99	Atlas	other
P10	Atlas Scientific Electrically isolated carrier board	1	$41.99	$41.99	Atlas	other
P11	Screw Terminal Block − 5	1	$1.31	$1.31	Digikey	other
P12	18x2 male pins	1	$1.84	$1.84	Digikey	other
P13	4x1 male pins	2	$0.19	$0.38	Digikey	other
P14	6x1 male pins	1	$0.35	$0.35	Digikey	other
P15	8x1 male pins	1	$0.26	$0.26	Digikey	other
P16	16x1 male pins	2	$0.70	$1.40	Digikey	other
P17	3x1 male pins	6	$0.19	$1.14	Digikey	other
P18	power receptacle	1	$1.76	$1.76	Digikey	other
P19	Power outlet	2	$1.38	$2.76	Digikey	other
P20	16x1 female socket	1	$0.98	$0.98	Digikey	other
P21	8x1 female socket	1	$0.65	$0.65	Digikey	other
P22	4x1 female socket	1	$0.45	$0.45	Digikey	other
P23	3x1 female socket	1	$0.42	$0.42	Digikey	other
P24	5x1 female socket	2	$0.47	$0.94	Digikey	other
P25	3.5 mm TRS jack	1	$7.10	$7.10	Digikey	other
P26	1x1 female socket	3	$0.24	$0.72	Digikey	other
P27	3.5 mm jack receptacle	1	$1.32	$1.32	Digikey	other
P28	Vishay potentiometer	1	$1.67	$1.67	Digikey	other
P29	Push Button	1	$1.39	$1.39	Digikey	other
P30	220-ohm resistor SMD 0805	1	$0.10	$0.10	Digikey	other
P31	0.1 uF capacitor SMD 0805	7	$0.10	$0.60	Digikey	other
P32	10 uF capacitor SMD 0805	1	$0.11	$0.11	Digikey	other
P33	0.1 uF Kemet capacitor SMD 603	1	$0.30	$0.30	Digikey	other
P34	22 uF TDK capacitor SMD 0805	1	$0.59	$0.59	Digikey	other
P35	47 uF TDK capacitor SMD 1206	1	$0.96	$0.96	Digikey	other
P36	4.7 uH Bourns Inductor	1	$0.59	$0.59	Digikey	other
P37	10 kOhm resistor SMD 0805	1	$0.40	$0.40	Digikey	other
P38	TI Switching regulator	1	$0.63	$0.63	Digikey	other
P39	82.5 kOhm resistor SMD 0805	1	$0.36	$0.36	Digikey	other
P40	5 V AC DC buck converter module	1	$2.60	$2.60	Amazon	other
P41	9 V AC DC buck converter module	1	$2.36	$2.36	Amazon	other
P42	M4 × 0.7 Press-fit Nuts	8	$0.27	$2.16	McMaster-Carr	other
P43	M4 × 0.7 12 mm hex drive flat head bolt	8	$0.12	$0.96	McMaster-Carr	other
P44	M3 × 0.05 10 mm socket head screw	4	$0.13	$0.52	McMaster-Carr	other
P45	M3 × 0.05 nut	4	$0.03	$0.12	McMaster-Carr	other
P46	14-gauge wire (white, green, red)					other
P47	22-gauge wire					other
P48	Standard 3-prong power cable	1	$4.99	$4.99	Amazon	other
P49	DuPont jumper wires	1	$6.99	$6.99	Amazon	other
P50	Jumper	5	$0.10	$0.50	Digikey	other

In addition to the materials listed above, the housing will need to be 3D printed and the printed circuit board (PCB) manufactured. Using a standard fused deposition modeling (FDM) printer and 20% in-fill, printing all 3 housing components requires approximately 250 g of polylactic acid (PLA) filament before considering any supports that may be needed. Cost of PCB manufacture can vary widely with the company used but can be as little as 5 USD.

## Build instructions

### Tools needed

• Soldering iron & solder
• Pliers
• Wire cutter
• Wire stripper
• Desoldering pump (solder sucker)
• Breadboard
• Voltmeter
• Metric 2.5 Allen wrench
• 5.5 mm socket wrench
• Helping hands tool

### Housing and PCB manufacture

#### Construct the Housing

Using a fused deposition modeling (FDM) 3D printer and polylactic acid (PLA) filament, print the files listed above in section 3, “back_plate.stl”, “front_plate.stl”, and “skirt.stl”, to make the housing for the Tank Controller.

#### PCB Manufacture

**Fig. 1:**
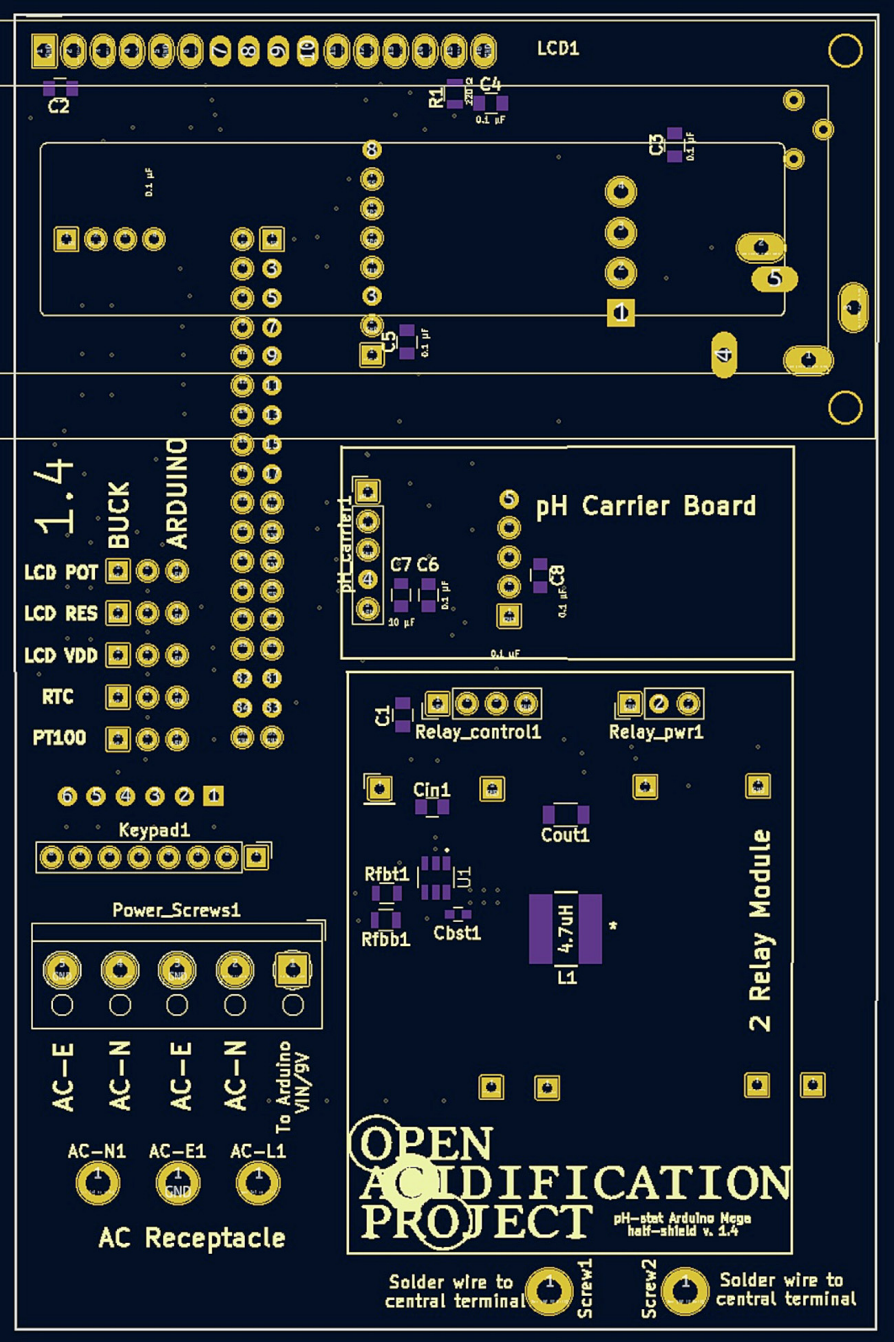
The front of the PCB with the Open Acidification Project logo.

**Fig. 2:**
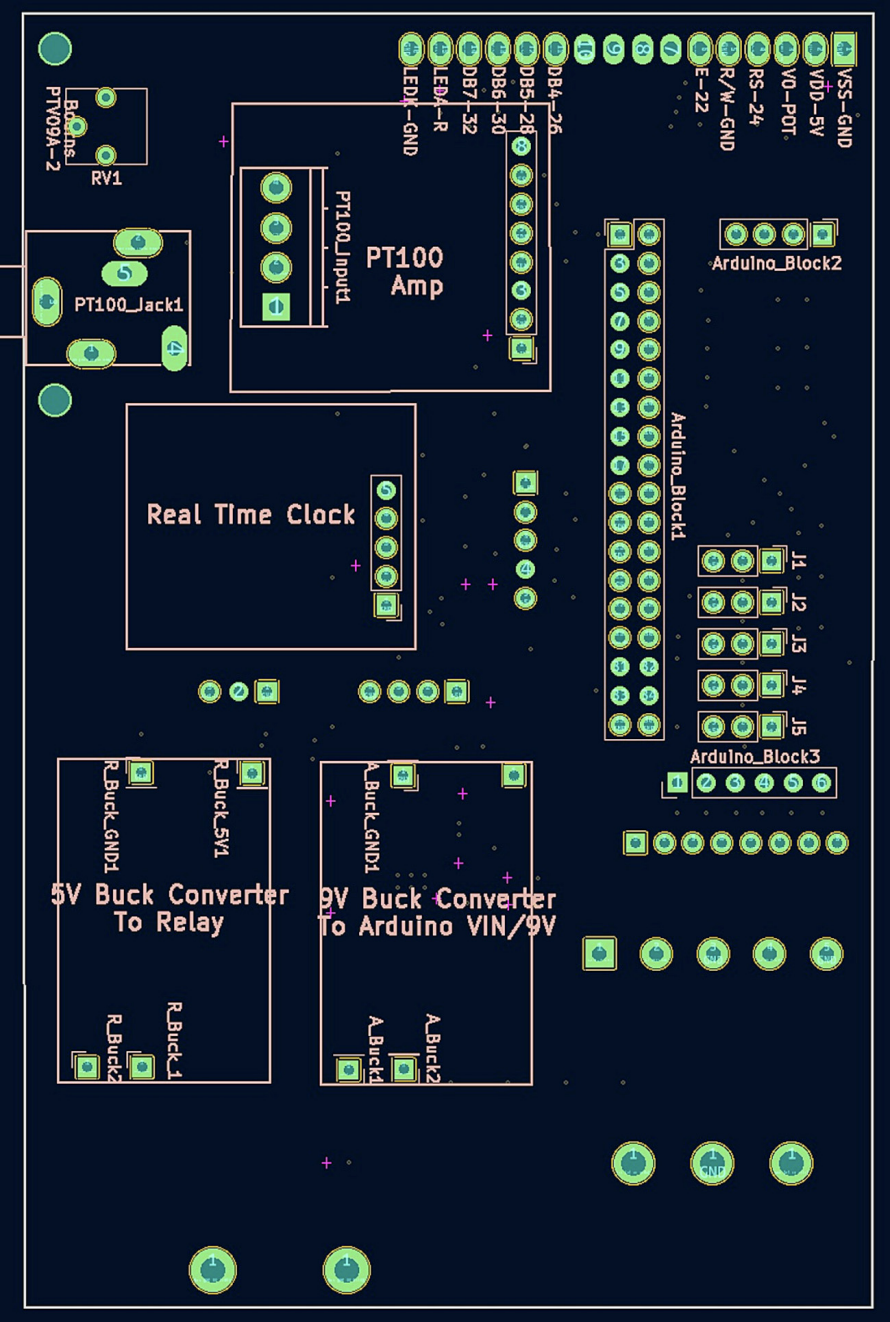
The back of the PCB.

o Using the source KiCad files referenced above in section 3 “Design files,” generate Gerber (.gbr) and Drill (.drl) files.
o Submit these files to a PCB manufacturing fabricator.
o You should receive back a PCB of about 3″ x 4.5″ (74.5236 mm × 114.2492 mm) (Fig. 1, Fig. 2).

### Apply surface-mount components to PCB

The following components are attached directly to the PCB using a surface-mount process:

• 220-ohm resistor SMD 0805 (P30)
• Seven 0.1 uF capacitor SMD 0805 (P31)
• 10 uF capacitor SMD 0805 (P32)
• uF Kemet capacitor SMD 603 (P33)
• 22 uF TDK capacitor SMD 0805 (P34)
• 47 uF TDK capacitor SMD 1206 (P35)
• 4.7 uH Bourns Inductor (P36)
• 10 kOhm resistor SMD 0805 (P37)
• TI Switching regulator (P38)
• 82.5 kOhm resistor SMD 0805 (P39)

The locations and part numbers are as follows:

**Table:** 

Location (see Fig. 1)	Part Number (see Bill of Materials)
C1	0.1 uF capacitor SMD 0805 (P33)
C2	0.1 uF capacitor SMD 0805
C3	0.1 uF capacitor SMD 0805
C4	0.1 uF capacitor SMD 0805
C5	0.1 uF capacitor SMD 0805
C6	0.1 uF capacitor SMD 0805
C7	10 uF capacitor SMD 0805 (P32)
C8	0.1 uF capacitor SMD 0805
R1	220-ohm resistor SMD 0805 (P30)
Cin1	22 uF TDK capacitor SMD 0805 (P34)
Cout1	47 uF TDK capacitor SMD 1206 (P35)
Rfbt1	82.5 kOhm resistor SMD 0805 (P39)
Rfbb1	10 kOhm resistor SMD 0805 (P37)
U1	TI Switching regulator (P38)
Cbst1	0.1 uF Kemet capacitor SMD 603 (P33)
L1	4.7 uH Bourns Inductor (P36)

**Fig. 3:**
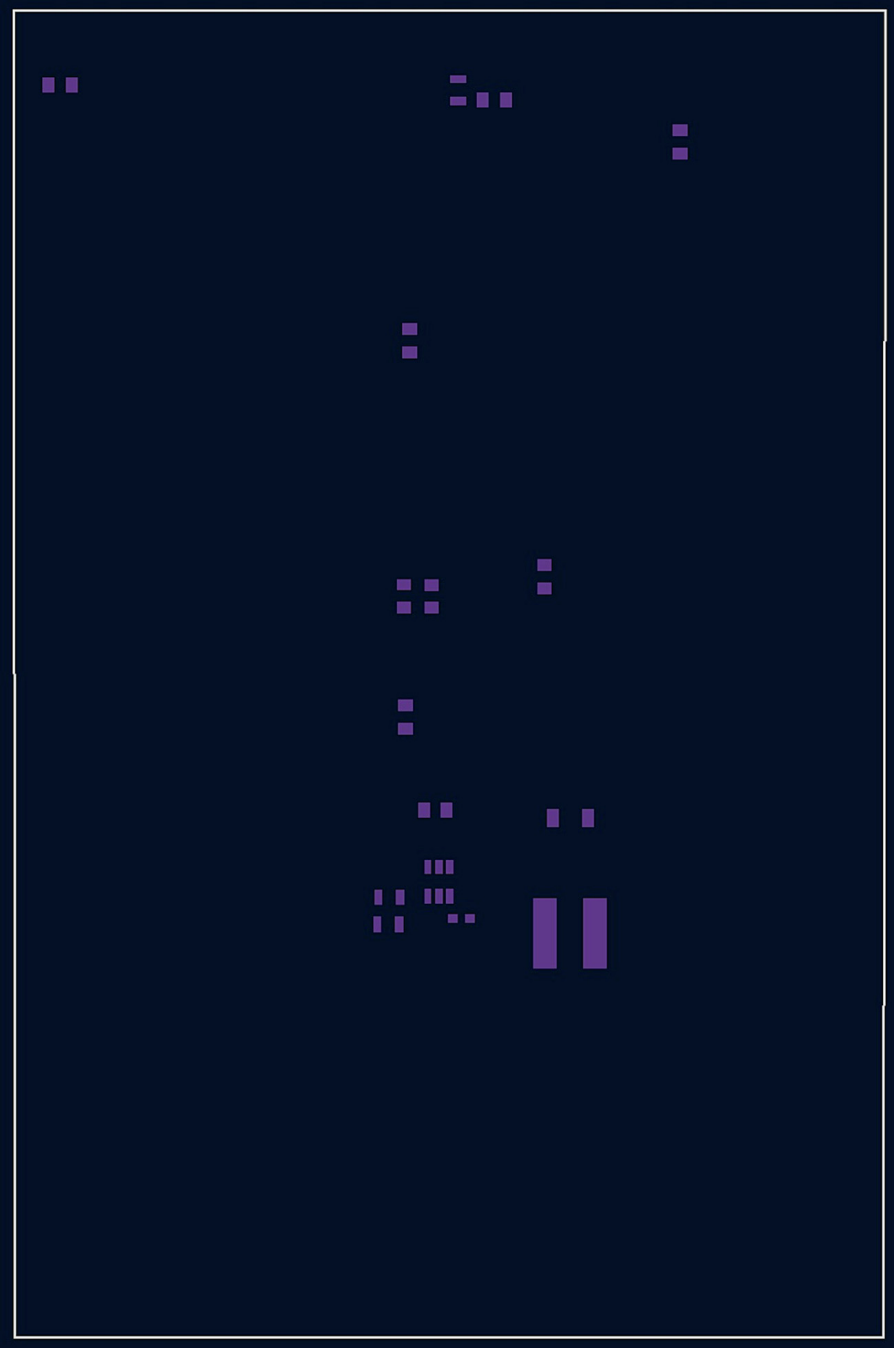
Surface-mount template.

There are (at least) three approaches to this task. First, just as the PCB is custom-made by an outside firm, there are many firms that will do the surface-mount assembly. This is certainly the easiest and most reliable approach. Another option (if you have access to the equipment) is to use a pick-and-place machine and a reflow oven. Finally, you can manually place solder paste on the PCB using a stencil (Fig. 3), add the components one at a time (very carefully with tweezers), and then heat the PCB in an oven until the solder melts (but not so long as to distort the surface). Most of our devices were assembled at our School of Engineering using their equipment, but a few have been assembled with manual placement and heated in a toaster oven.

### Prepare components

Several of the components that will be attached to the PCB are shipped with through-holes to which the user can attach wires or header pins facing up or down. In some cases, the header pins are included (but not connected), in some cases the component does not include header pins so we need to provide them, and in some cases the pins are pre-installed. If the pins are pre-installed, then they might be in the desired position or opposite how we wish to have them positioned. In this step of the construction, we prepare the components to be attached to the PCB.

#### Adafruit PCF8523 Real Time Clock (RTC) Assembled Breakout Board (P8)

**Fig. 4:**
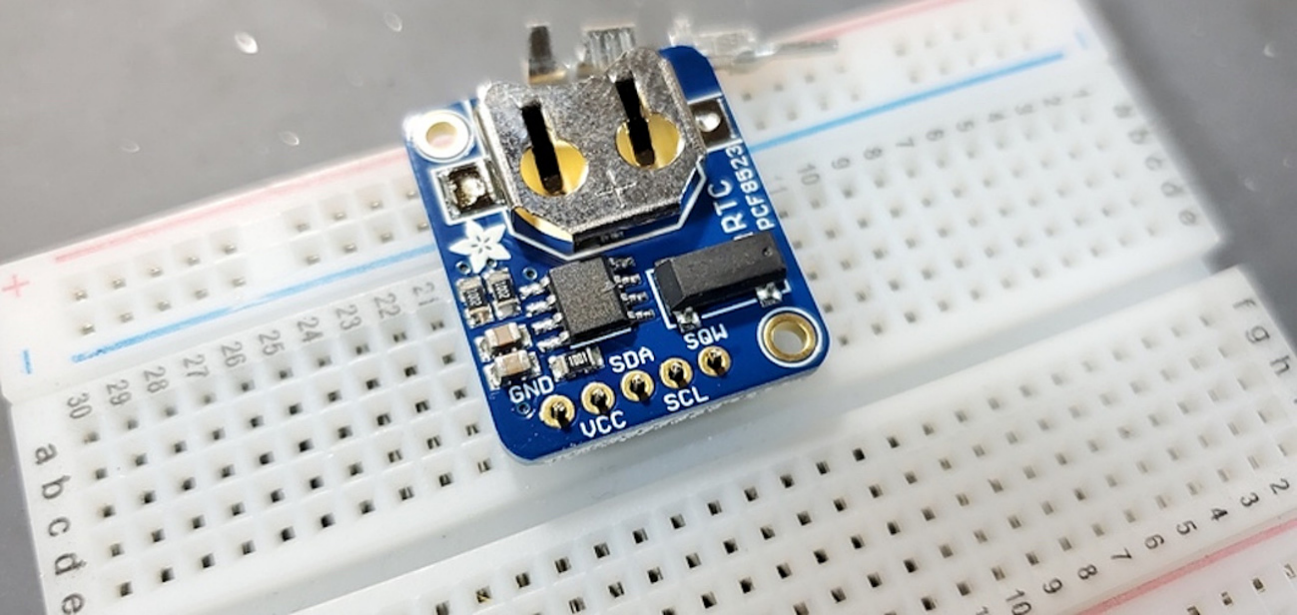
PCF8323 Real Time Clock Breakout Board ready for header pins to be soldered.

This component ships with a 5x1 set of header pins. We need the pins facing down (away from the battery), so they will be soldered from the front of the board. A good way to do this is to put the male pins into a breadboard, then place the breakout board on top of the pins (perhaps with some padding to have the breakout board at a right angle from the pins; Fig. 4). You can then solder the header pins and remove the breakout board from the breadboard.

#### Adafruit PT100 RTD Temperature Sensor Amplifier - MAX31865

**Fig. 5:**
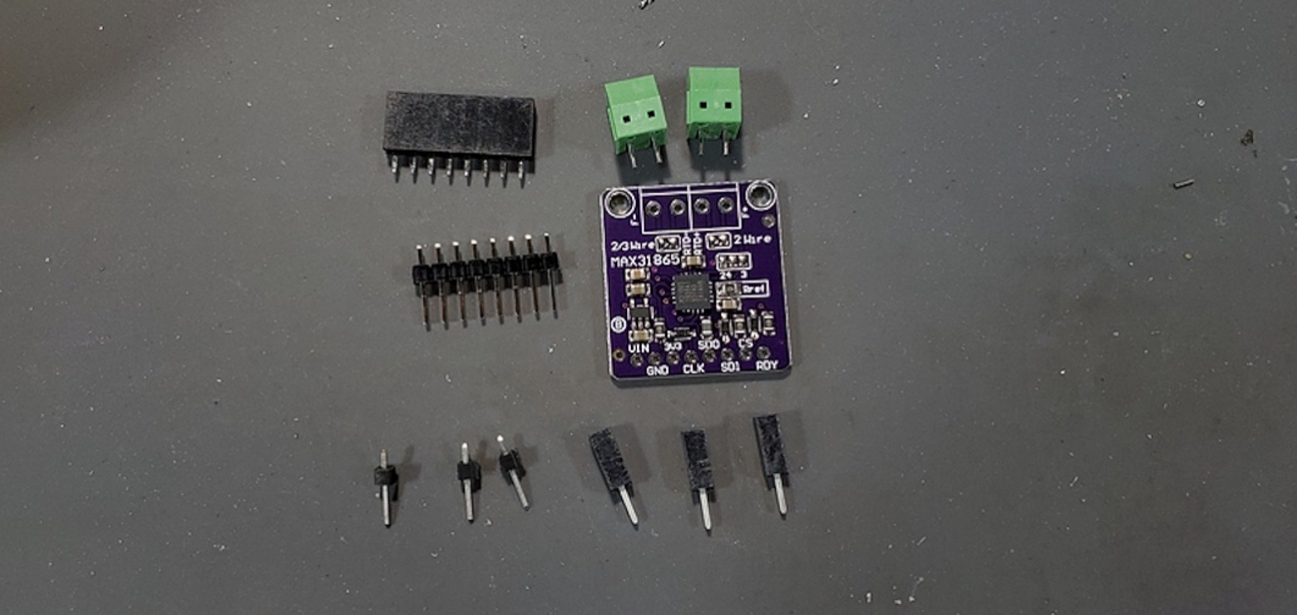
PT100 amplifier breakout board (P3).

This amplifier breakout board ships with two 2-pin terminal blocks (that we don’t use) and an 8x1 male pin header (Fig. 5). The front of the breakout board contains the surface mounted components, and we want the pins to be on the back (so soldered from the front).

**Fig. 6:**
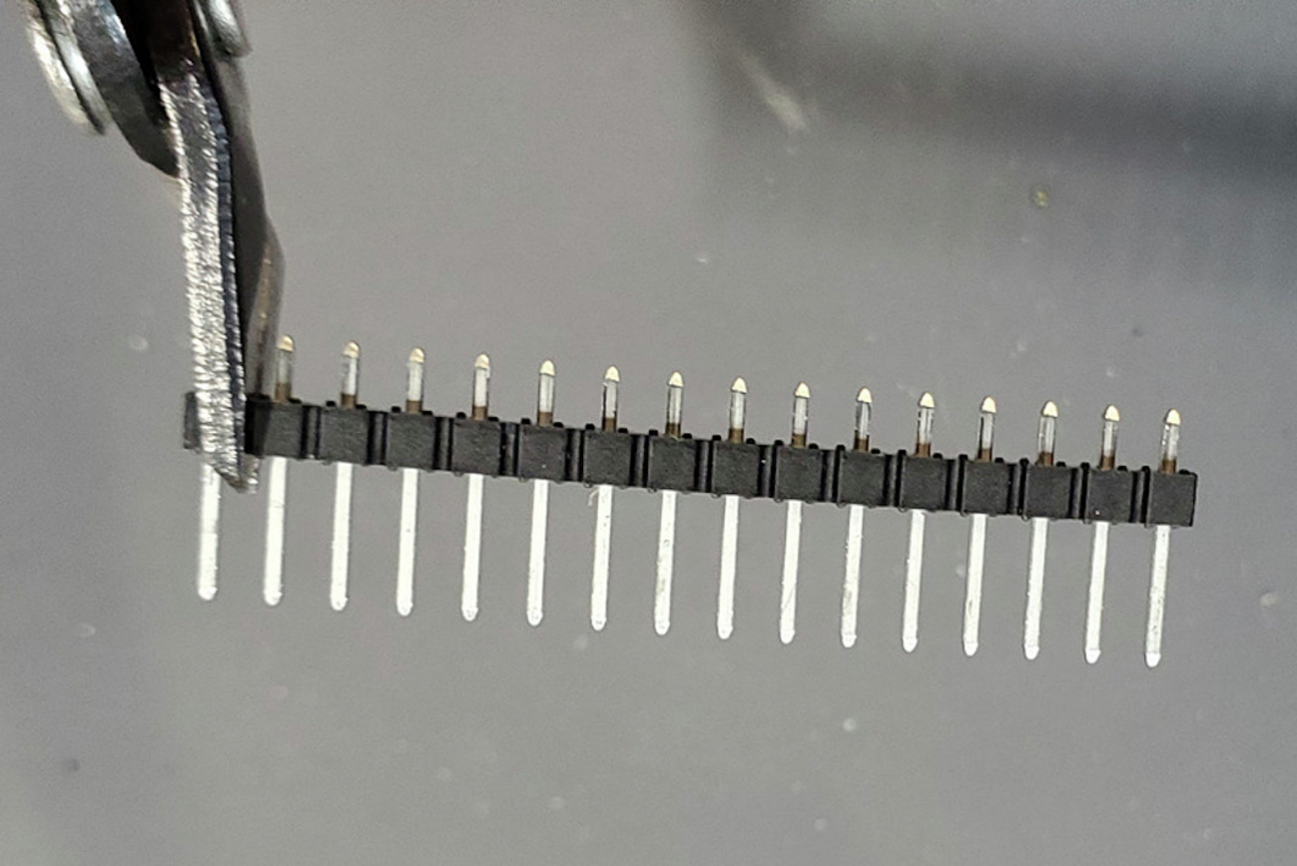
Clip three individual pins from a 16x1 header (P16).

In addition to the 8x1 male pins provided with the device, we will take three male pins off a 16x1 pin header (Fig. 6).

**Fig. 7:**
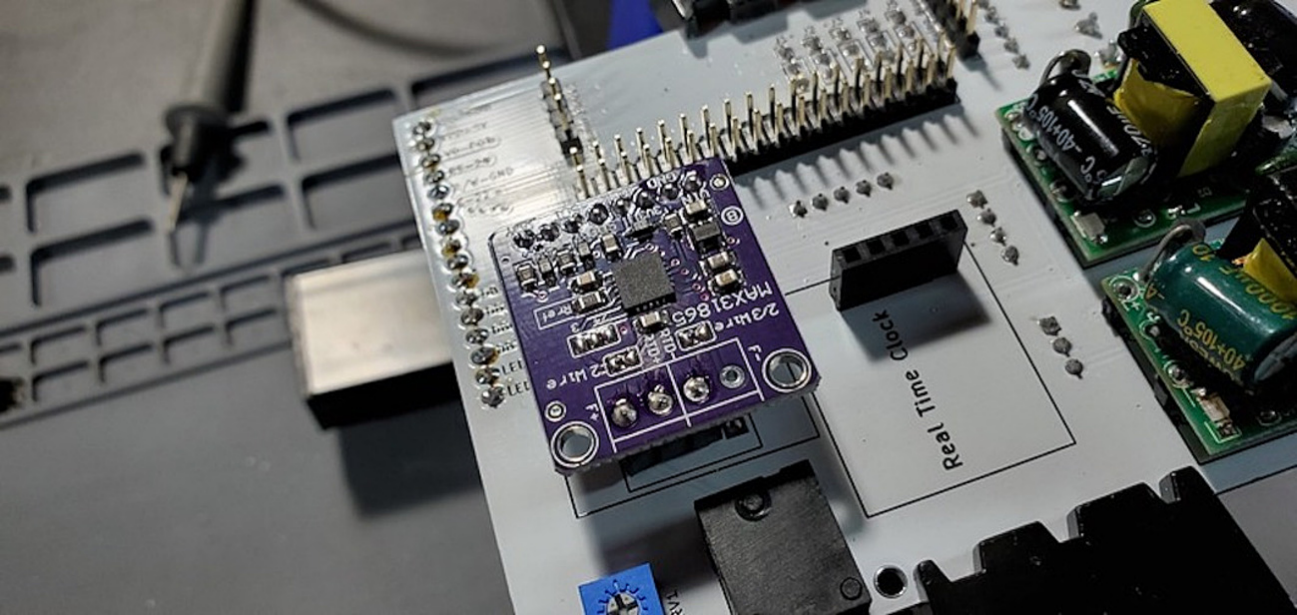
PT100 breakout board with pins soldered in place.

The 8x1 male pin header is soldered to the breakout board at the eight through-holes at the bottom of the breakout board. The other three pins are attached to the right-most three of the four through-holes at the top of the board (toward the “F+” lettering, leaving one blank through-hole next to the “F-” lettering). As with the RTC assembly, the 8x1 pins can be soldered using a breadboard for positioning, but the three other pins do not have the same spacing so must be handled separately. One option is to use the custom PCB itself as a template and drop the pins through the holes labeled “PT100_Input1” (choose the three round through-holes, not the square one). Or, you can mate the three 1x1 female sockets with the male pins and put the pin portion of the female sockets into the custom PCB (Fig. 7).

**Fig. 8:**
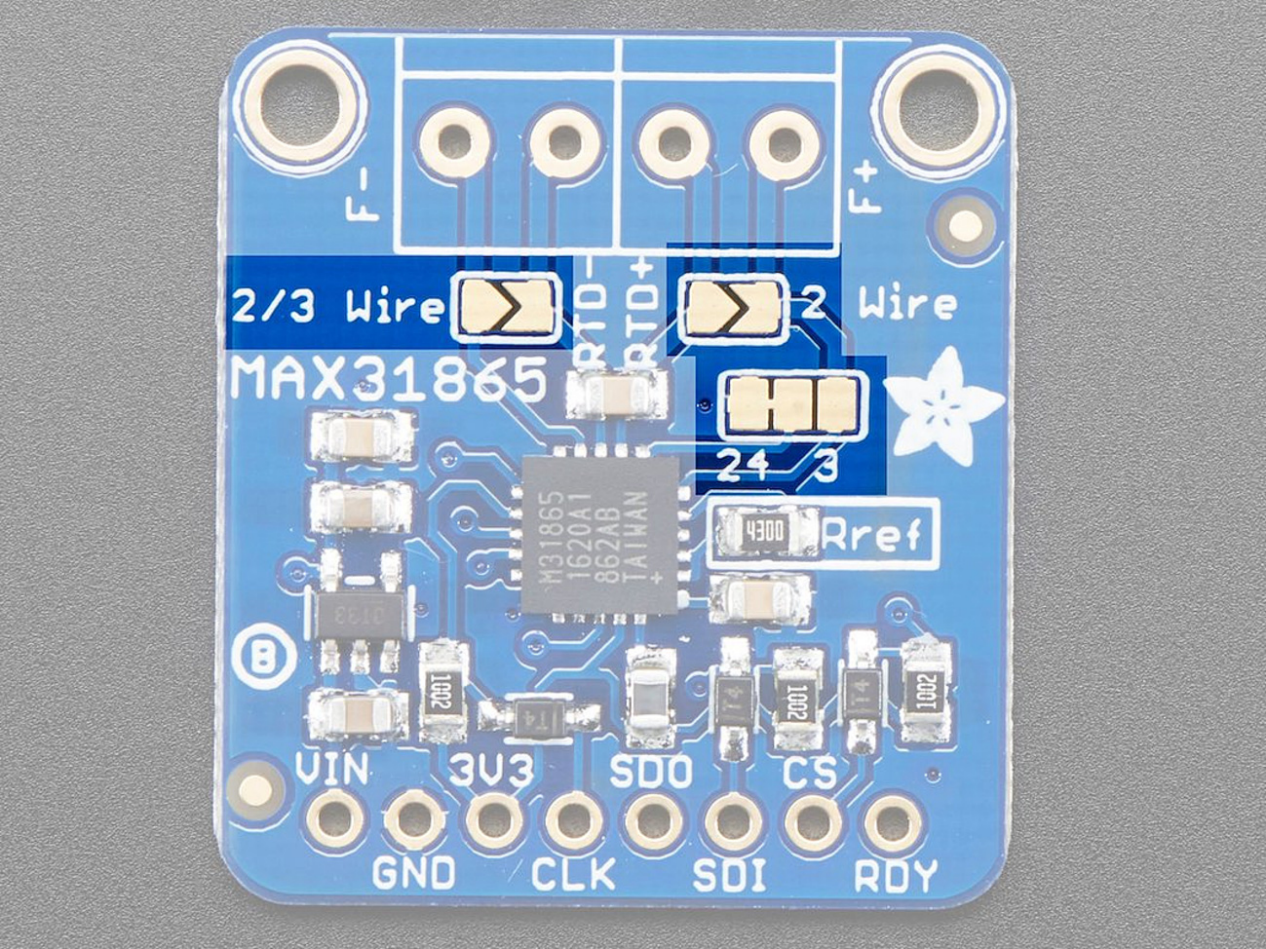
PT100 with configuration jumpers highlighted (image from https://learn.adafruit.com/adafruit-max31865-rtd-pt100-amplifier/pinouts).

**Fig. 9:**
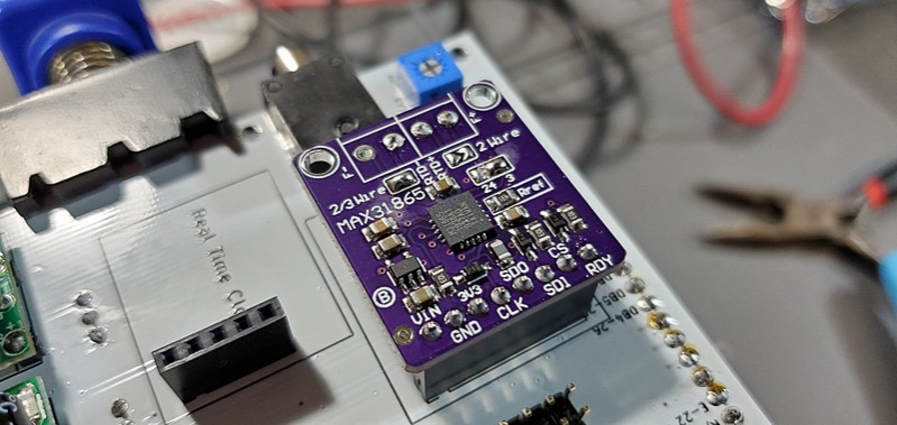
PT 100 with pins attached and jumper configuration.

As shipped, the breakout board is configured for a 4-wire connection. As noted above, we are using a 3-wire temperature probe, so we need to change the configure of the board to work with a 3-wire probe (Fig. 8, Fig. 9). Start by identifying the jumper locations:

o Solder together the pads that are labeled “2/3 Wire”.
o Cut the wire connecting the left two pads of the 2-way jumper labeled “24 3” above the “Rref” label so that the “24” pad is no longer connected to the center pad (the pointed tip of wire cutters work well for this).
o Finally, solder together the right two pads so that the “3″ pad is connected to the center pad.

#### 1602 LCD Display Module (P5)

**Fig. 10:**
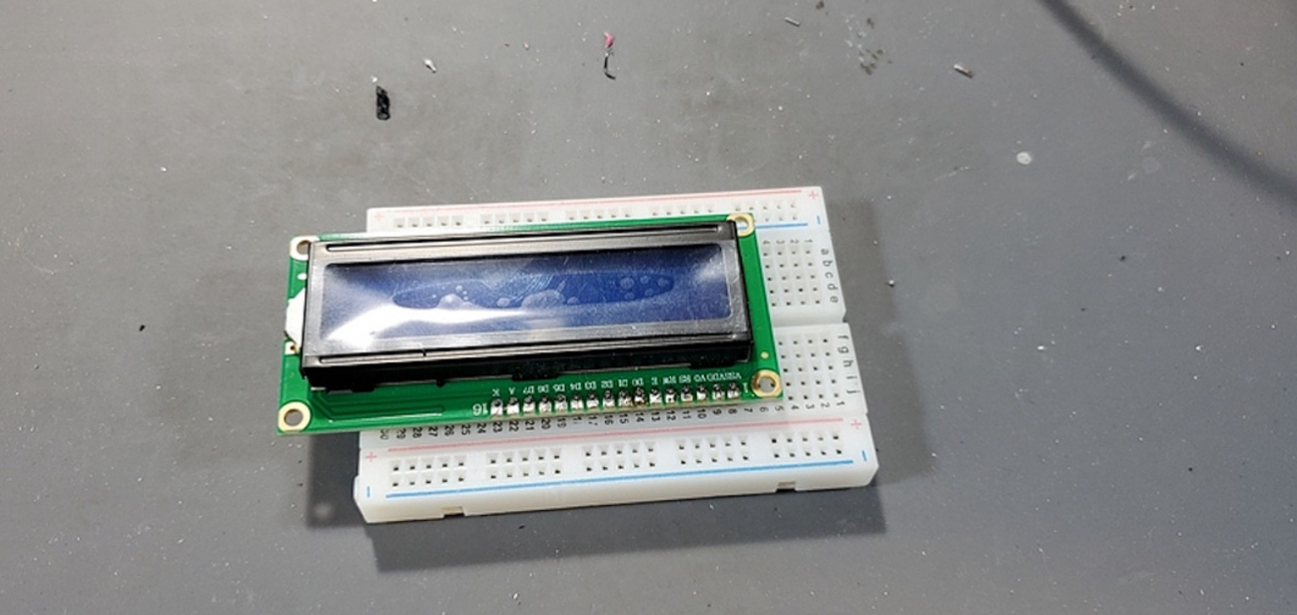
1602 LCD display module with pins.

This two-line 16-column display does not include any pins, so we use a 16x1 male pin set (P16) and attach them to the back of the board (soldering from the front; Fig. 10).

#### Buck Converter Modules (P40 and P41)

**Fig. 11:**
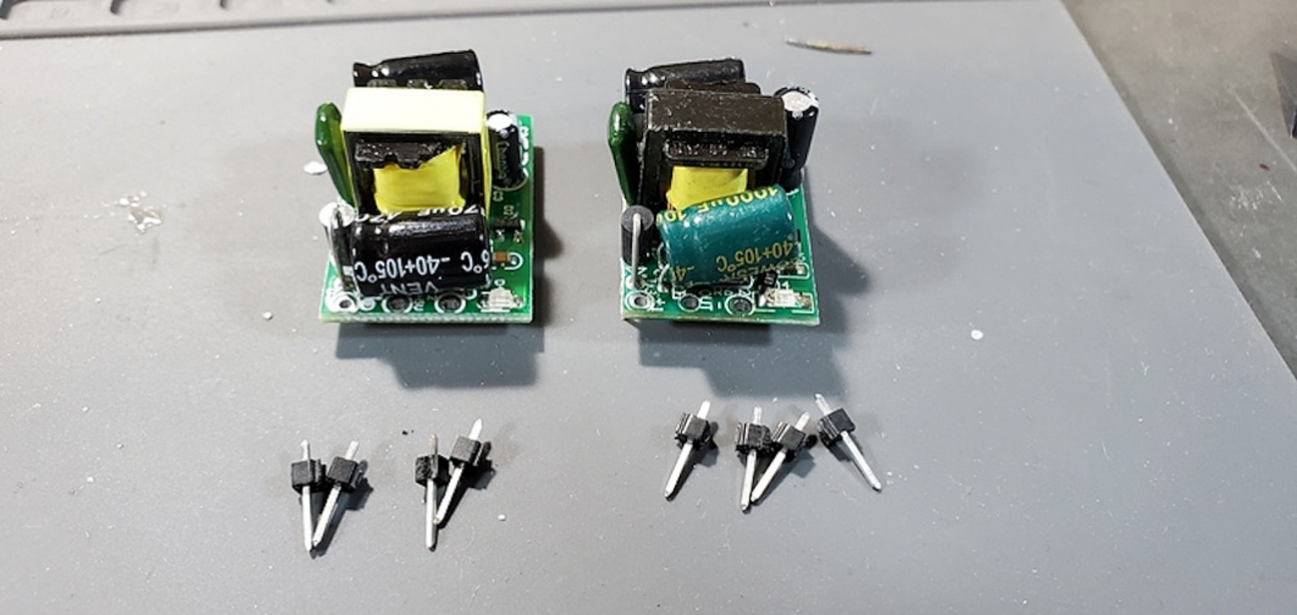
Buck converts with pins.

**Fig. 12:**
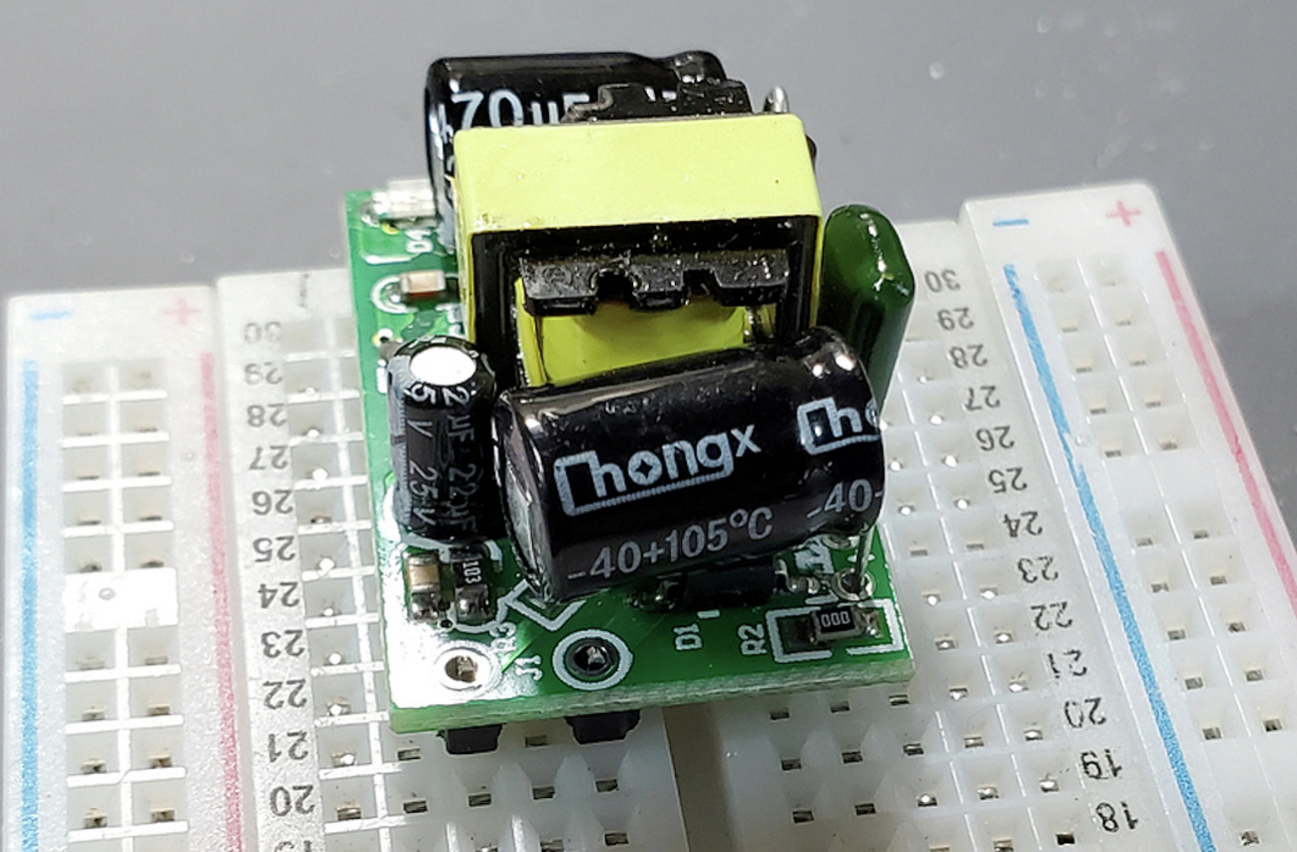
Buck converter AC pins.

**Fig. 13:**
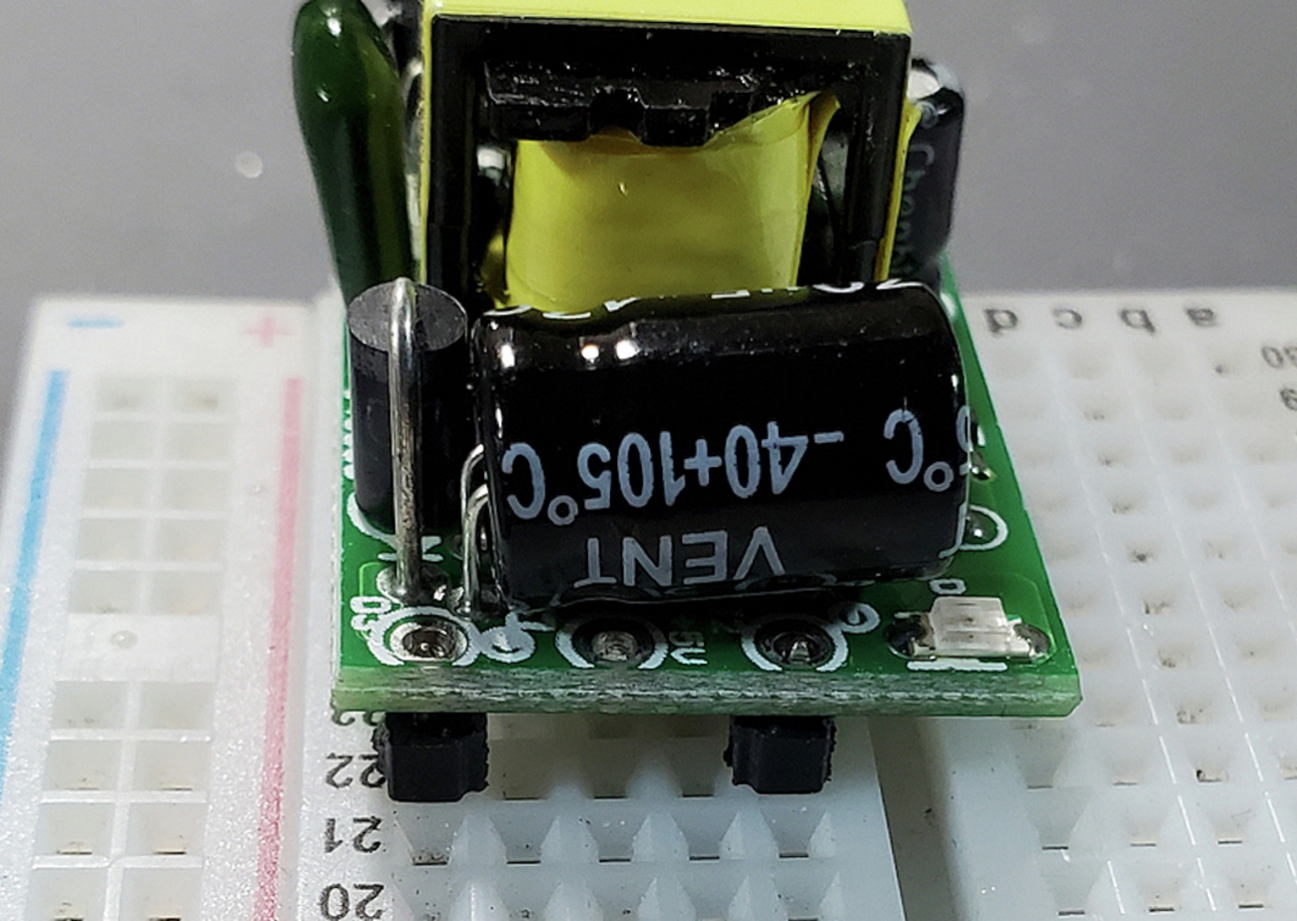
Buck converter DC pins (note unused center hole).

**Fig. 14:**
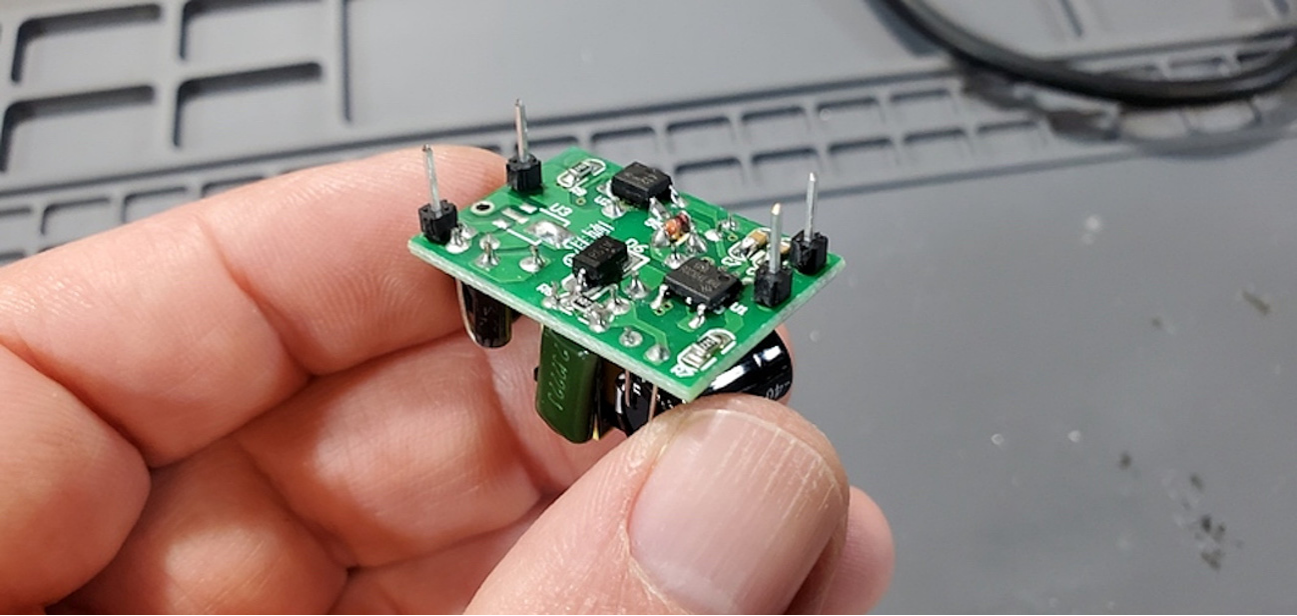
Back of Buck converter breakout board with four pins attached.

These components do not include pins so we will cut eight (four for each) from the 16x1 header (P16) as shown in Fig. 6 to be installed on the back (away from the transformer). At one end of the board there are two through-holes that are labeled on the back as “AC” while at the other end of the board there are three through-holes (all five holes might be pre-filled with solder). We want to attach pins to all except the middle hole in the three-hole set (Fig. 11, Fig. 12, Fig. 13, Fig. 14).

#### EZO™ Carrier Board (P10)

**Fig. 15:**
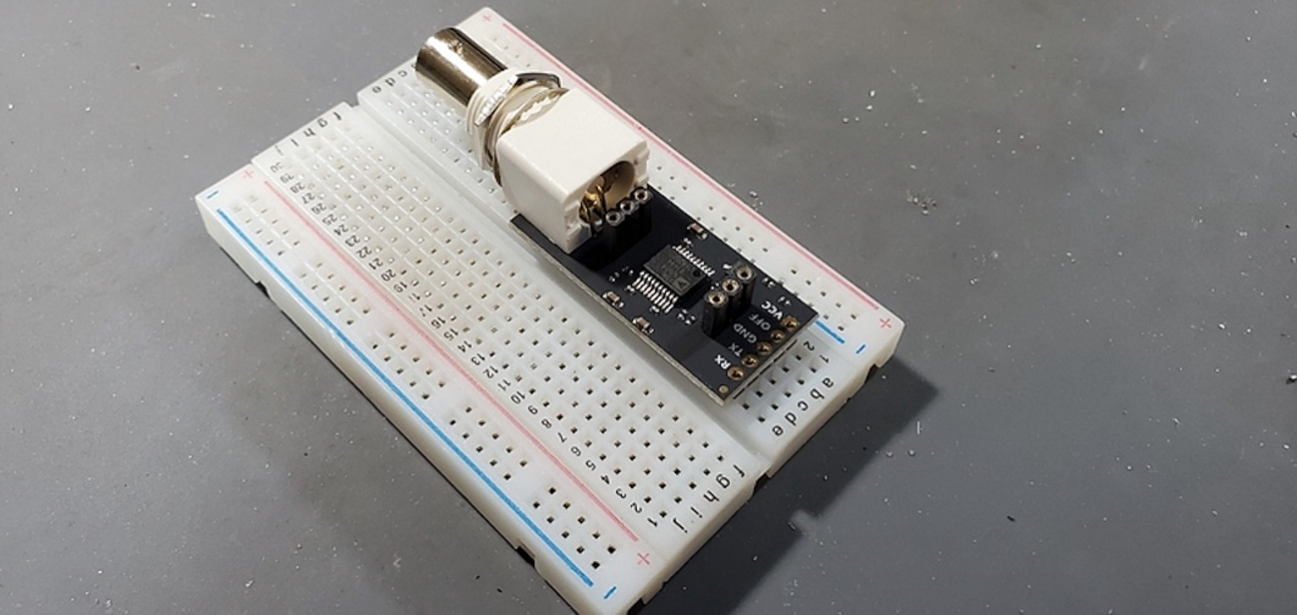
EZO™ Carrier Board with 5x1 pins attached to back.

This board includes a 5x1 male header that should be attached to the bottom of the board (Fig. 15).

**Fig. 16:**
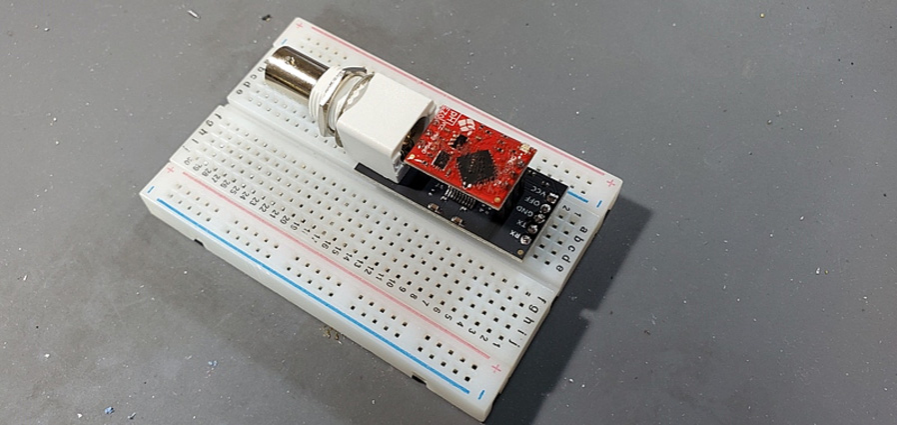
EZO™ circuit on the carrier board.

Place the red EZO™ circuit on the carrier board so that the “pH EZO” lettering is next to the large white plastic part of the carrier board and the BNC plug (Fig. 16).

#### Two-Channel DC 5 V Relay Module (P7)

**Fig. 17:**
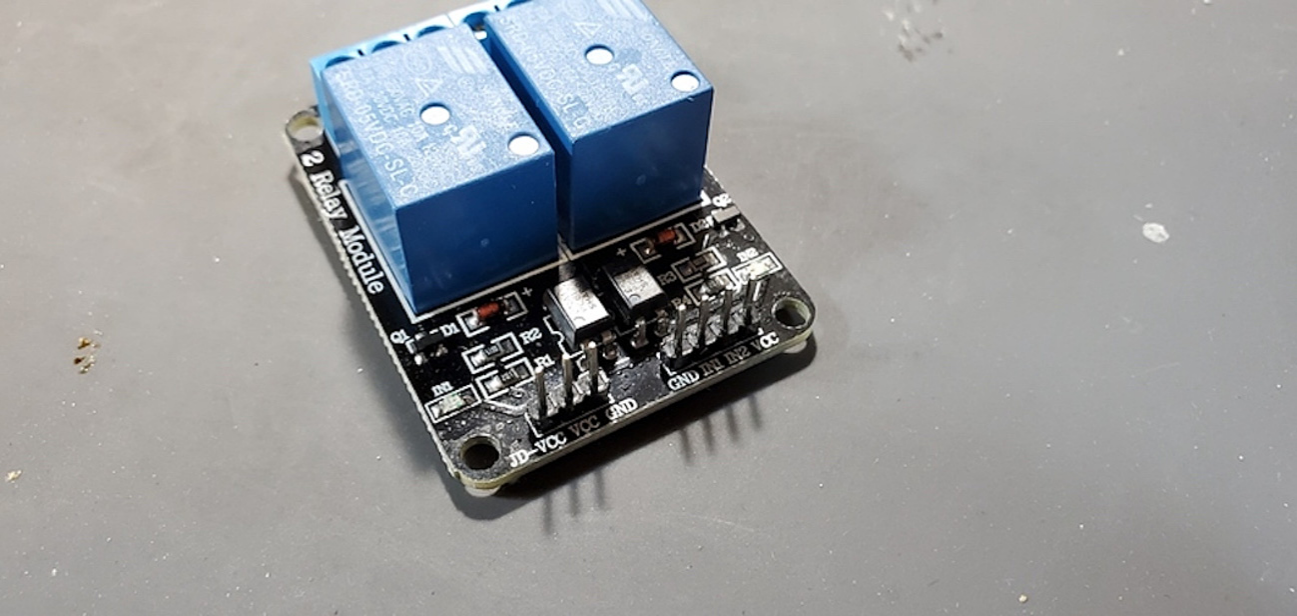
Two-relay module with pins facing up.

This module comes with seven pins pre-installed facing up when looking at the front of the breakout board (Fig. 17).

**Fig. 18:**
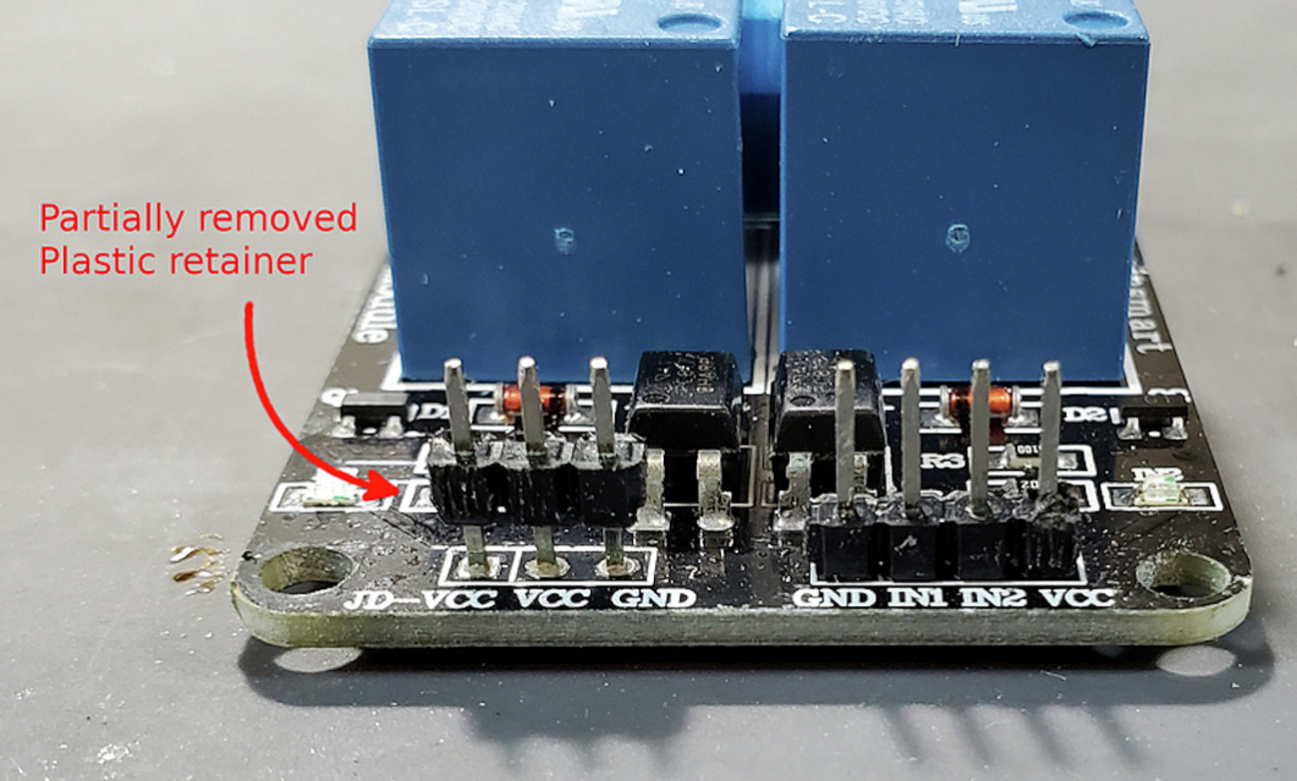
Relay module with plastic retainer partially removed.

Unfortunately, we want them facing down so will have to remove the existing pins and install new pins. Because the pins are joined by a plastic base, they will stay in place even when the solder on one of them is melted. So, we need to slide off or cut off the plastic base (Fig. 18).

**Fig. 19:**
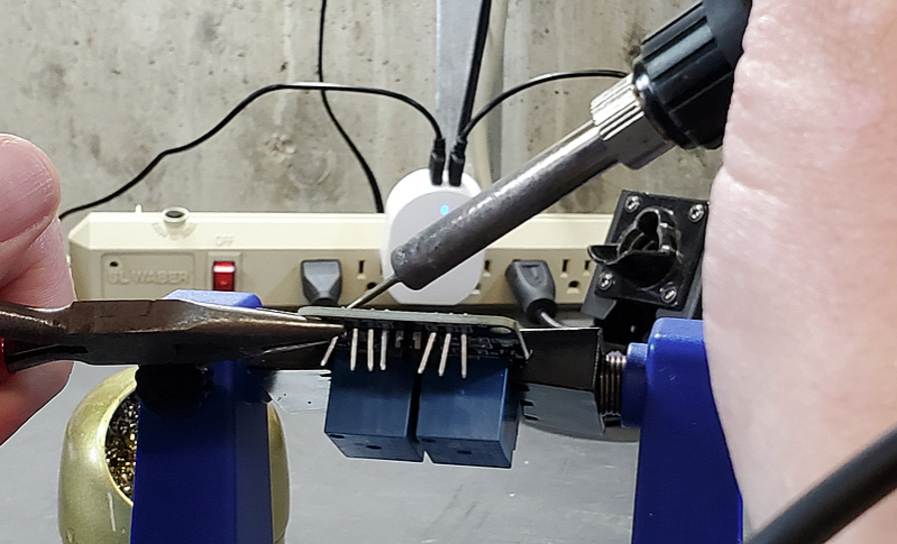
Removing pins from relay module.

Once the plastic retainer is removed, remove the pins by grasping the longer end of the pin with a pair of pliers and gently pull away from the board while heating the solder (Fig. 19).

**Fig. 20:**
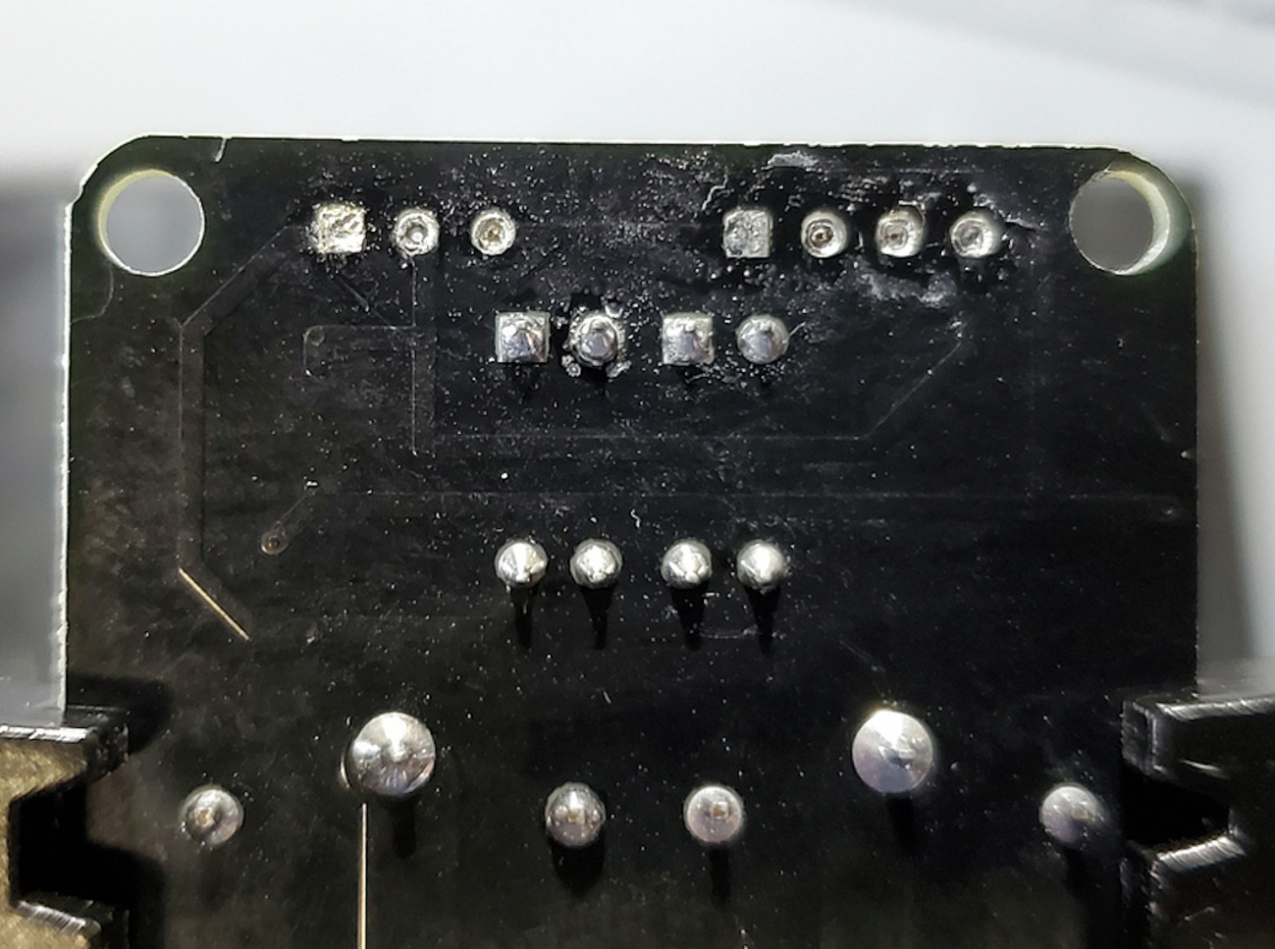
Relay breakout board with solder remnant in through-holes.

When you have completed this step, the through-holes on the breakout board will likely still be filled with remnant solder (Fig. 20).

**Fig. 21:**
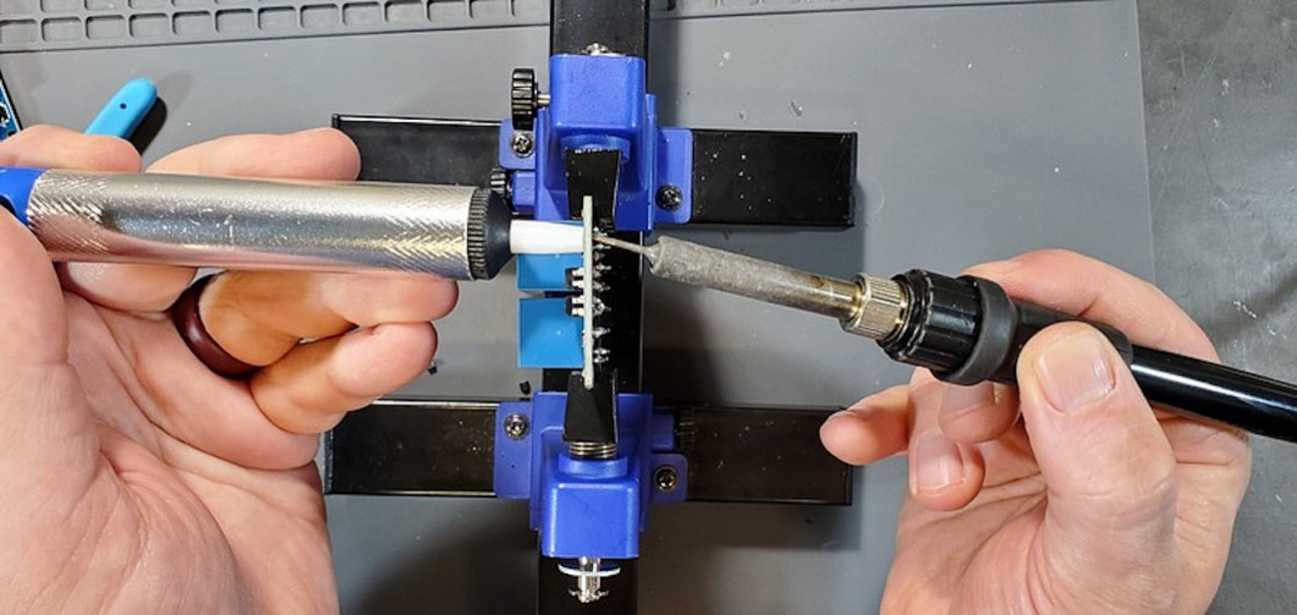
Removing remnant solder from relay breakout board.

Remove the solder from the through-holes by placing the aperture of the solder sucker directly over a through hole, while simultaneously heating the solder in the through hole with the soldering iron. When the solder liquefies, fire the solder sucker to remove the solder from the hole. You may need to repeat this step multiple times per through-hole to completely clear the hole of solder. Be careful not to leave the soldering iron on the through-hole too long and cause the pad to separate from the breakout board. You may need to remove solder from both sides (Fig. 21).

**Fig. 22:**
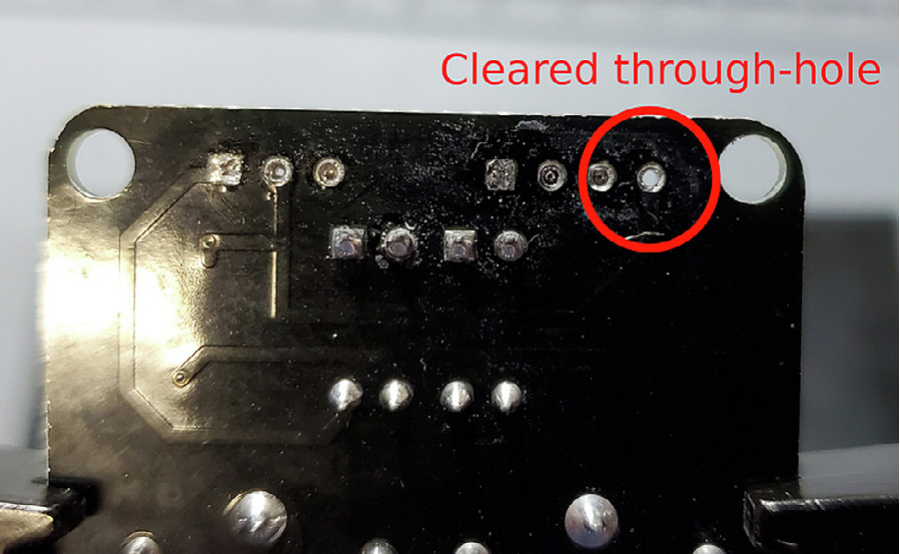
Cleared through-hole.

You can check whether the hole is sufficiently cleared by attempting to push the clean end of one of the removed pins through the joint. If you can push the pin through easily, it has been cleared. If it fits through, but tightly, you can spin the pin (which should be square in cross-section) in the hole to clear away the last few bits of solder (Fig. 22).

**Fig. 23:**
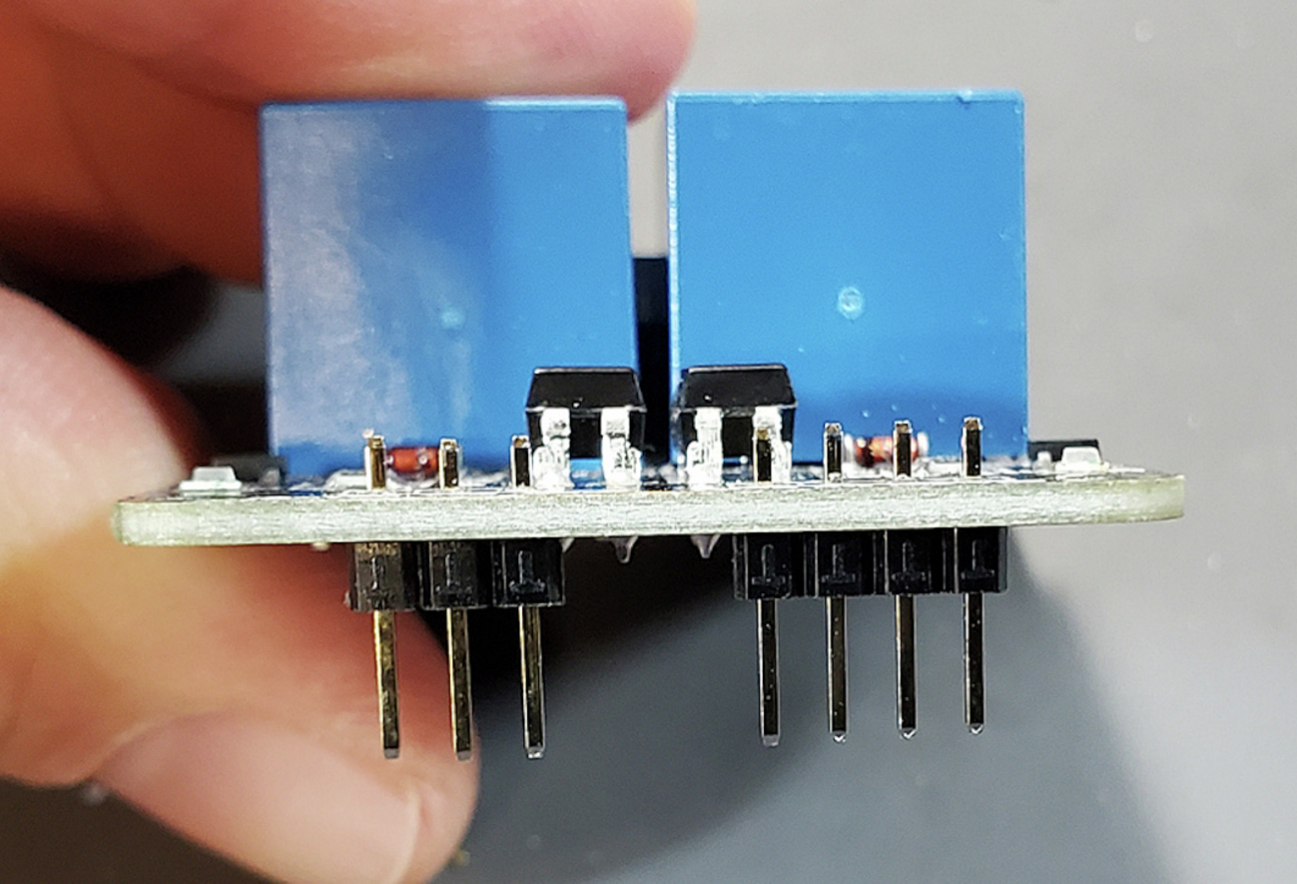
Relay module with pins facing down.

Finally, attach new 3x1 pins (P17) and 4x1 pins (P13) facing the back of the breakout board (soldering from the front). As before, this can be done using a breadboard (Fig. 23).

### Back of PCB

Next, we describe the components that are attached to the back of the custom PCB (which means they will be soldered from the front).

#### Arduino header pins

**Fig. 24:**
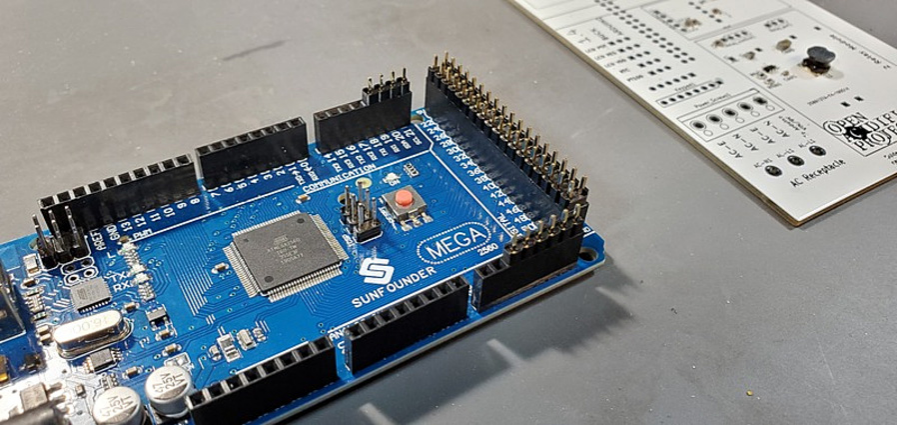
Arduino header pins placed in position.

The Arduino Mega 2560 connects to our custom PCB using 46 male pins: an 18x2 set (P12), a 4x1 set (P13), and a 6x1 set (P14). While this can be done using a breadboard (as before), we find it easier to use the Mega 2560 itself to position the pins. Insert the 4x1 pins into positions 18–21, the 6x1 pins into positions A10-15, and the 18x2 into positions 20–55 (Fig. 24).

**Fig. 25:**
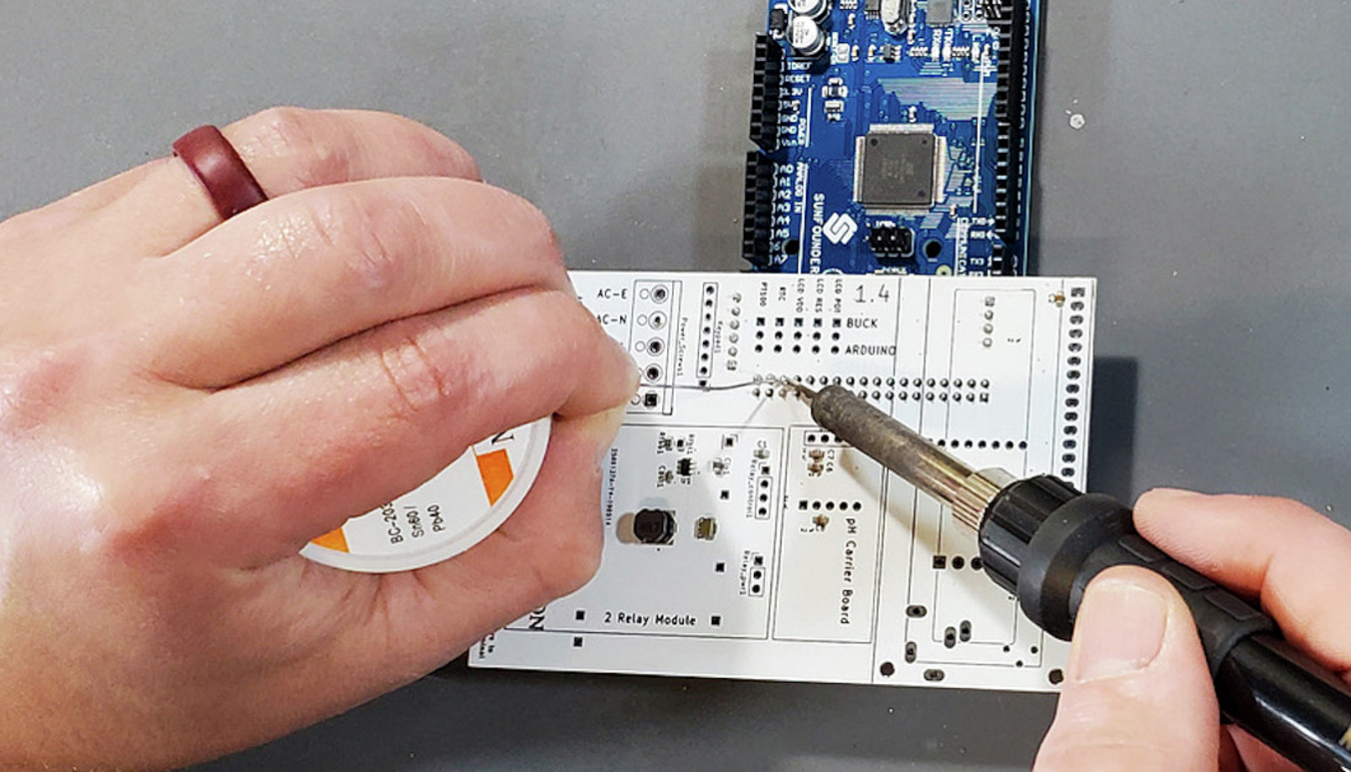
Arduino header pins being soldered.

**Fig. 26:**
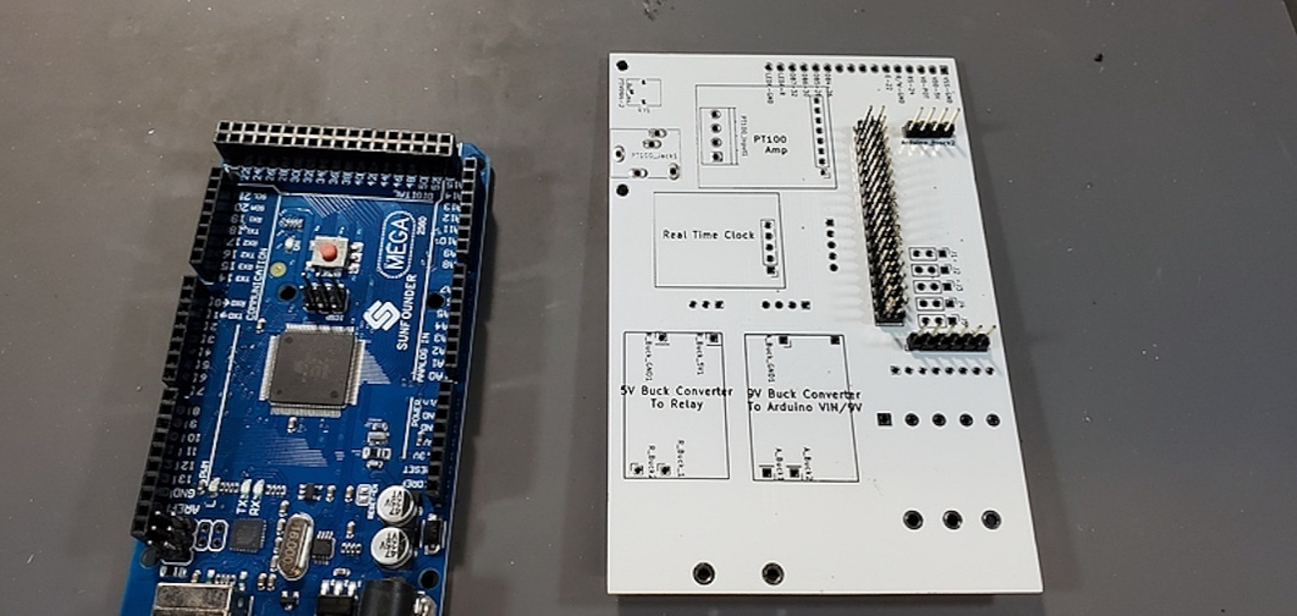
Arduino header pins attached to back of custom PCB.

Set the custom PCB onto the pins so that the pins are facing the back of the custom PCB and the front of the custom PCB is available to solder the pins (Fig. 25, Fig. 26).

#### Potentiometer (P28)

**Fig. 27:**
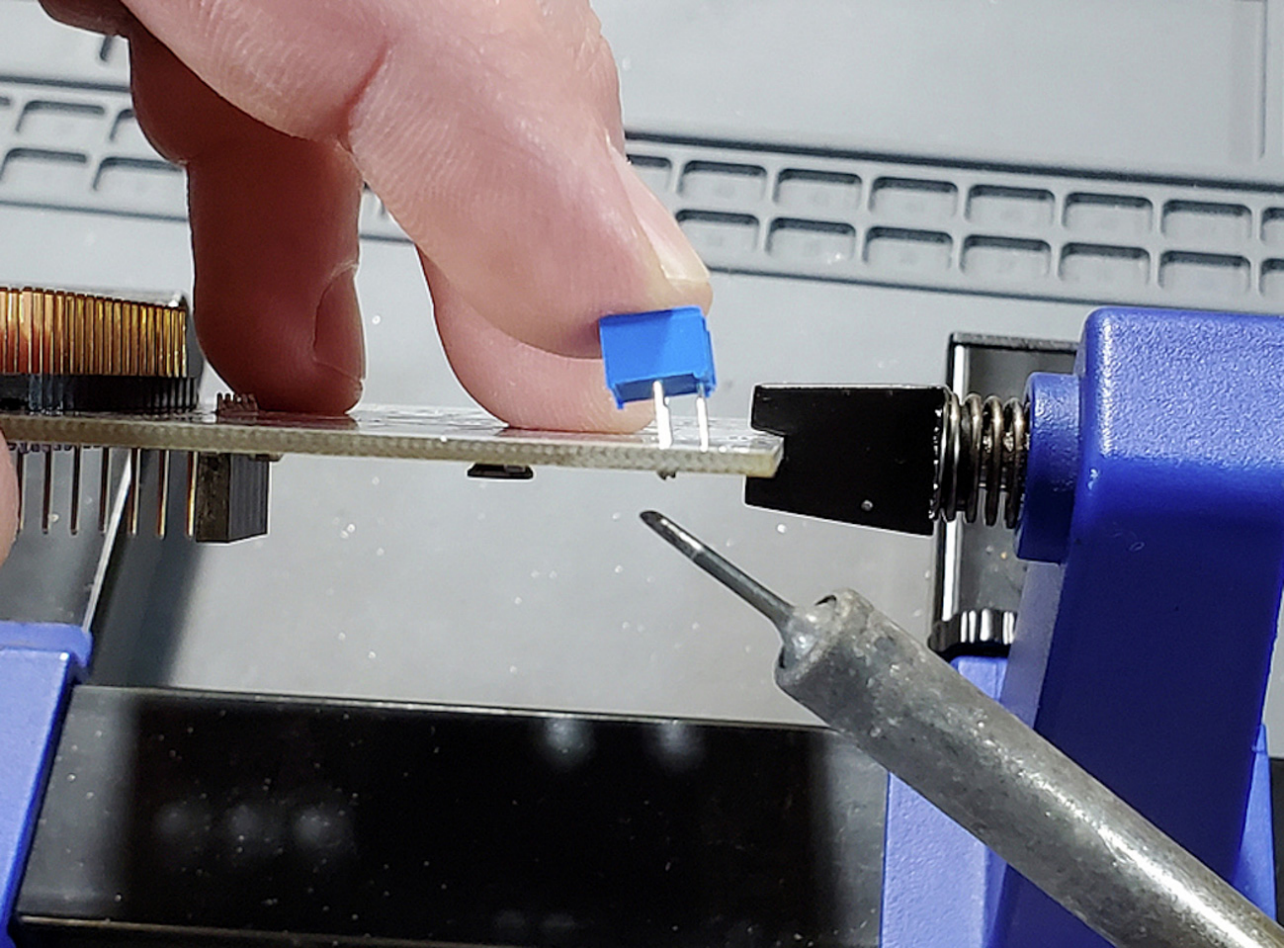
Potentiometer on the back of the custom PCB.

The potentiometer is attached to the back of the custom PCB (this is used to set the backlight level for the LCD; Fig. 27).

#### 3.5 mm jack receptacle (P27)

**Fig. 28:**
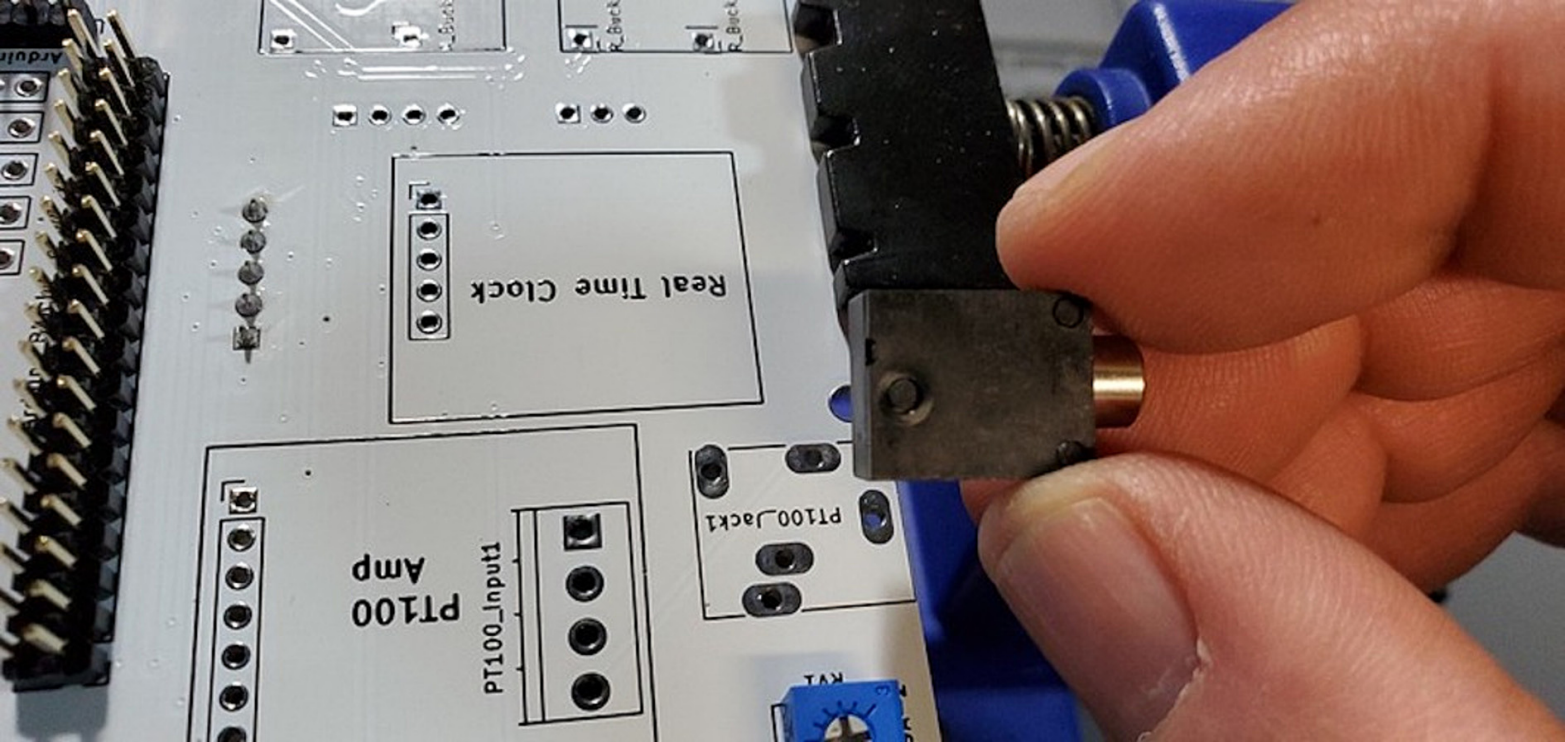
Temperature probe connector jack placement.

**Fig. 29:**
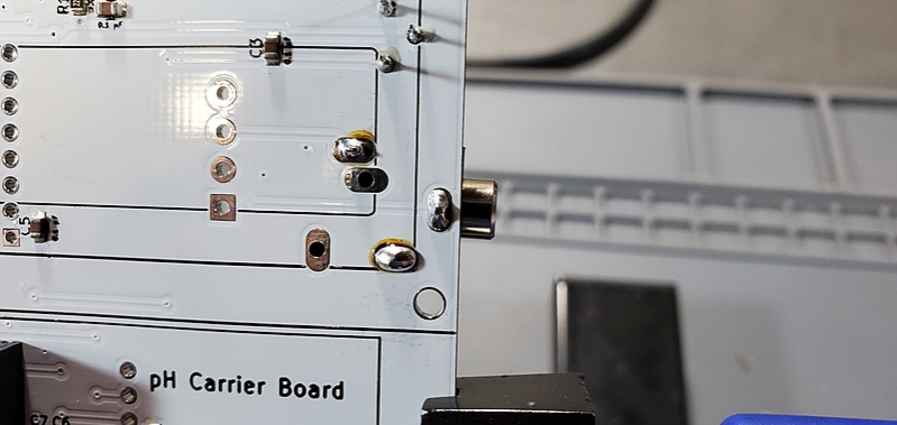
Temperature probe connector jack soldered in place.

This ⅛” mini stereo headset jack is used to connect to the temperature probe. Although the custom PCB has five through-holes, the jack should have only three terminals or pins. Position the jack on the back of the custom PCB (Fig. 28) and solder it in place (Fig. 29).

#### Real time clock 5x1 female header (P24)

**Fig. 30:**
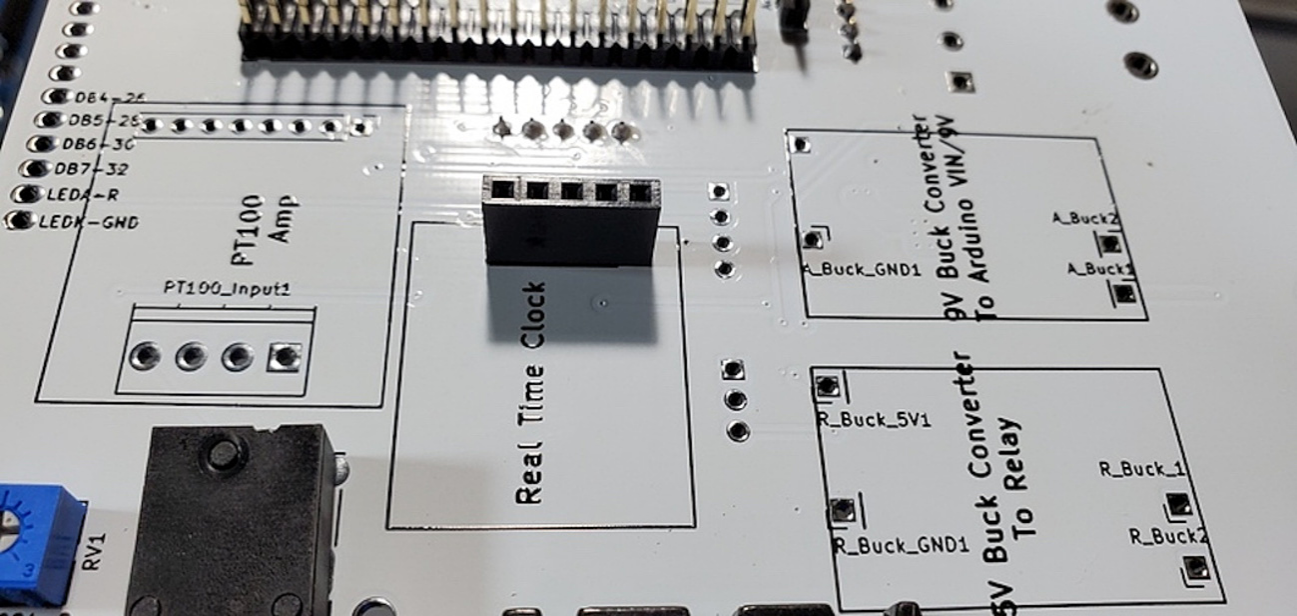
Real time clock socket attached to the back of the custom PCB.

Attach the 5x1 female socket to the back of the custom PCB in the spot labeled “Real Time Clock”. To attach the socket, you can fill one through-hole with solder (perhaps using a PCB holder), then gently press the pins into position while melting the solder from the opposite side. When the solder cools it should hold the socket in place for you to solder the other pins (Fig. 30).

#### Max 31865 PT100 RTD converter 5x1 female socket (P24) and three 1x1 (P26)

The female header sockets are placed on the back of the custom PCB in the space labeled “PT100 Amp”. The three 1x1 sockets are placed in the three circular through-holes (leave the square through-hole blank. An easy way to do this is to attach the female header sockets to the male pins already soldered to the breakout board and then hold the breakout board against the through-holes (Fig. 7). Solder all eleven pins from the front of the custom PCB (Fig. 9).

### Front of PCB

Next, we describe the components that are attached to the front of the custom PCB (which means they will be soldered from the back).

#### pH Carrier board 5x1 female socket (P24)

**Fig. 31:**
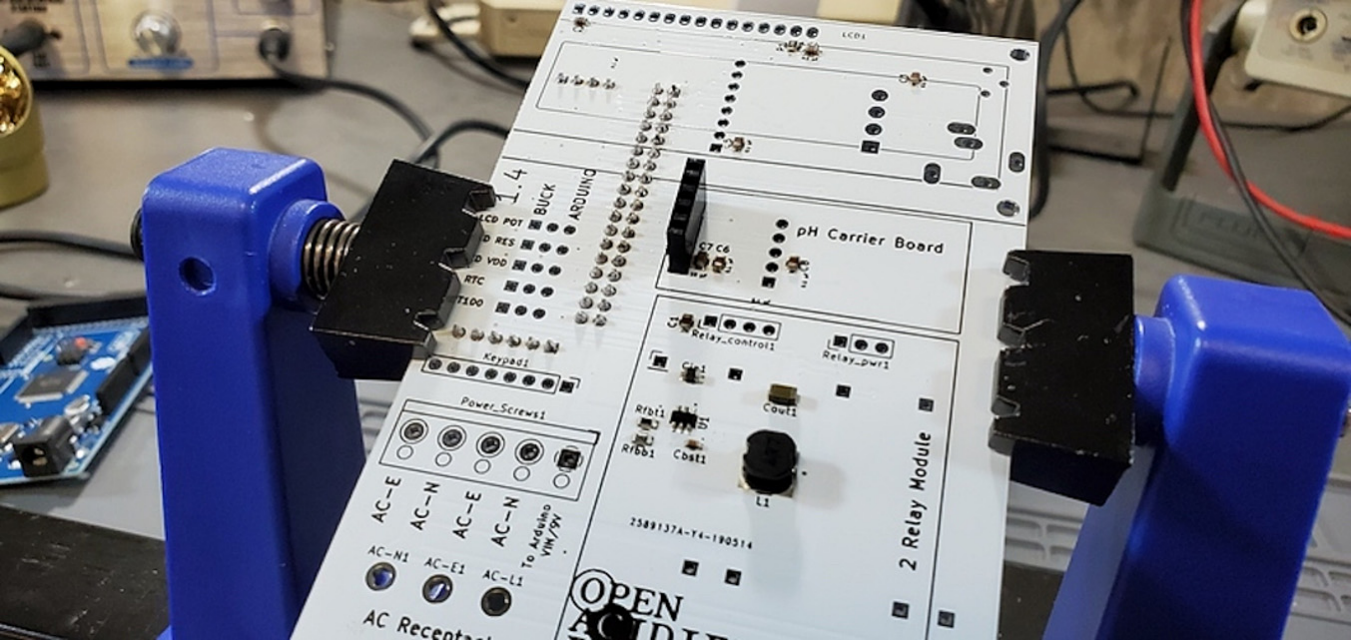
5x1 female socket for pH carrier board.

Attach a 5x1 female socket to the front of the board in the space labeled “pH_carrier1” (Fig. 31).

#### Keypad 8x1 male pins (P15)

**Fig. 32:**
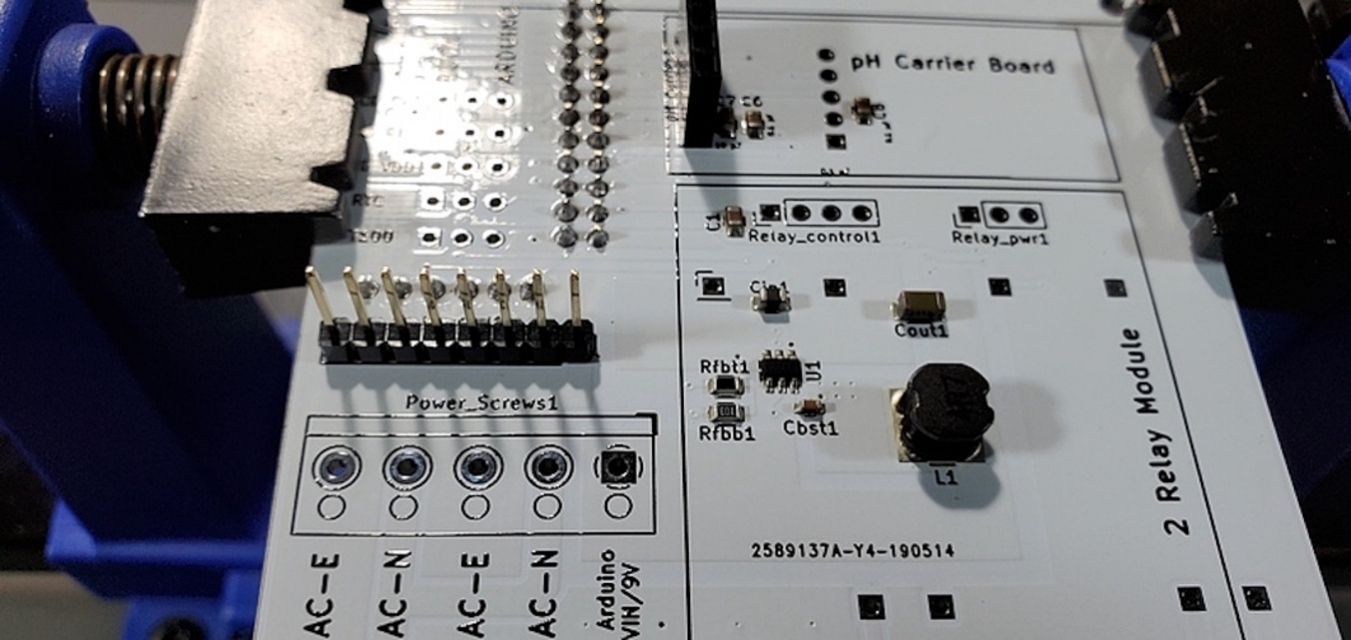
8x1 male pin set for keypad.

Attach an 8x1 male pin set to the front of the board in the space labeled “Keypad1” (Fig. 32).

#### 16x1 female socket for LCD (P20)

**Fig. 33:**
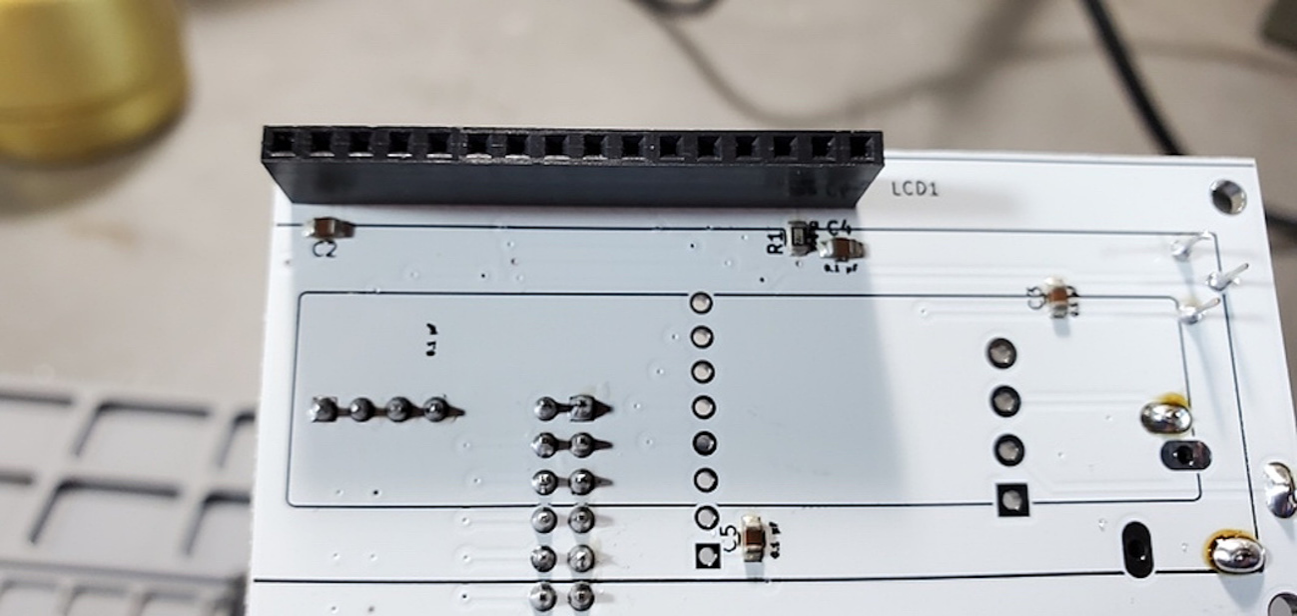
16x1 female socket for LCD.

Attach a 16x1 female socket to the front of the custom PCB next to the space labeled “LCD1” (Fig. 33).

#### 5-Position Wire-to-Board Terminal Block (P11)

**Fig. 34:**
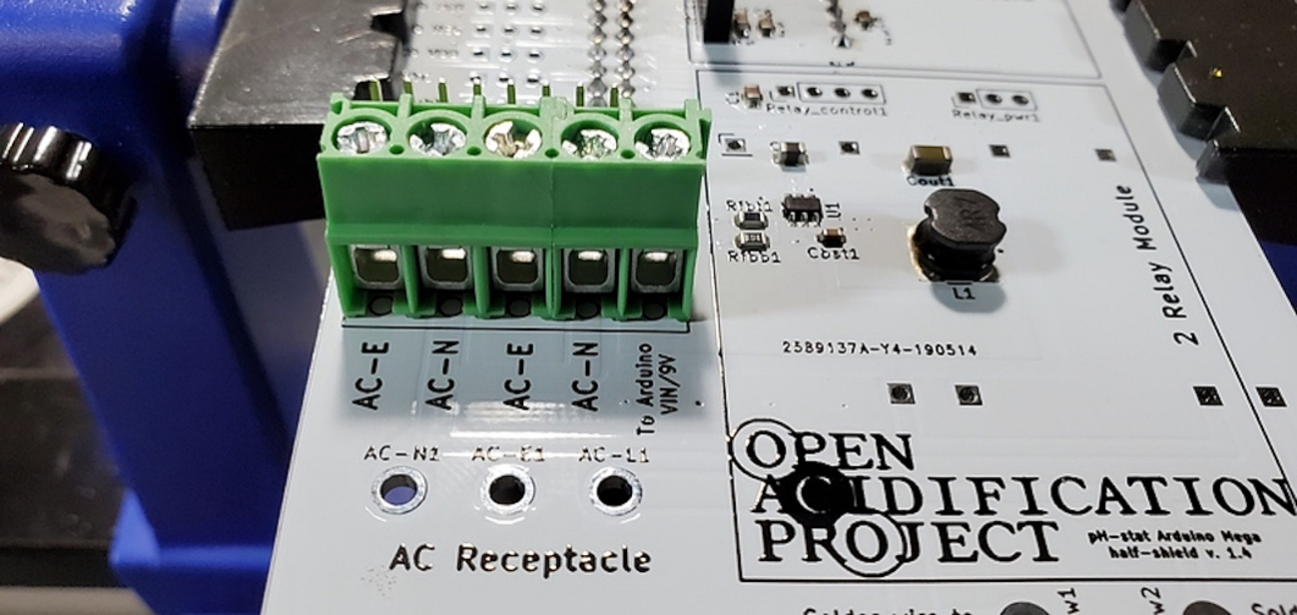
Terminal block.

Attach the terminal block to the front of the custom PCB in the space labeled “Power_Screws1”. The openings on the side of the terminals (where the wires will be fed) should be facing toward the “AC Receptacle” location on the PCB. (Fig. 34)

#### Five 3x1 Male Jumper Pins (P17)

**Fig. 35:**
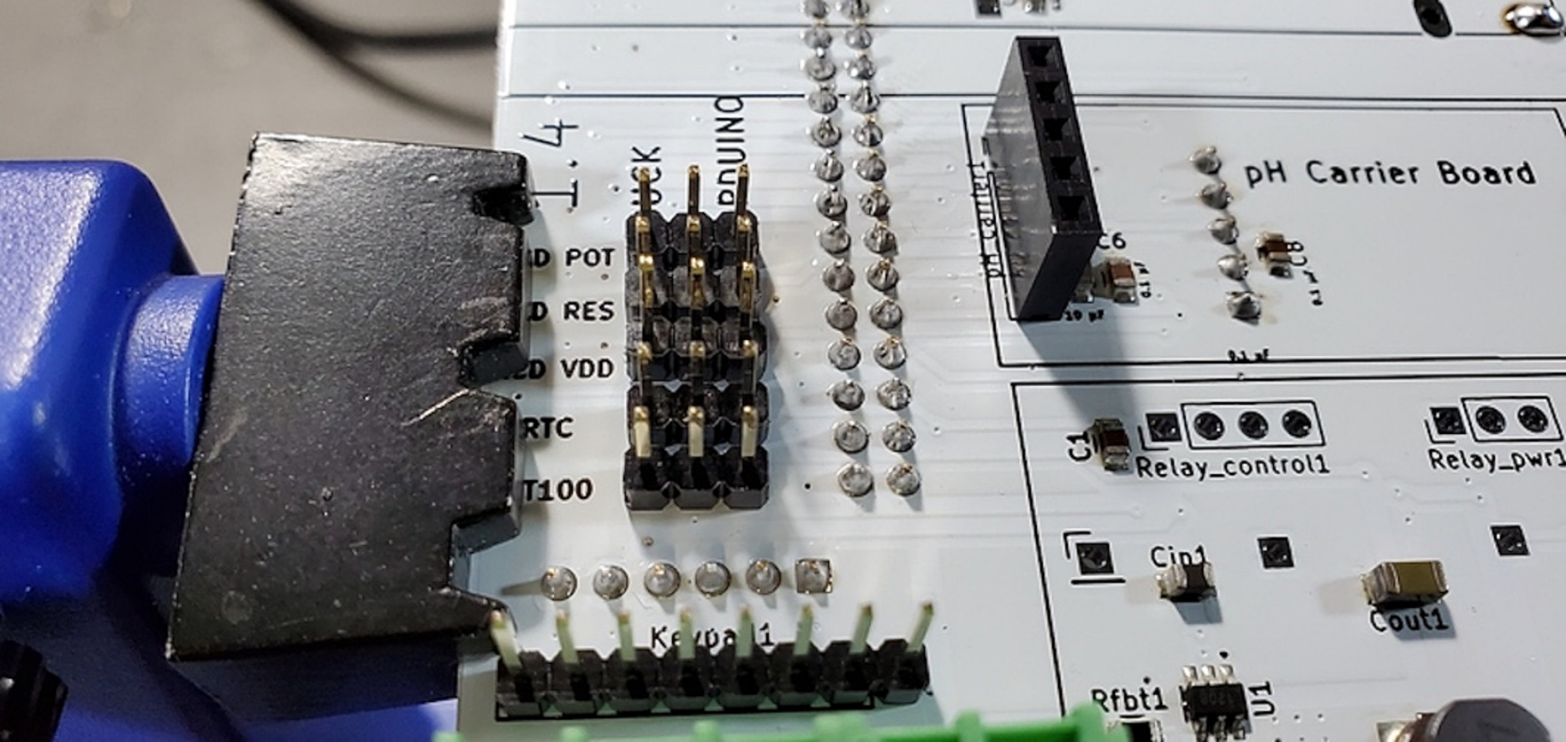
Jumper pins.

Attach five sets of 3x1 male pins to the front of the custom PCB in the spaces labeled “LCD POT”, “LCD RES”, “LCD VDD”, “RTC”, and “PT100” (Fig. 35).

**Fig. 36:**
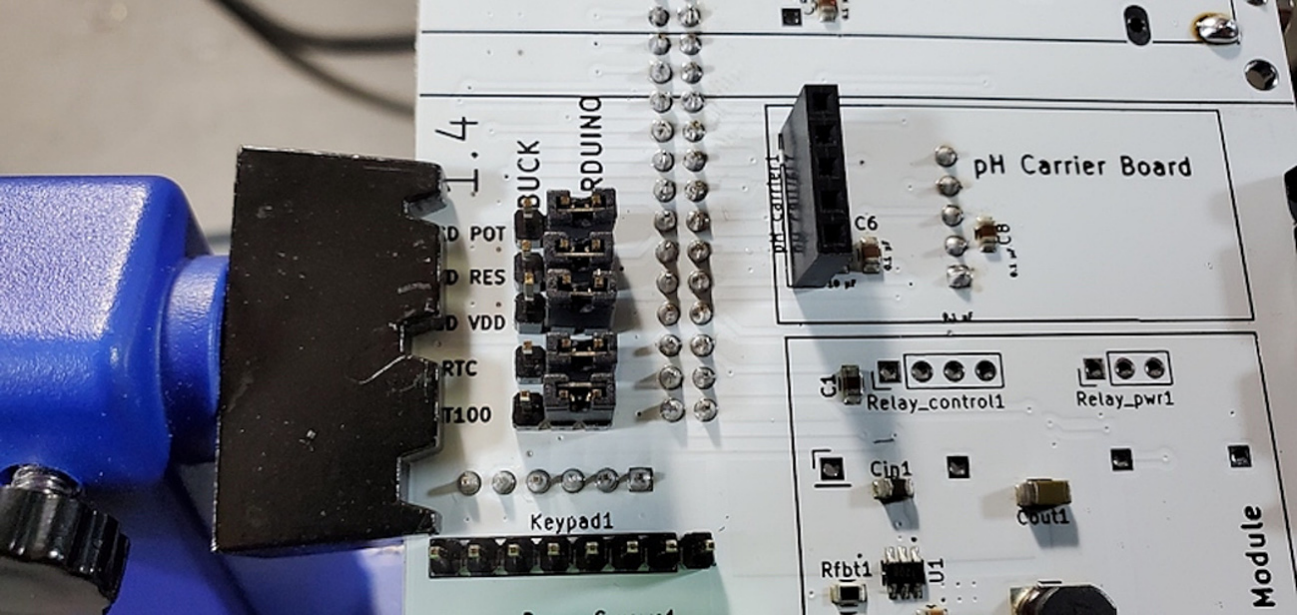
Jumpers on jumper pins.

Once they are soldered on, place jumpers (P50) between the center pin and the “Arduino” pin on each header (Fig. 36).

#### AC Power Receptacle (P18)

**Fig. 37:**
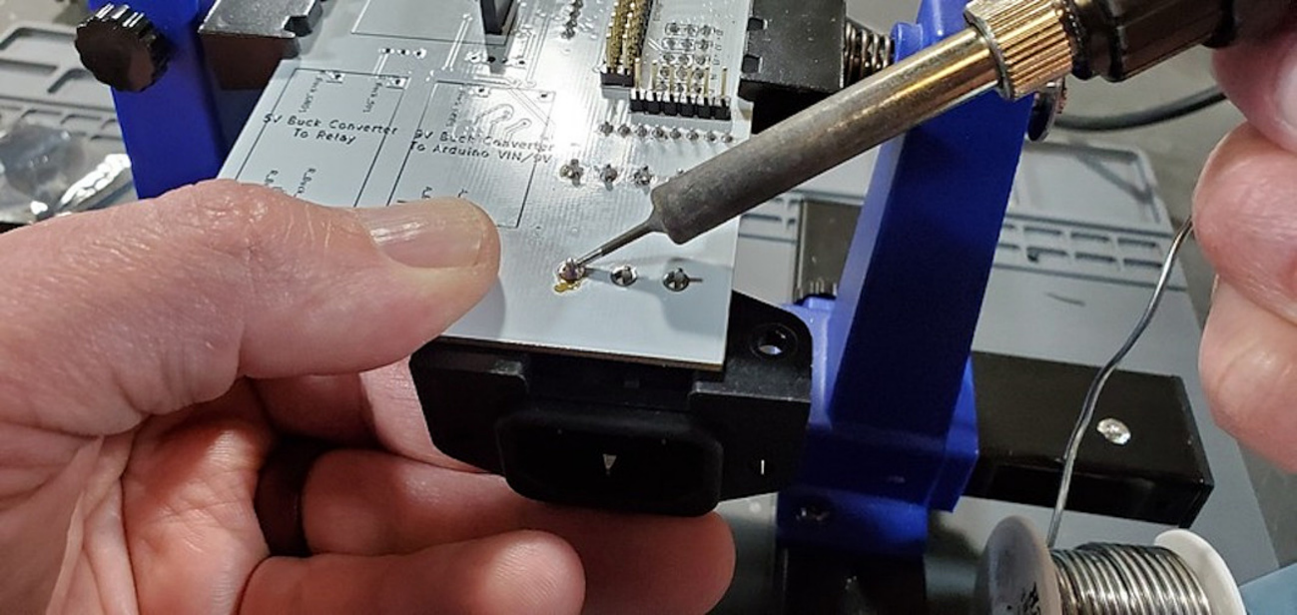
Attaching the AC power receptacle.

Attach the AC power receptacle to the front of the custom PCB in the space labeled “AC Receptacle”. A convenient way to do this is to turn the board over (facing the back), hold the receptacle from underneath, place a small chunk of solder near one of the terminals, and then use that solder to attach the receptacle (Fig. 37).

#### Female Sockets for Relay Module: 4x1 (P22) and 3x1 (P23)

**Fig. 38:**
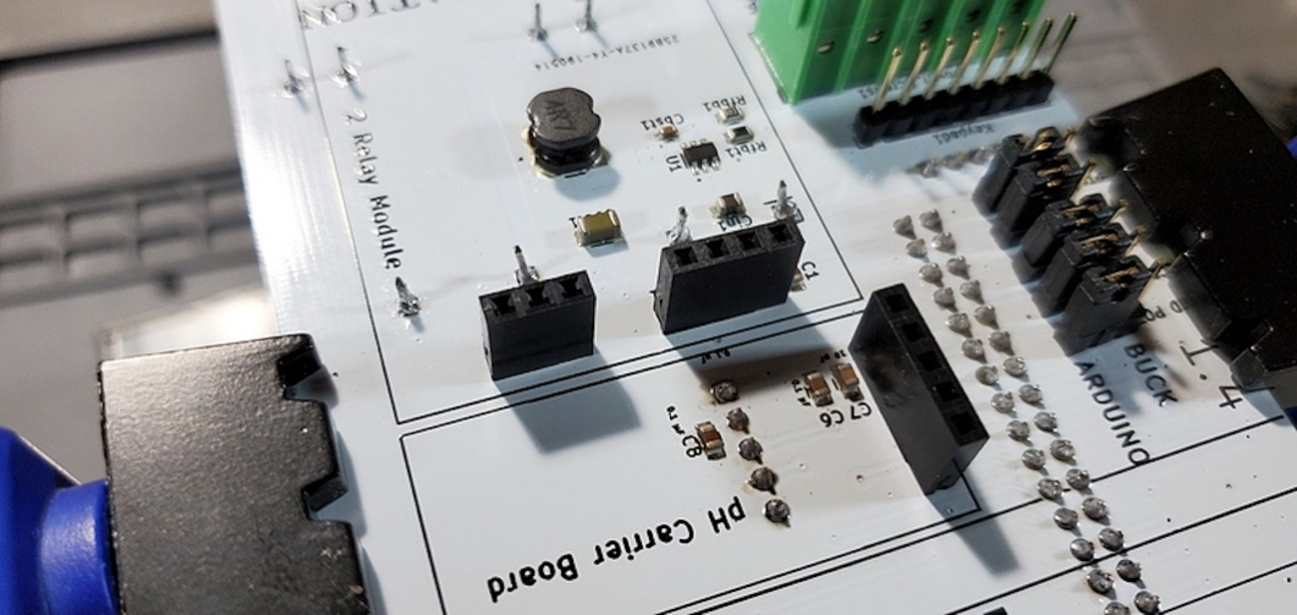
Female sockets for relay module.

Attach a 4x1 female socket to the front of the custom PCB in the space labeled “Relay_control1” and attach a 3x1 female socket in the space labeled “Relay_pwr1” (Fig. 38).

### Buck converters and power

Next, we describe attaching the buck converters and wires for the AC power.

#### Buck Converter Modules (P40 and P41)

**Fig. 39:**
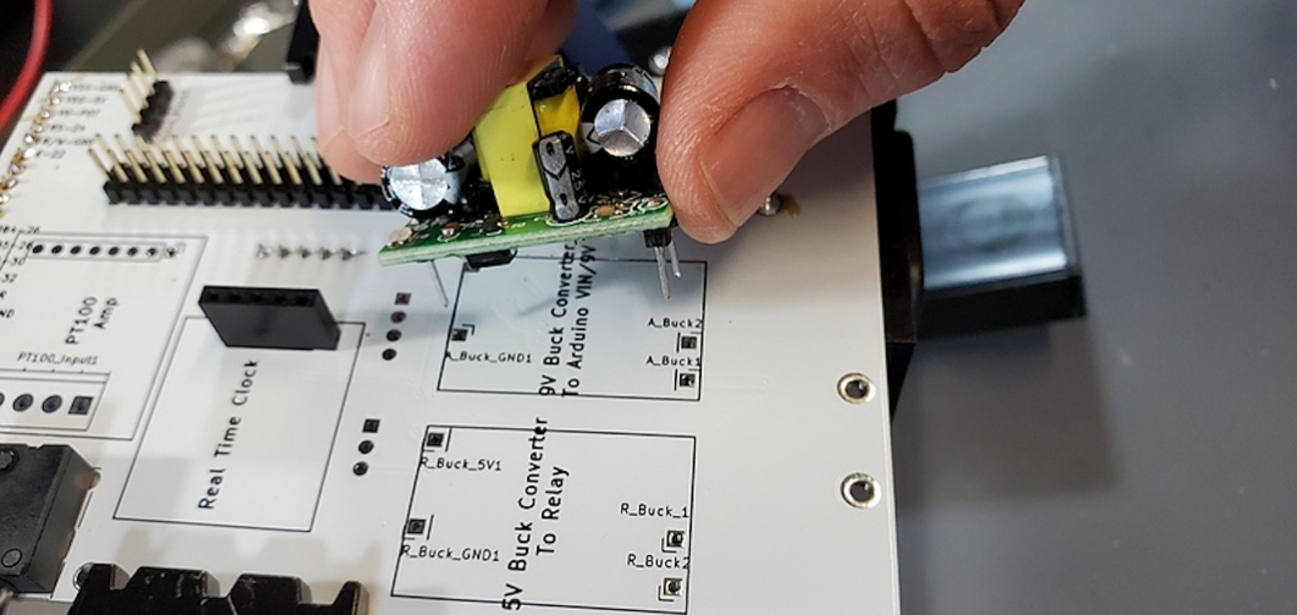
Buck converter placement.

Place the buck converters onto the back of the custom PCB. The through holes for these converters are slightly closer together on the PCB than on the converters. This is intentional so that you can place them and have tension keep them in place while you test the units before soldering them on. Make sure that you place the correct buck converter in the correct place on the PCB. The positions are marked on the PCB. The 5 V buck converter should have a 1000 μF capacitor on the output end, the 9 V should have a 470 μF capacitor in the same position (Fig. 39).

Before soldering the buck converters to the custom PCB, you should test the outputs to make sure (1) that you placed the buck converters in the correct positions and (2) that the converters are not defective (the Amazon reviews include several comments about defective units). To do this we need to plug the board into an outlet (P48), which must be done very carefully to prevent giving yourself a very dangerous shock. To do this safely, first secure the PCB so that it remains in place while plugged in (we suggest using a PCB holder for this). Then, plug the power cable into the PCB. Only then plug the power cable into a wall outlet. Once the power cable is attached, DO NOT TOUCH the custom PCB except with a multimeter. If you need to reposition the PCB, unplug it from the wall before doing so.

**Fig. 40:**
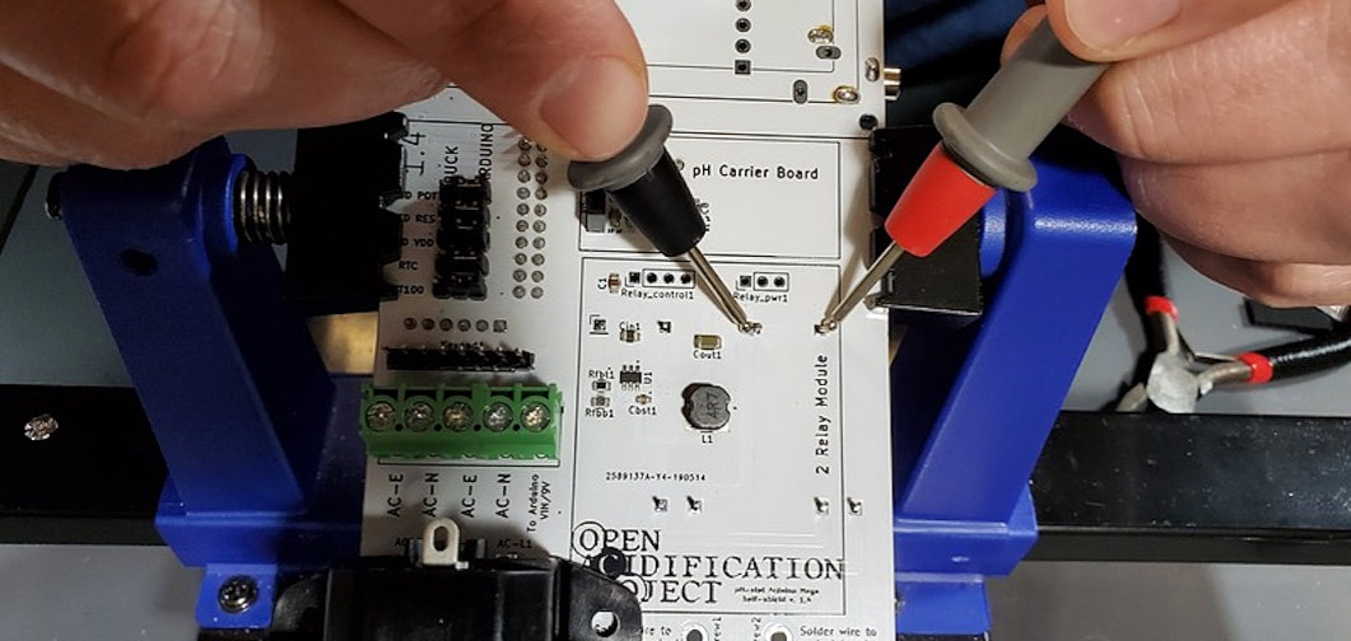
Checking the voltage on the buck converters.

Once you have the PCB secured and plugged in, use a multimeter to check the voltage output on both the 5 V and the 9 V buck converters. This is most easily done on the pins protruding through the front of the custom PCB (Fig. 40).

**Fig. 41:**
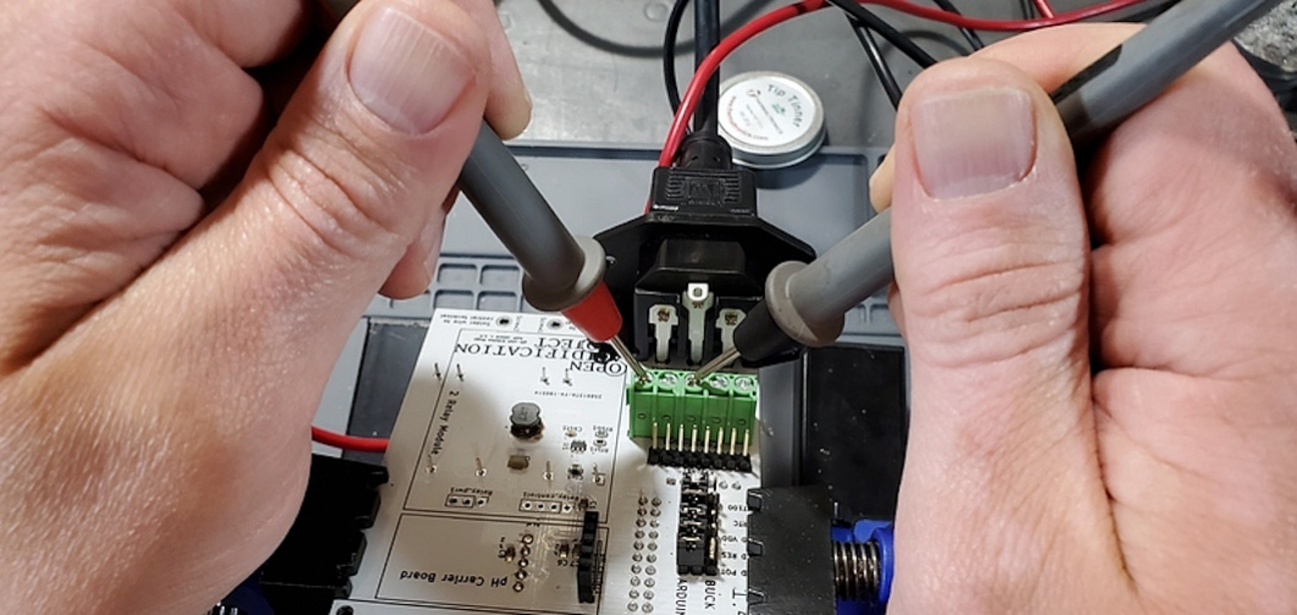
Checking the voltage on the Arduino.

It is also useful to check the 7 V power output from the screw terminal block for the Arduino. You can do this by checking voltage between the screws labeled “To Arduino VIN/9V” and “AC-E” (Fig. 41).

Once you have checked the voltage outputs of the buck converters, confirming correct placement and that they are in working order, you can solder down those two devices. Before you do this, however, UNPLUG THE POWER. After you have soldered all 4 pins on each buck converter, you can trim the pins with a wire cutter.

#### Attach the Two-Channel Relay Module (P7)

**Fig. 42:**
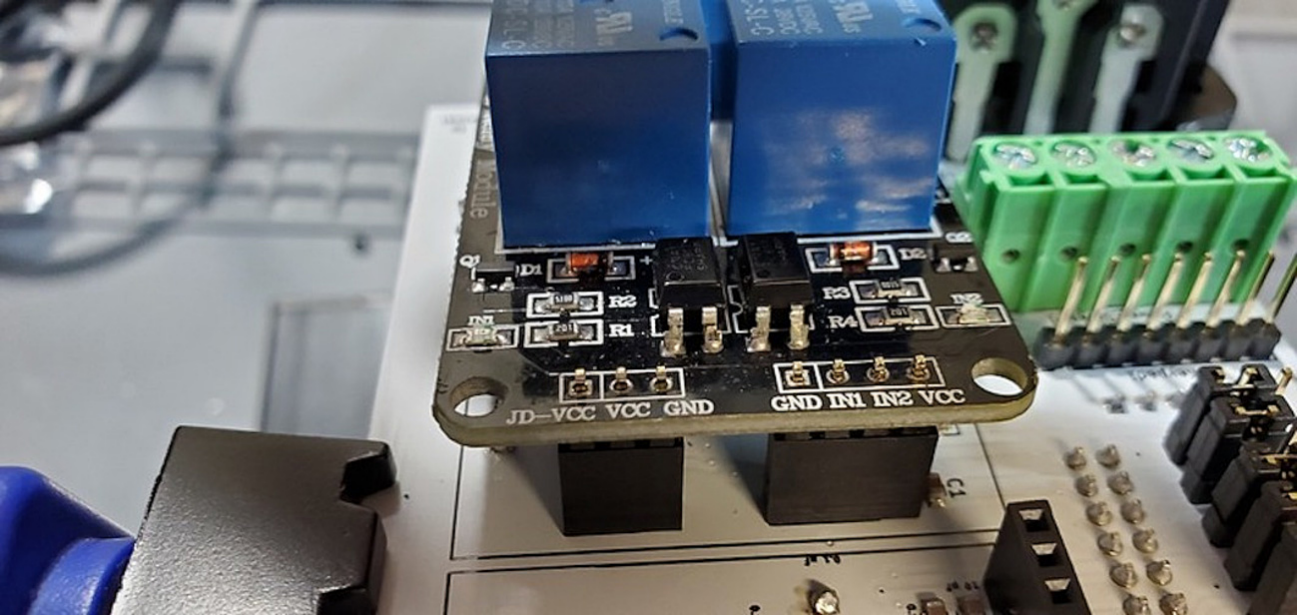
Two-channel relay module attached to front of custom PCB.

Insert the two-channel relay module (Fig. 23) into the appropriate female headers (Fig. 42).

**Fig. 43:**
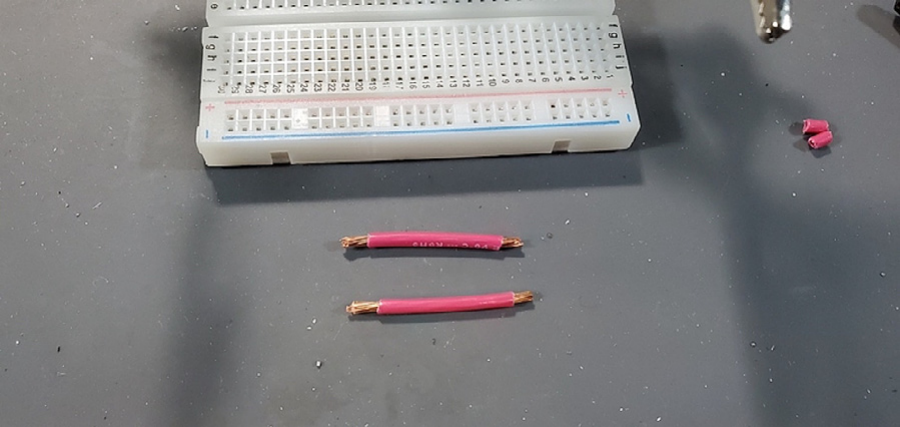
Wires cut and stripped.

Cut two pieces of stranded 14-gauge (AWG) wire (P46) to a length of approximately 3.5 cm and strip the insulation off the last 5 mm of each end (Fig. 43).

**Fig. 44:**
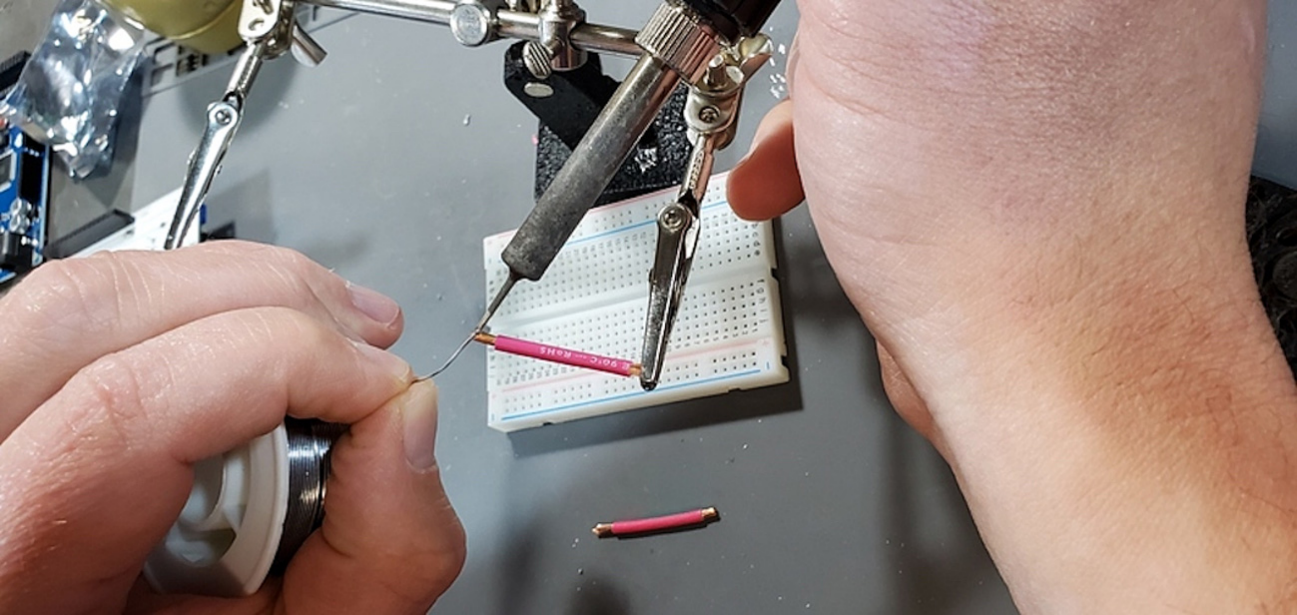
Tin the ends of the stripped wires.

Tin the ends of the wire where it is not covered by insulation. This involves heating the bare wire with the soldering iron, and melting solder into the wire, allowing the strands to “wick” up the solder (Fig. 44).

**Fig. 45:**
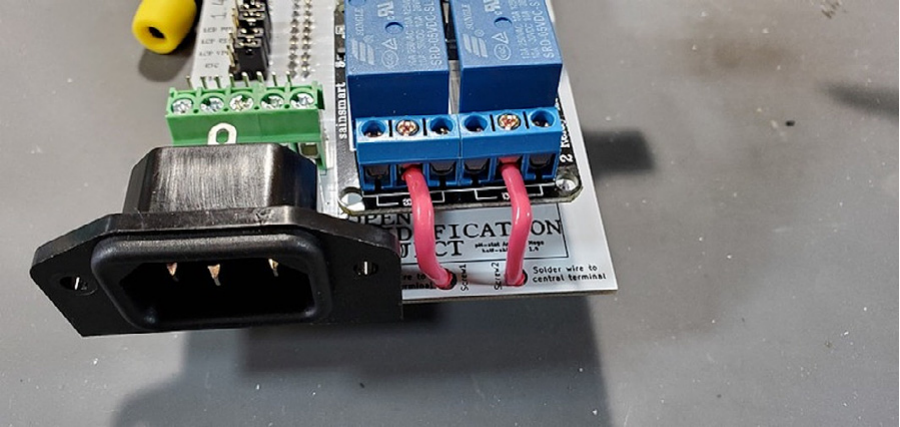
Wires screwed to relays.

Bend the wires about 90 degrees approximately in the middle. Place one end of the wire in the middle terminal of each of the two 3-position terminal screw blocks on the relay module and place the other sides in the hole in the PCB labeled “Screw1” and “Screw2”. Tighten the screws to secure the wires into place (Fig. 45).

**Fig. 46:**
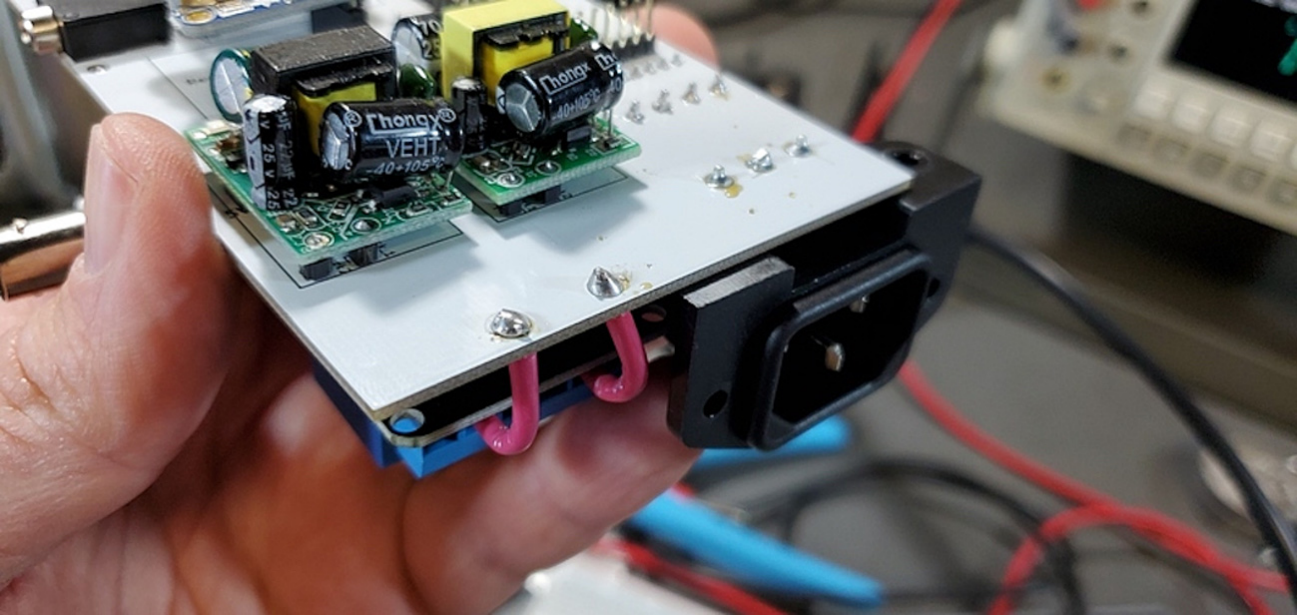
Wires soldered to custom PCB.

Finally, solder the ends of the wires in the PCB to the holes (Fig. 46).

#### Insert Three Modules

• Insert the Real Time Clock breakout board on the back of the custom PCB in the spot labeled “Real Time Clock”.
• Insert the Max31865 RTD converter on the back of the custom PCB in the spot labeled “PT100 Amp”.
• Insert the pH Carrier Board on the front of the custom PCB in the spot labeled “pH Carrier Board”.

Now it is time to assemble the Tank Controller.

### Faceplate Assembly

#### Parts and Tools (Fig. 47)

• 3D printed faceplate

**Fig. 47:**
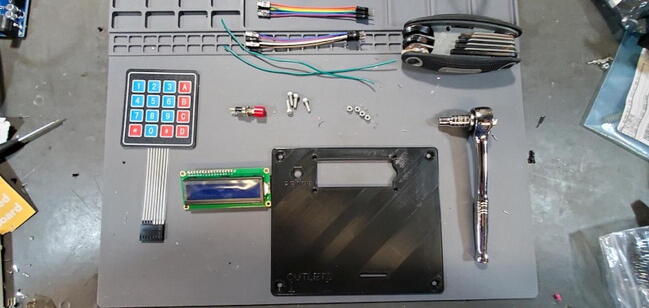
Faceplate parts.
• LCD module
• 2x 6-pin female-male jumper cables
• 4x4 matrix keypad
• Button
• 2x 20 cm solid core 22 AWG wire
• 4x M3 × 0.5 Socket hex head bolts
• 4x M3 nuts
• Metric 2.5 Allen wrench
• 5.5 mm socket wrench

#### Attach Reset Button (P29)

**Fig. 48:**
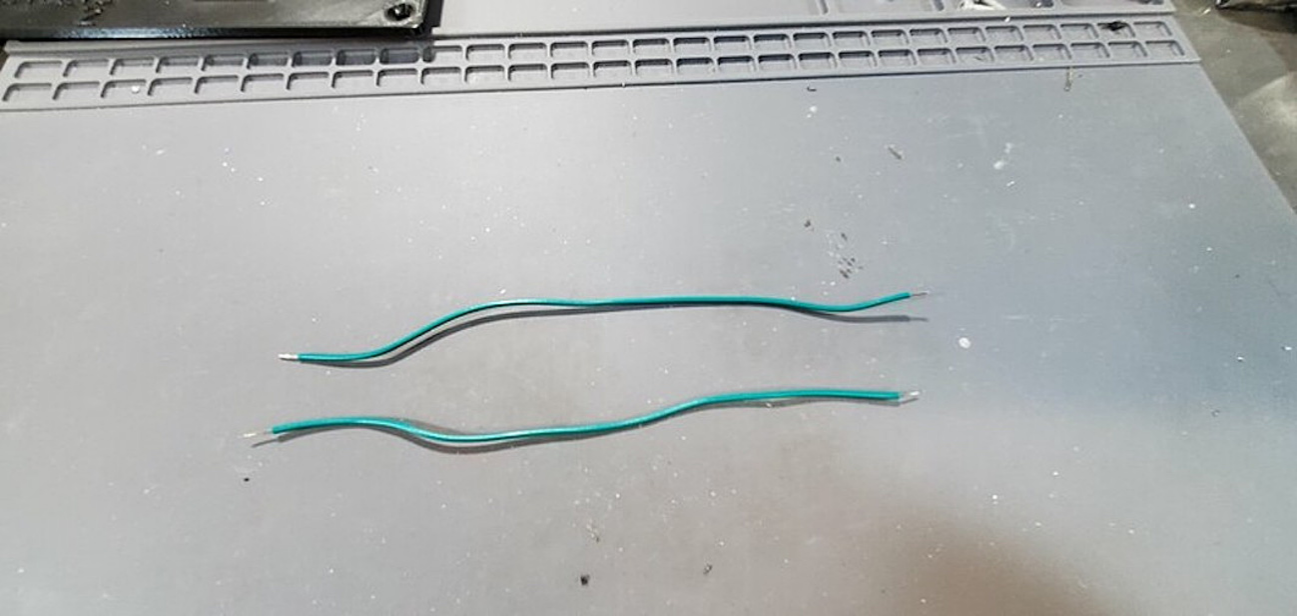
Reset wires.

Cut two sections of solid core 14-gauge wire (P46) to 20 cm. Strip the insulation from ∼ 1 cm from each end of each wire (Fig. 48).

**Fig. 49:**
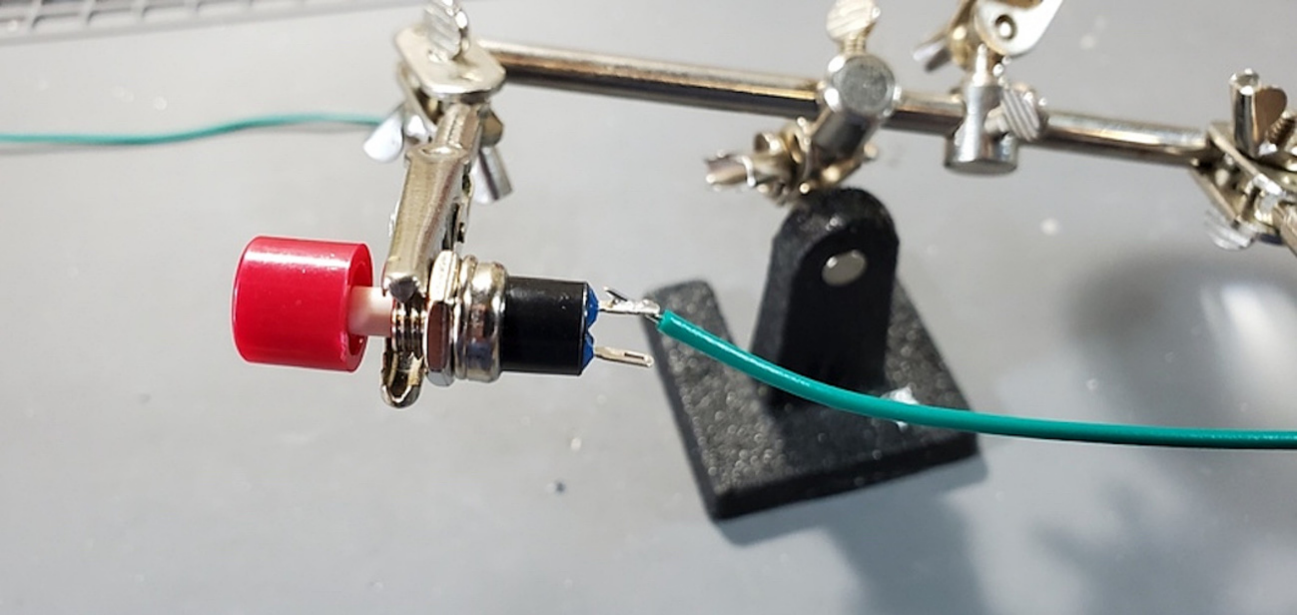
Wire soldered to reset button.

Solder one end of each wire to the terminals of the reset button (P29; Fig. 49).

**Fig. 50:**
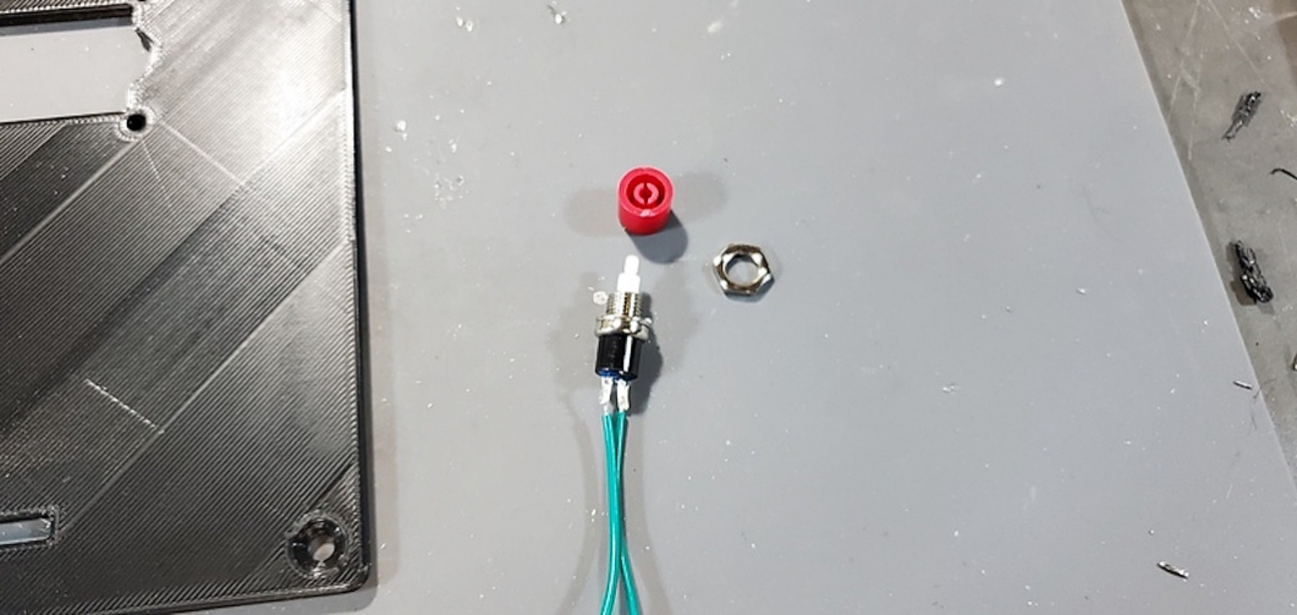
Reset button disassembled.

Disassemble the button pulling the red button cap from the button pin. Then, unscrew the nut from the button (Fig. 50).

**Fig. 51:**
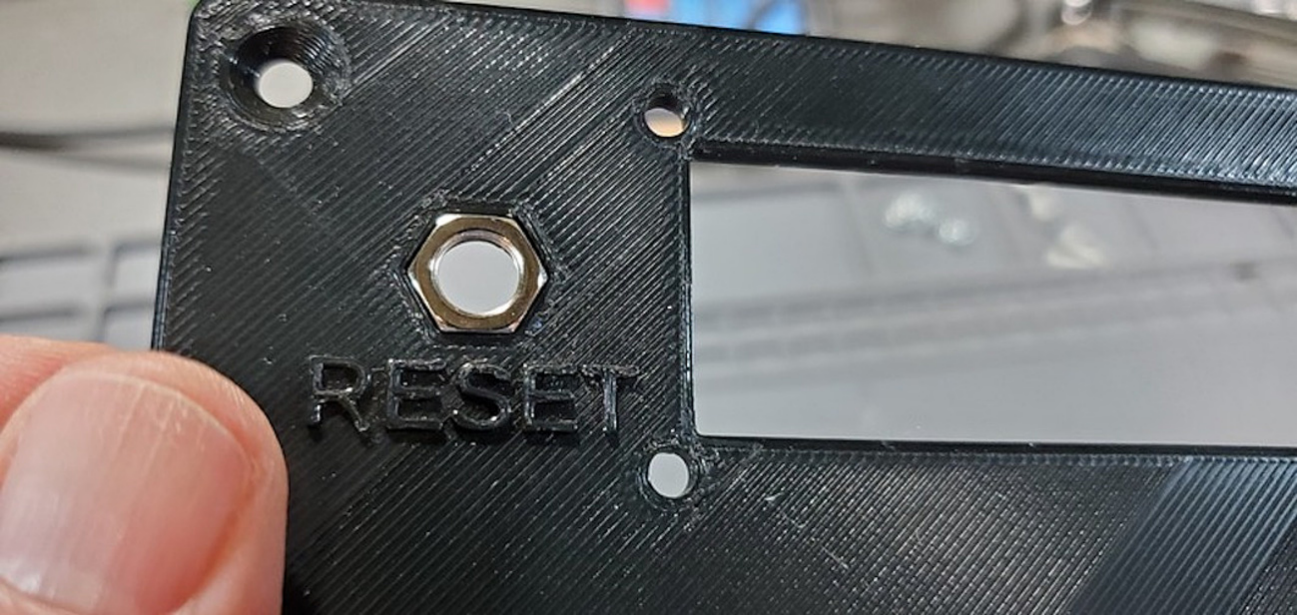
Reset button nut in housing faceplate.

Place the hex nut into the hexagonal pocket in the faceplate above the “RESET” text (Fig. 51).

**Fig. 52:**
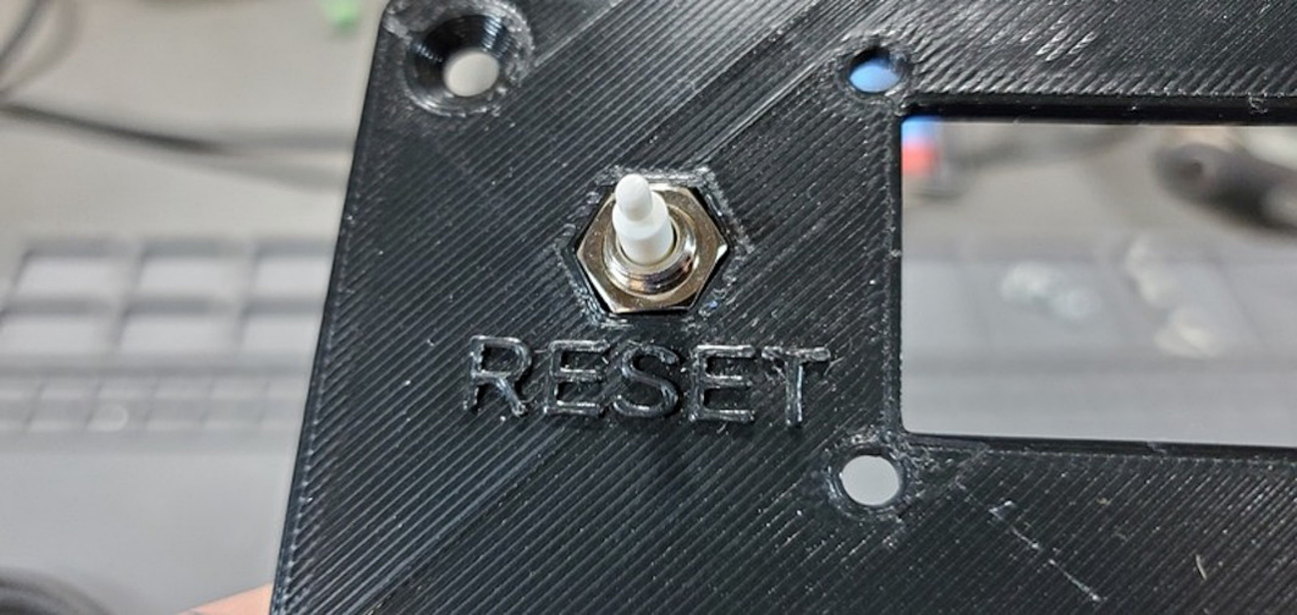
Reset button in housing faceplate.

**Fig. 53:**
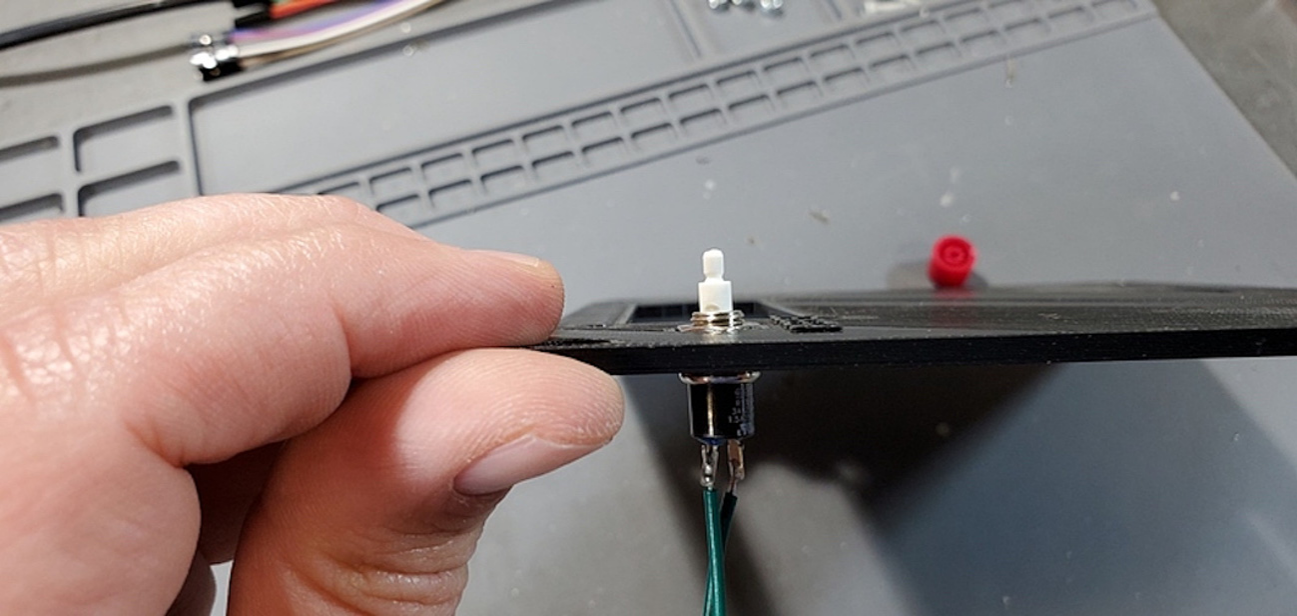
Reset button side view.

Push the reset button through the back face of the faceplate (Fig. 52) and screw it into the nut until the button assembly is tight against the faceplate (Fig. 53).

#### Attach Keypad to Faceplate

**Fig. 54:**
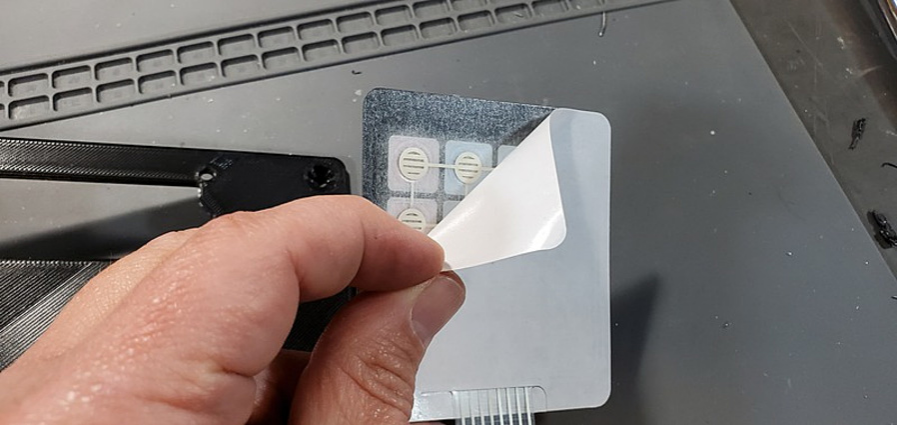
Paper backing on keypad.

Peel the paper backing from the keypad (P6; Fig. 54).

**Fig. 55:**
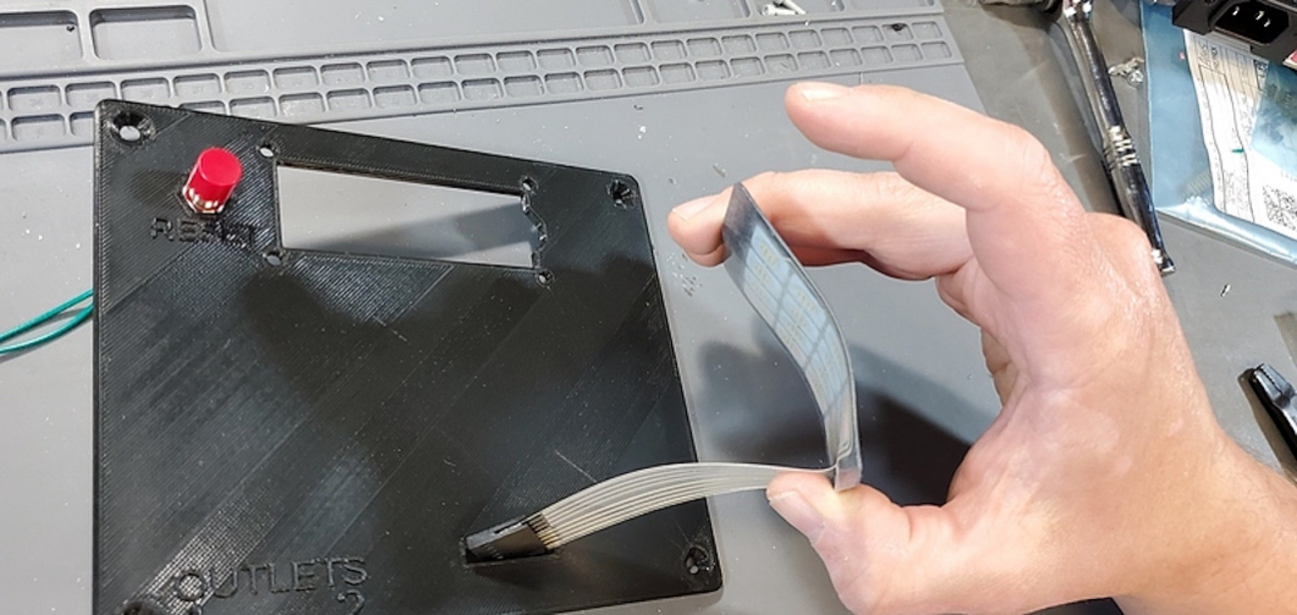
Keypad cable.

Carefully place the female header and flex cable through the slot in the lower portion of the faceplate (Fig. 55).

**Fig. 56:**
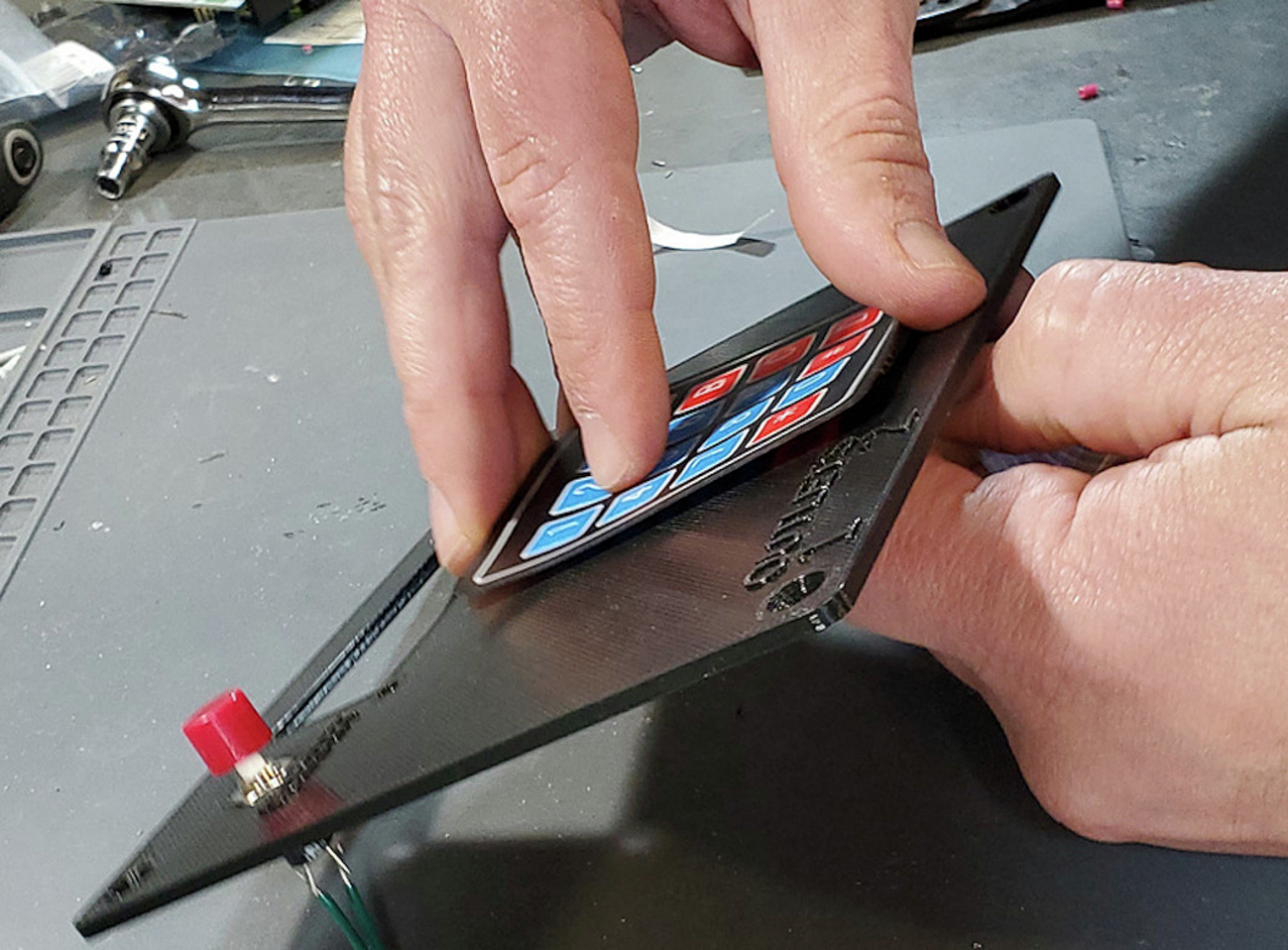
Keypad attached to faceplate.

Pull the flex cable through from the back side of the faceplate to take up the slack in the cable with one hand while placing the keypad square (so the bottom edge is parallel with the bottom edge of the faceplate) with the other hand. Once the keypad is placed down on the faceplate, press down firmly on all parts of the keypad to securely adhere the keypad to the faceplate (Fig. 56).

#### Attach LCD Screen to Faceplate

**Fig. 57:**
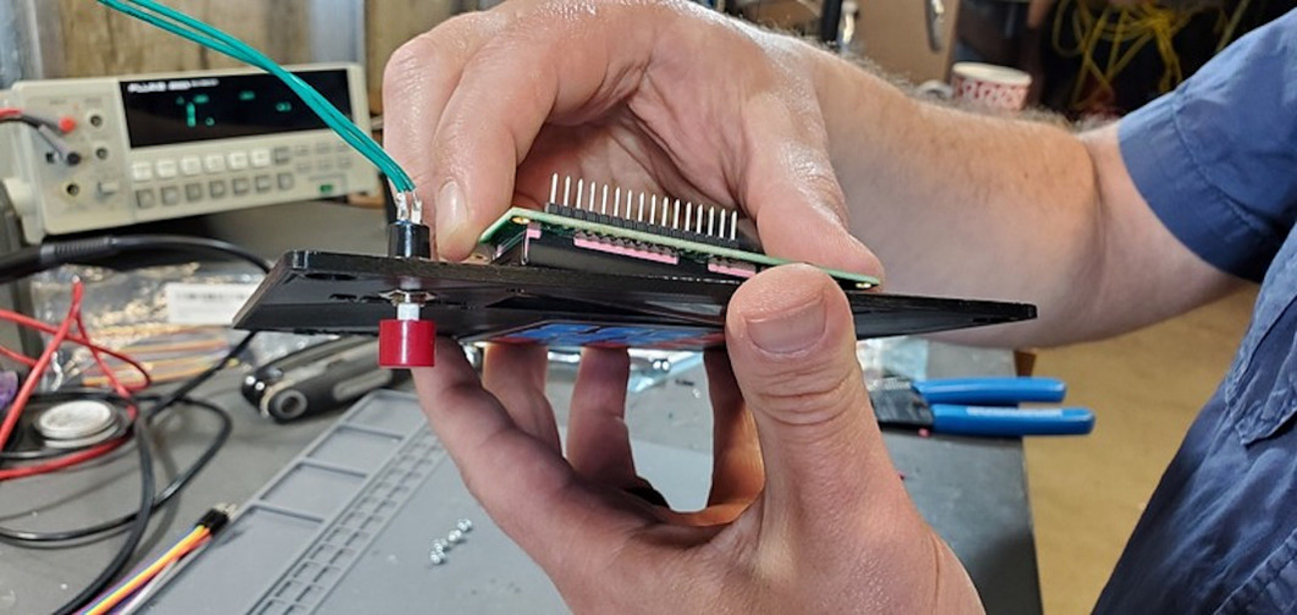
LCD placement in faceplate.

Place the LCD module (P5) into the large opening in the top portion of the faceplate from the backside of the faceplate (Fig. 57).

**Fig. 58:**
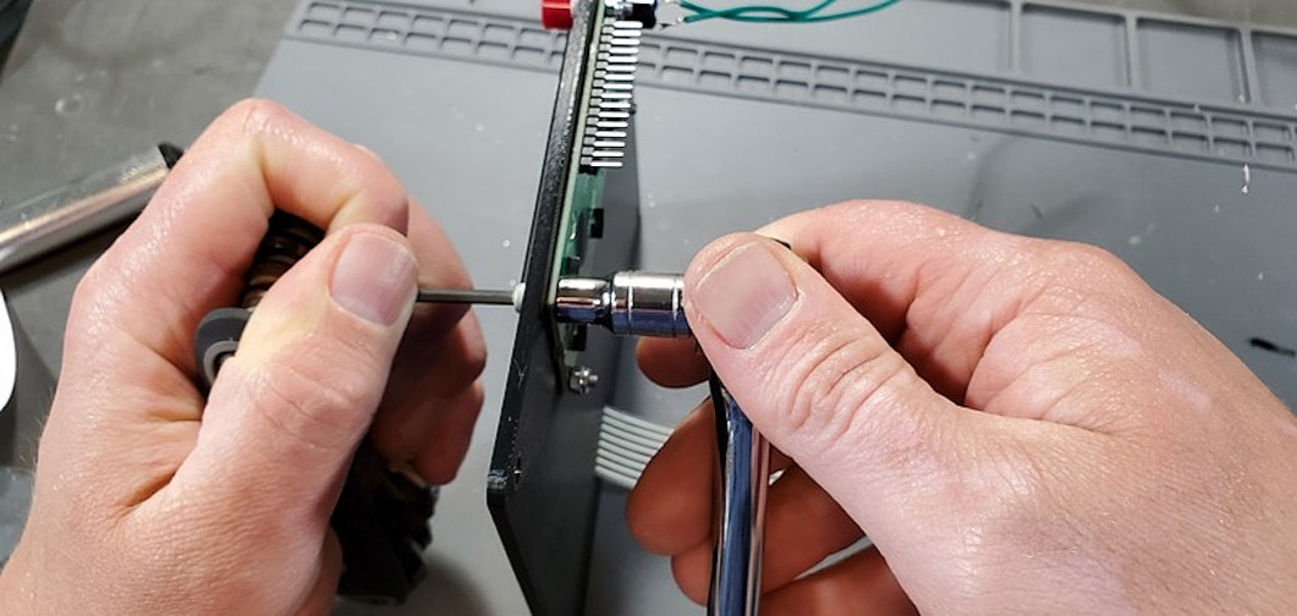
LCD bolts.

Place bolts through each of the four holes in the LCD breakout board and in the matching holes in the faceplate. Tighten nuts with the Allen wrench and socket wrench (Fig. 58).

**Fig. 59:**
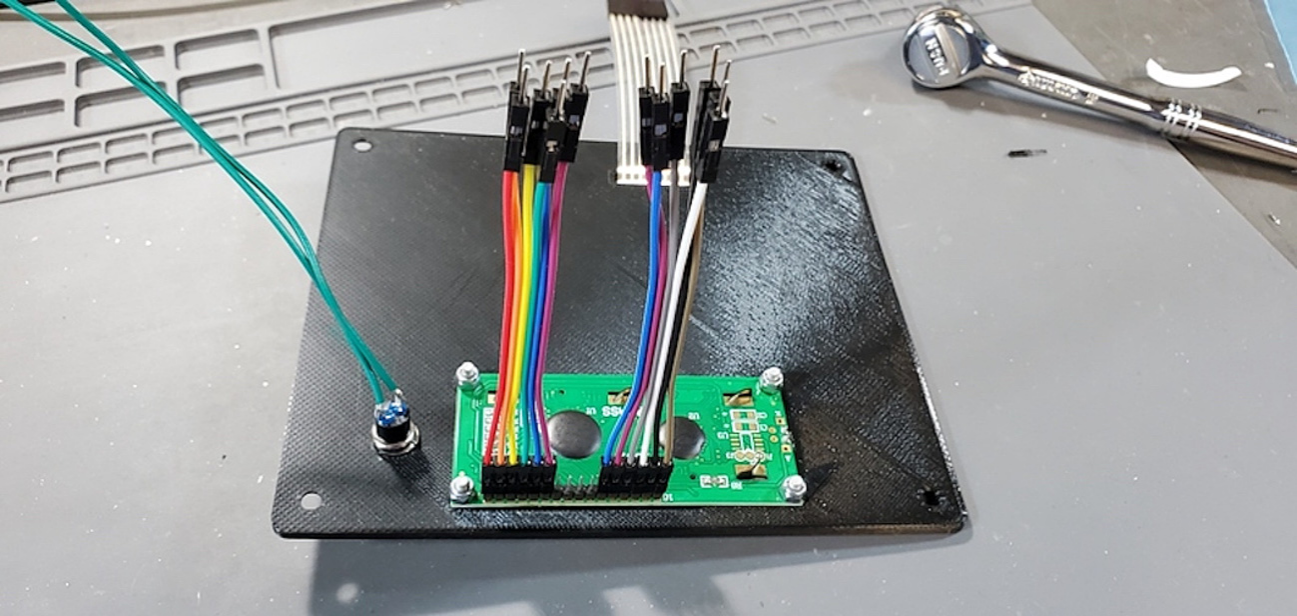
LCD jumpers.

Finally, place female/male jumper cables (P49) on the 6 pins on each end of the header pins on the LCD module (there are 16 pins, but we use only 12; Fig. 59).

### Preparing electrical outlets

#### Parts and Tools

• 14-gauge stranded wire (three colors: white, green and red) (P46)
o one 15 cm section in each color
o one 10 cm section in each color
• 2x panel mount AC outlet (P19)
• Strippers
• Soldering Iron
• Helping hands

**Fig. 60:**
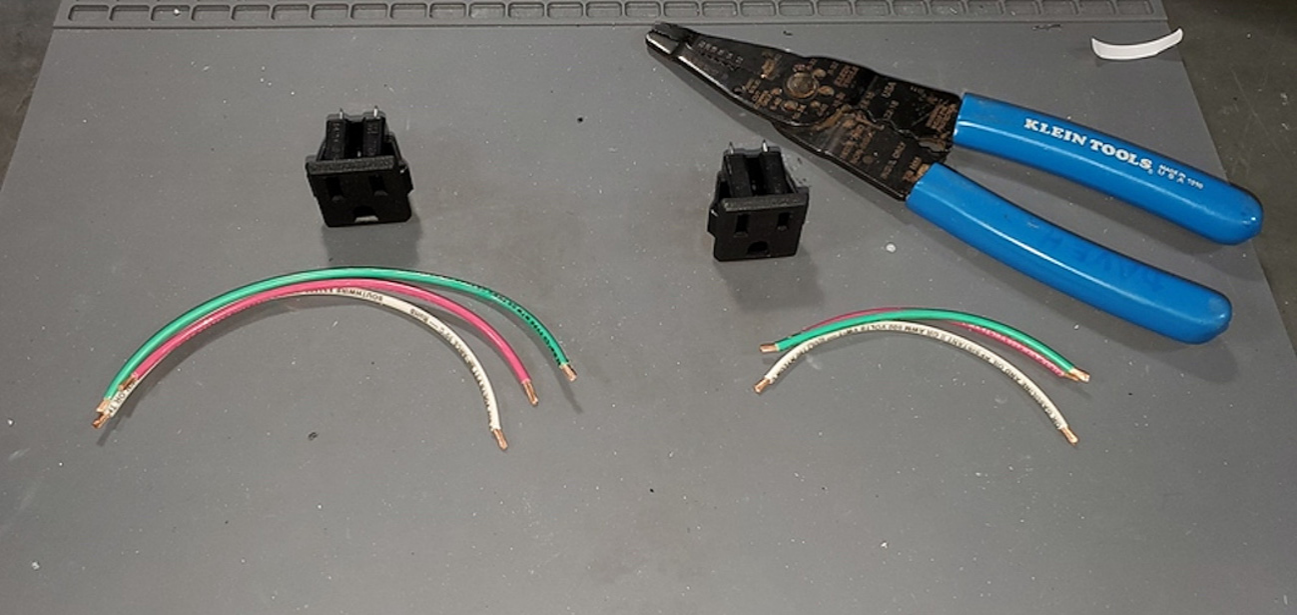
Outlet wires cut and stripped.

Cut the wires and strip 1 cm of insulation from each end of each section of wire (Fig. 60).

**Fig. 61:**
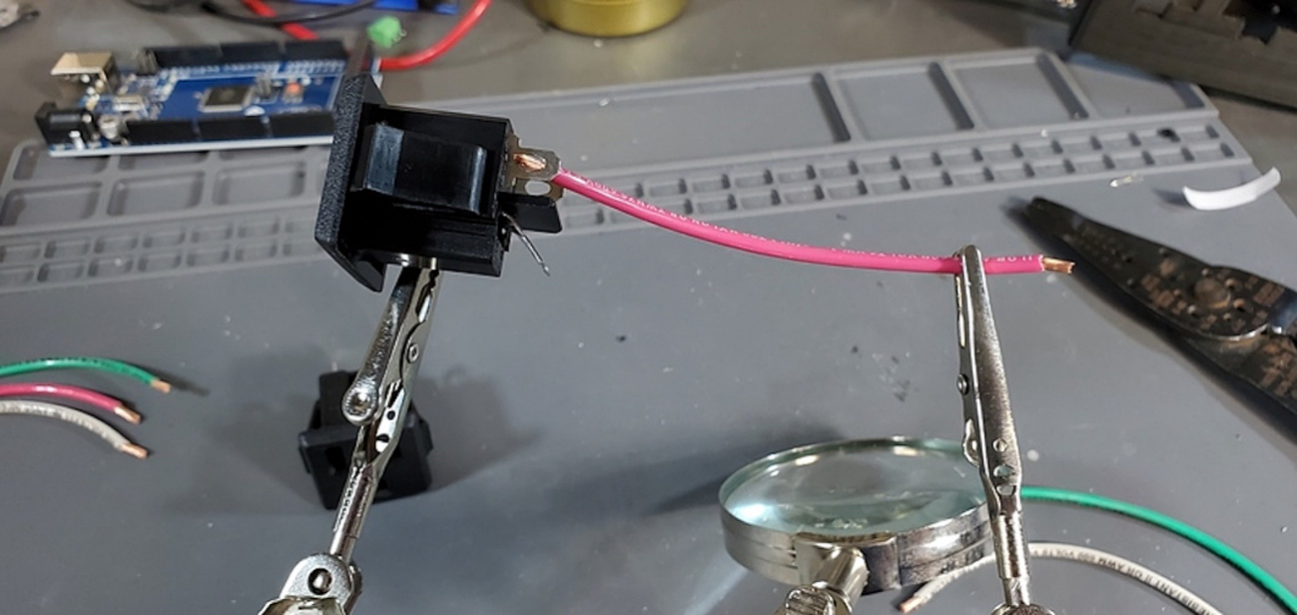
Soldering the outlet wires.

Using the helping hands, solder the 15 cm wires to the tabs on one of the AC outlets (Fig. 61).

**Fig. 62:**
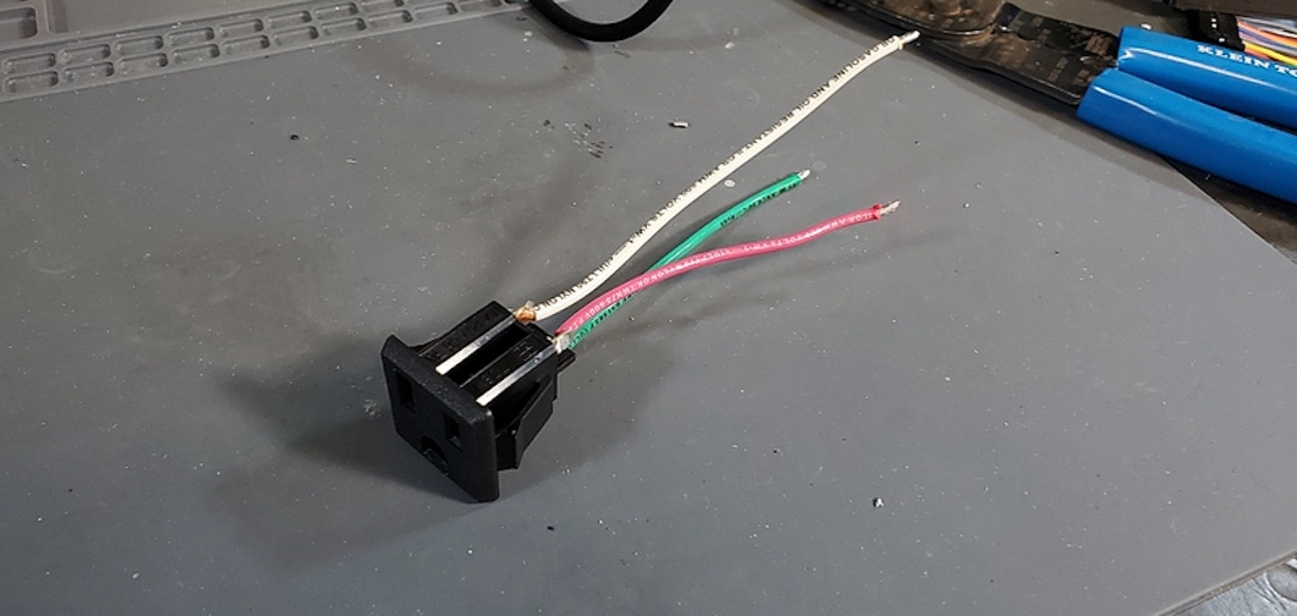
Outlet wires soldered to outlet.

The red wire should be attached to the upper left tab (as you are looking at the tabs straight on), sometimes marked “LM”, the white to the upper right tab, sometimes marked “W”, and the green wire to the lower tab, sometimes marked “G”. Tin the opposite end of the wires with solder (Fig. 62).

**Fig. 63:**
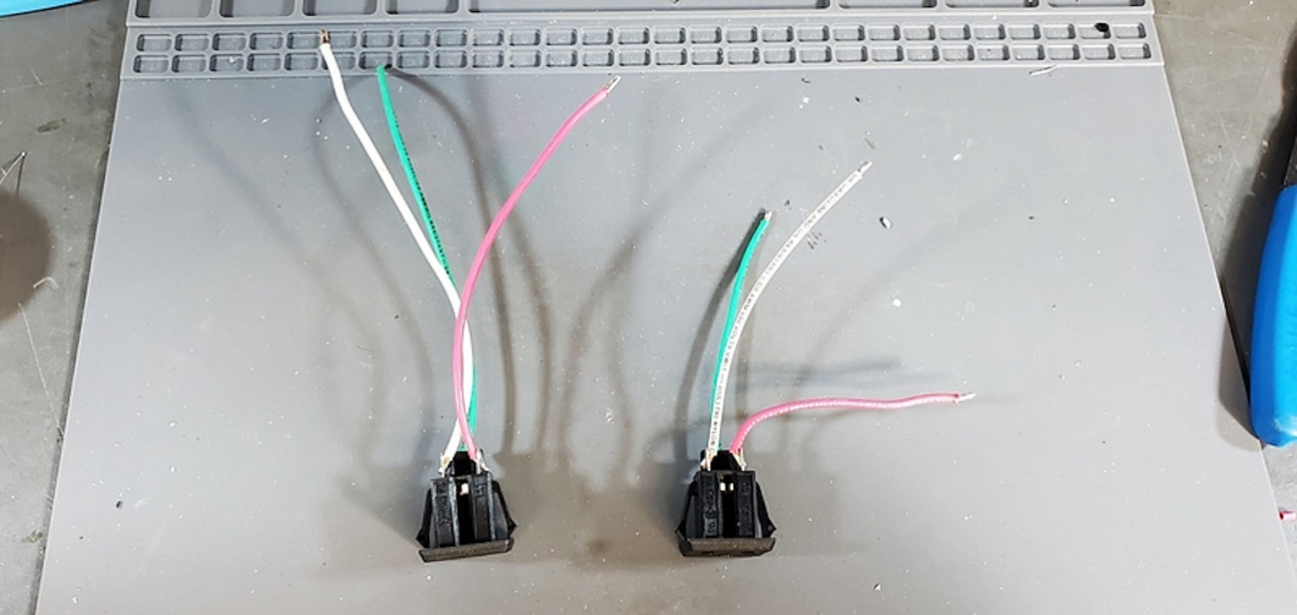
Outlets with wires attached.

Repeat this process with the 10 cm wires and the other AC outlet (Fig. 63).

### Preparing housing skirt

#### Parts and Tools

• 8 × M4 press-fit nuts (P42)
• One M1 bolt (P43)
• 3D printed housing skirt (from section 5.2 above)
• Either 3D printed faceplate or backplate

**Fig. 64:**
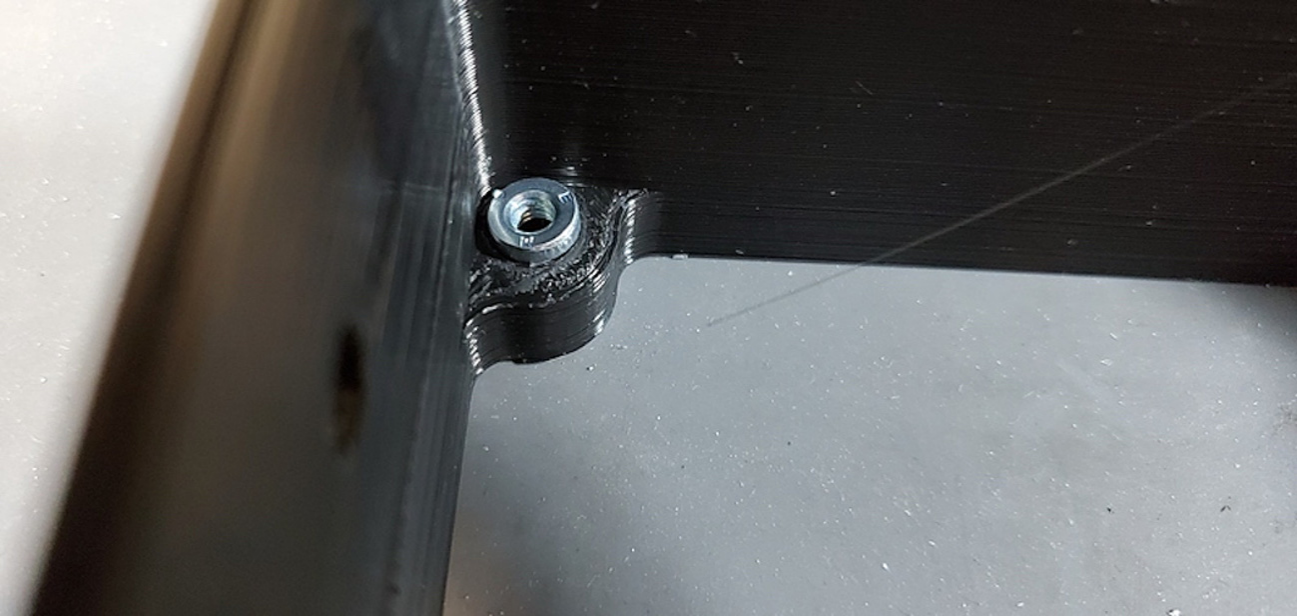
Pushnut placement (not properly sunk).

Place one of the press-fit nuts into the holes in the corners of the housing skirt (Fig. 64).

**Fig. 65:**
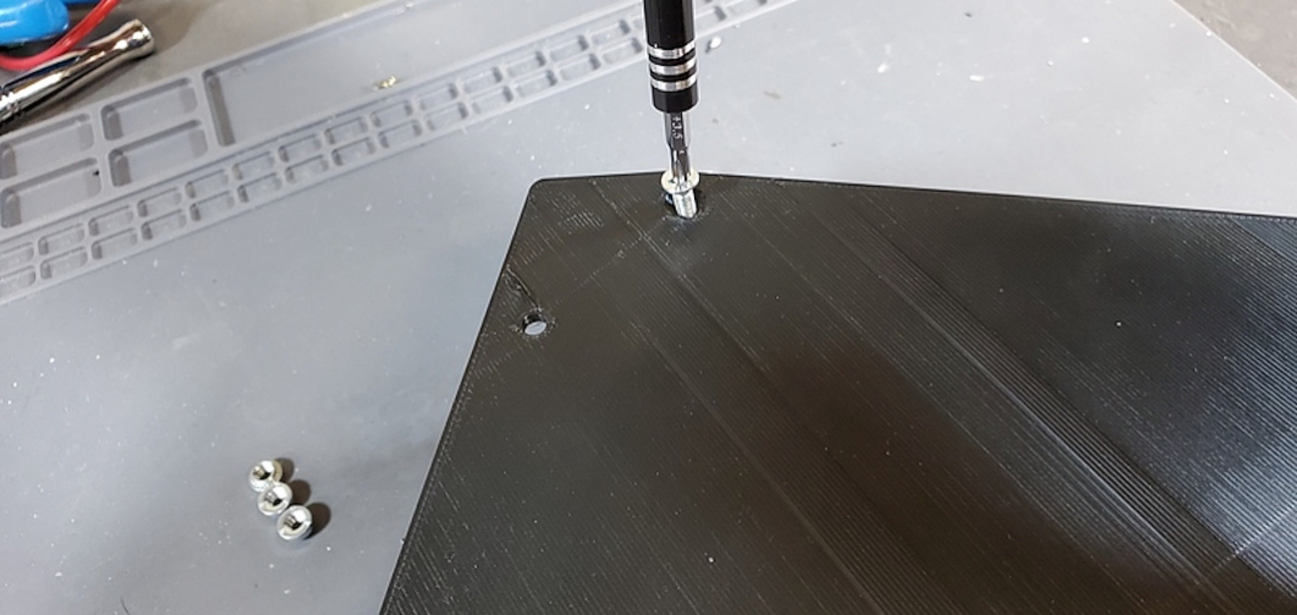
Use bolt to pull nut into place.

If the does not press into place manually (which is likely given the respective sizes), you can use a bolt to pull it into place. To do this hold the nut in place with one hand, turn the skirt over, place the faceplate or backplate on the skirt aligned with the holes, and thread a bolt into the nut (Fig. 65).

**Fig. 66:**
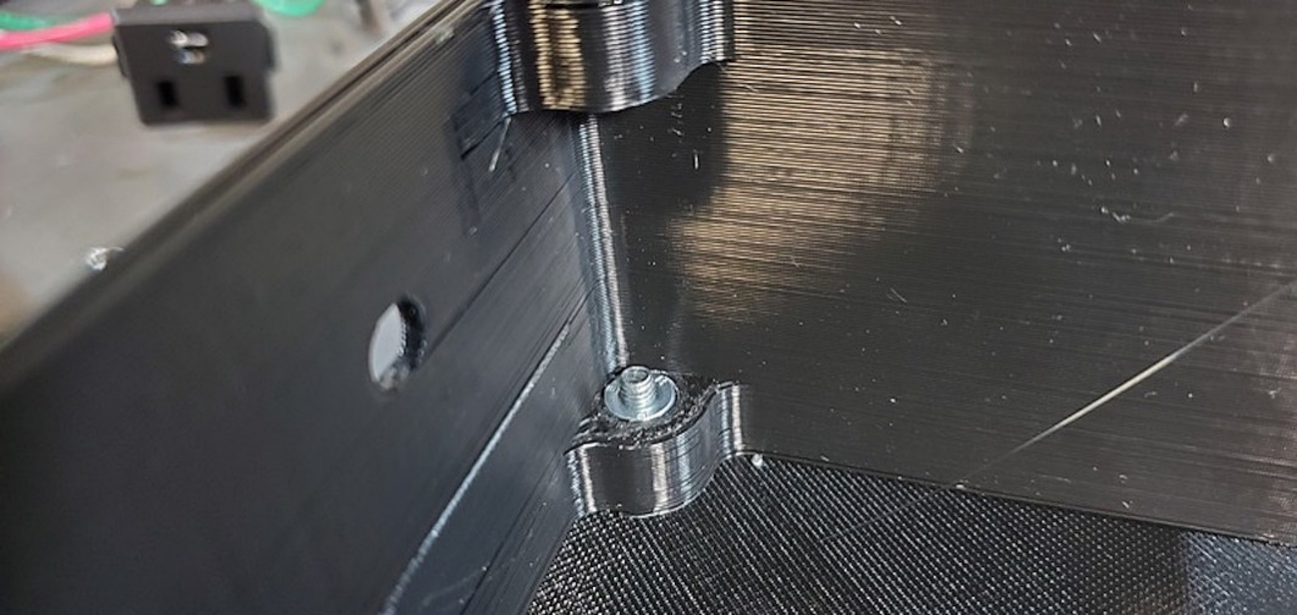
Pushnut sunk to proper depth.

Screw the bolt down to pull the nut into the hole until the exposed face is flush with the plastic (Fig. 66).

Remove the bolt (we will install the faceplate and backplate later) and repeat the process to install the remaining seven nuts.

### Final device assembly

#### Parts and Tools (Fig. 67)

• Prepared housing skirt

**Fig. 67:**
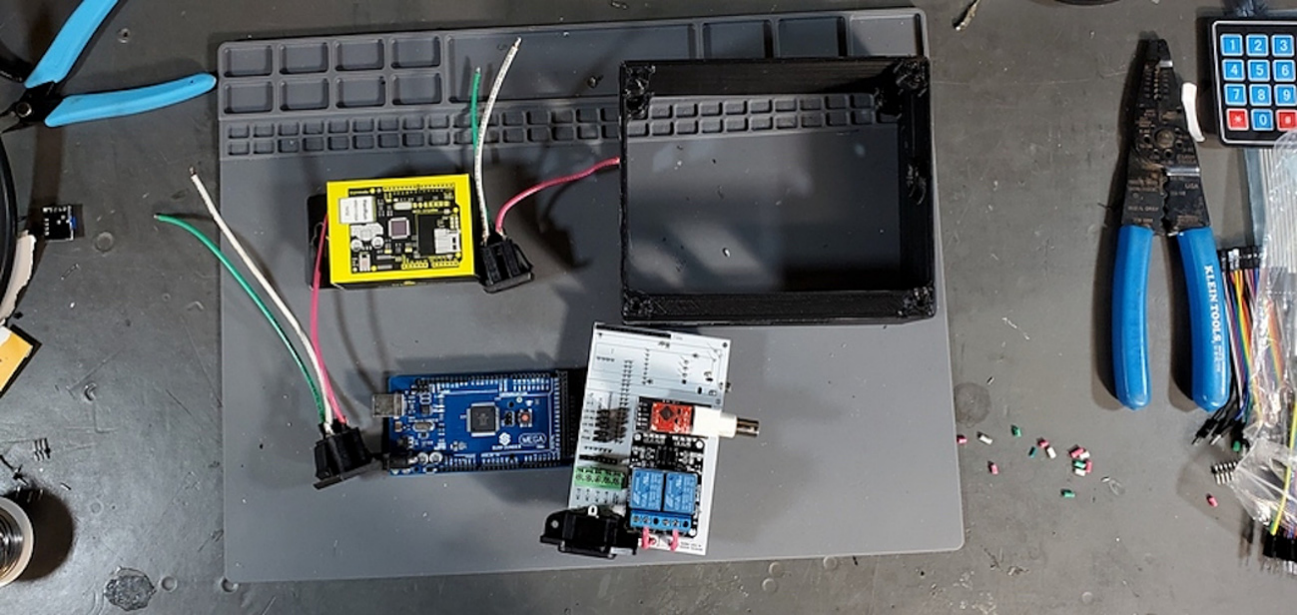
Final assembly parts and tools.
• Prepared electrical receptacles
• Arduino Mega 2560
• Arduino Ethernet Shield v1 (WIZnet W5100)
• Assembled PCB
• Assembled faceplate
• backplate
• 2 × M3 × 0.5 Socket hex head bolts
• 2 × M3 nuts
• 22-gauge wire > 10 cm with 0.5 cm stripped at each end
• 8x flat head M4 bolts
• Metric 2.5 Allen wrench

**Fig. 68:**
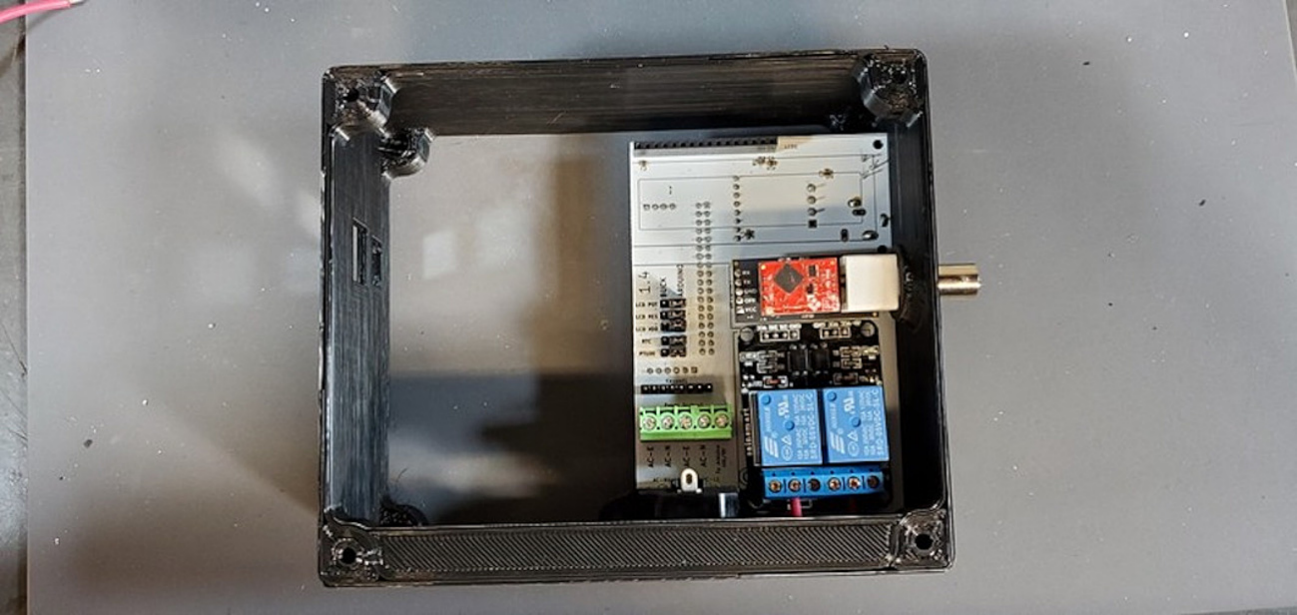
Placement of PCB in housing skirt.

**Fig. 69:**
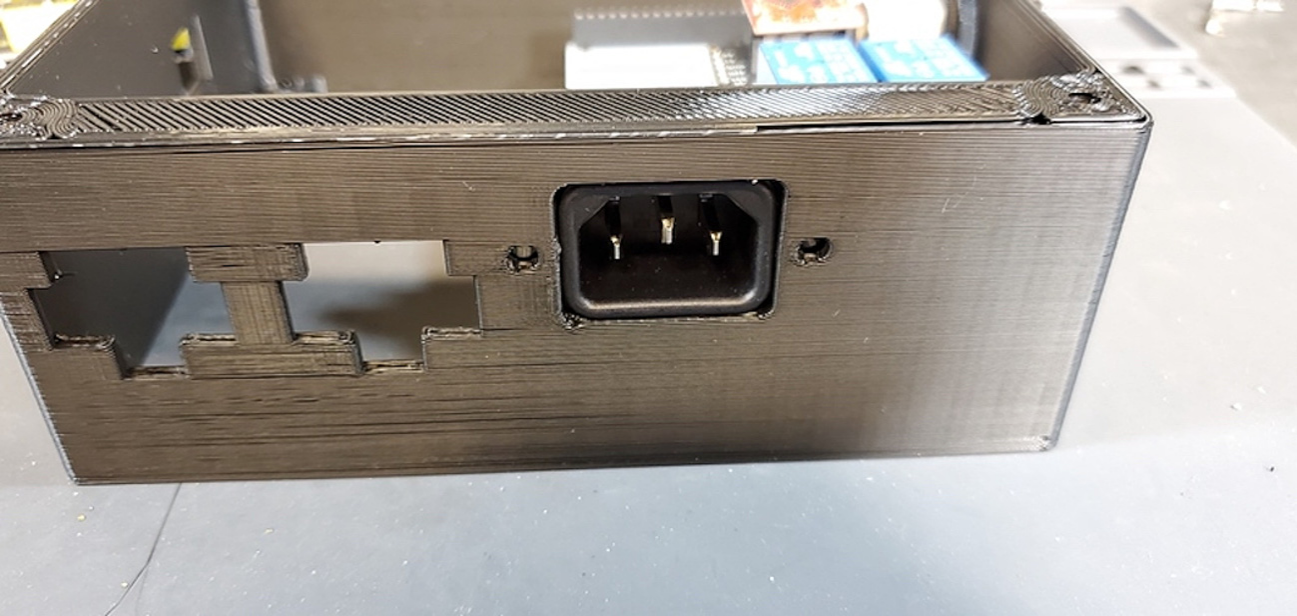
Placement of electrical input in housing skirt.

**Fig. 70:**
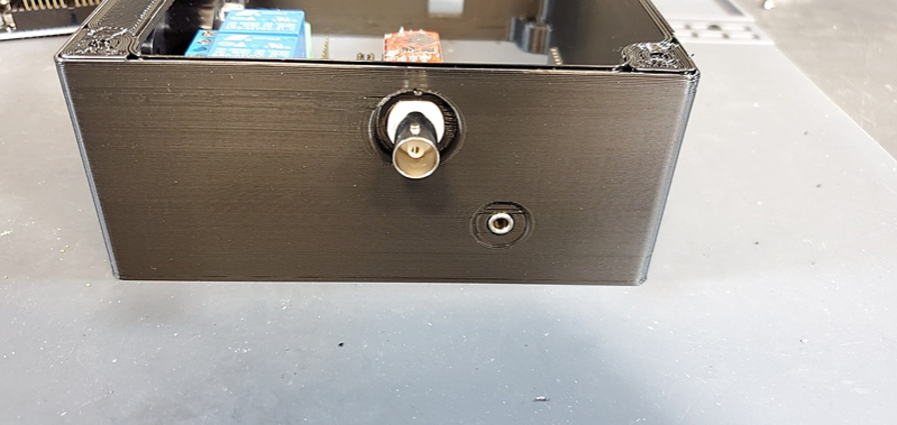
Placement of BNC and 3.5 mm jack in housing skirt.

Place the custom PCB into the housing skirt (Fig. 68), making sure the three-prong AC power receptacle is positioned in the appropriate hole in the skirt (Fig. 69), and that the pH BNC plug and temperature 3.5 mm jack are positioned into their holes as well (Fig. 70).

**Fig. 71:**
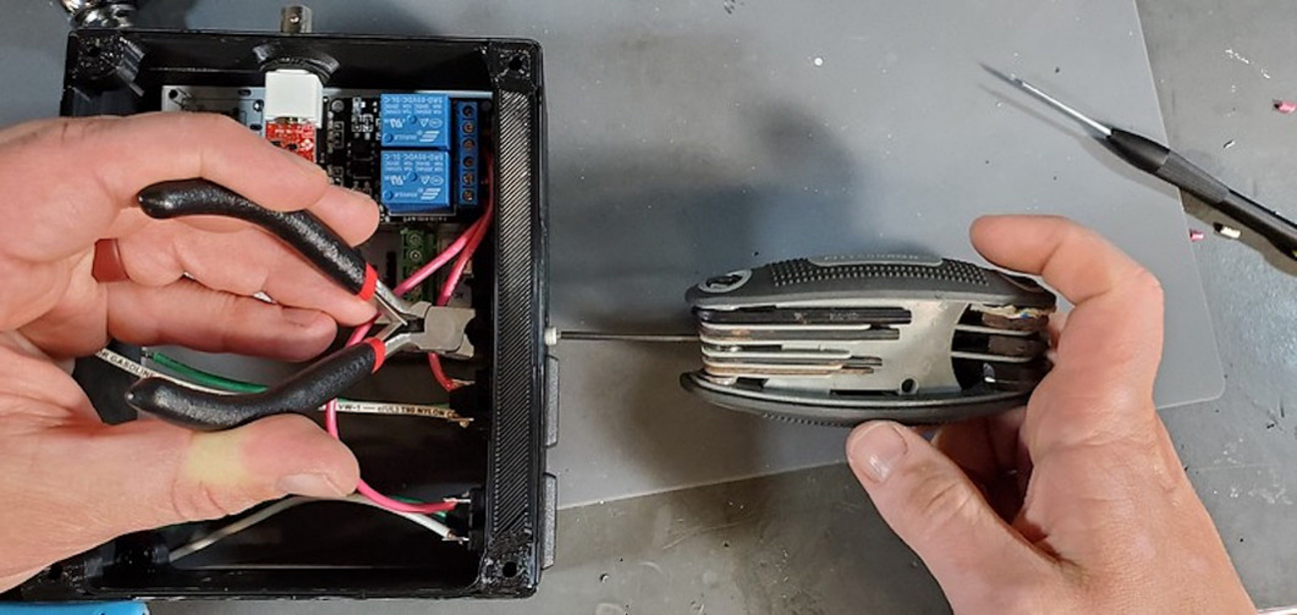
Attach AC power receptacle to housing skirt.

**Fig. 72:**
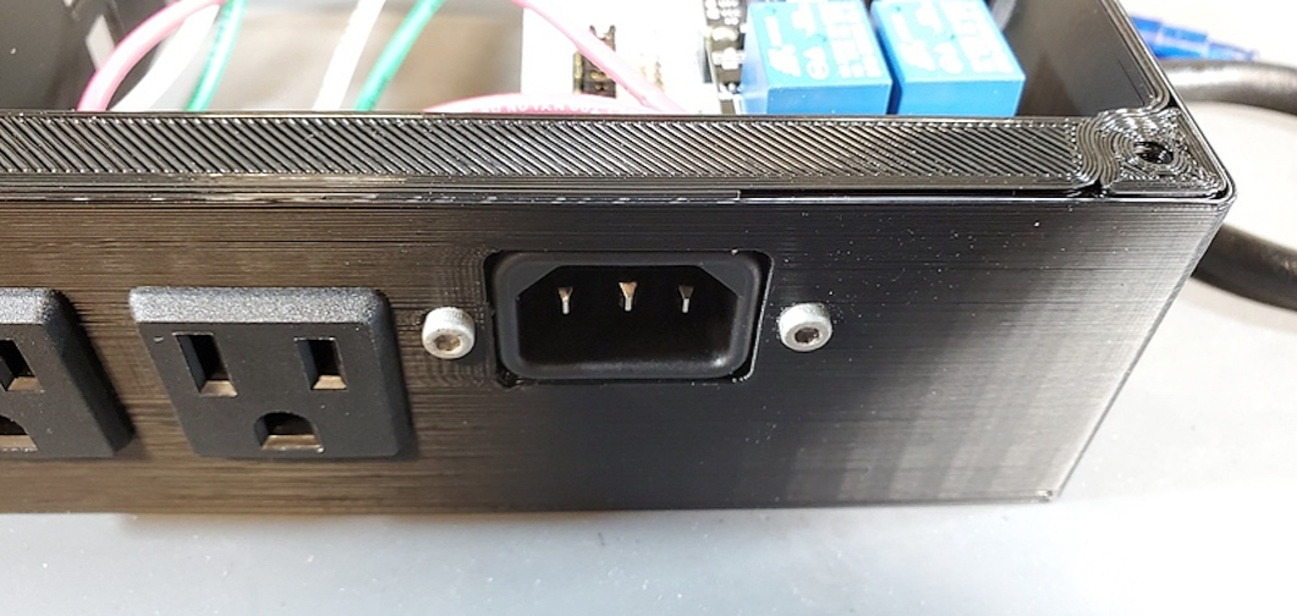
AC power receptacle attached to housing skirt.

Place an M3 × 0.5 Socket hex head bolt through the small holes adjacent to the AC power receptacle. Using pliers (it is difficult to use a socket wrench in this position), place a M3 nut inside the device housing onto the end of the bolt. Using a metric 2.5 Allen wrench, tighten the bolt onto the nut (Fig. 71). The pictures below show these bolts being placed after the AC outlets have been placed and wired, but the task of placing the bolts is much easier before installing the AC outlets (Fig. 72).

Select the AC outlet with the longer (15 cm) wires and push it through the hole (wires first) in the housing skirt farther from the custom PCB (closer to the corner) until it clicks into place. Repeat the process with the other AC outlet.

**Fig. 73:**
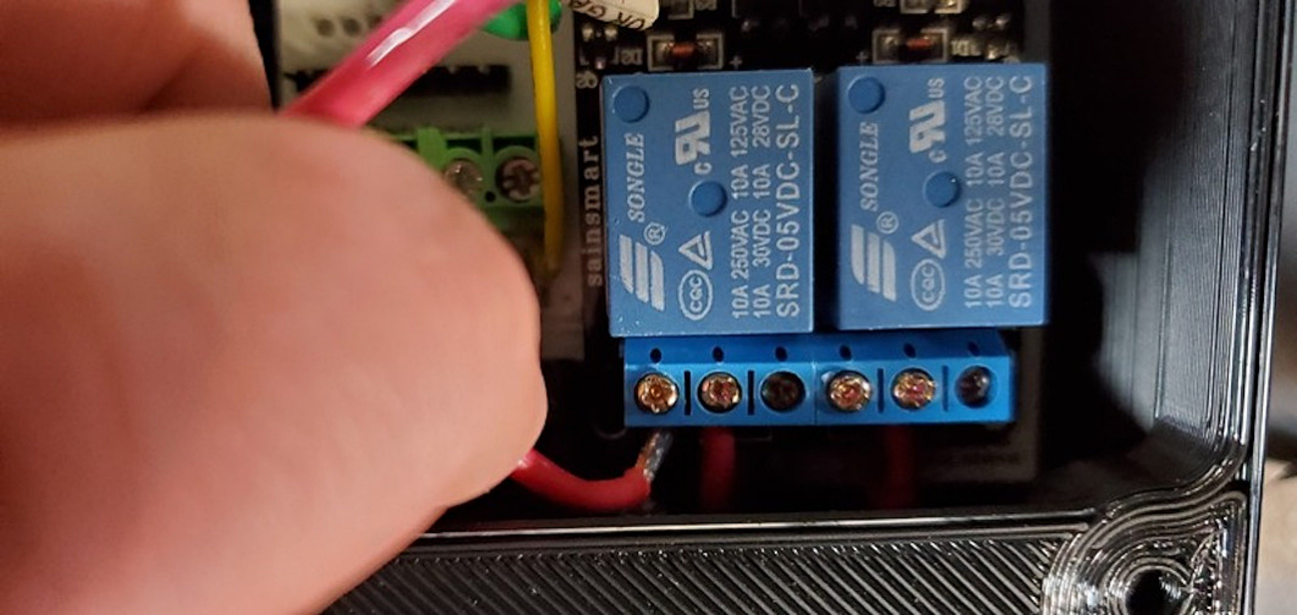
Connecting AC wire to relay.

Each relay (the large blue cubes on the module) has three screw terminals and a short red wire should already be attached from the PCB to the center screw terminal of each relay. Now attach the red wire from the left AC outlet to the left terminal of the left relay. This works best if the tinned end of the wire is bent in a 90-degree angle to the rest of the wire before attempting to connect to the relay module. After the wire has been placed into the correct terminal, tighten the screw on the terminal to secure the wire. Pull gently on the wire to make sure it does not easily come out of the terminal (Fig. 73).

**Fig. 74:**
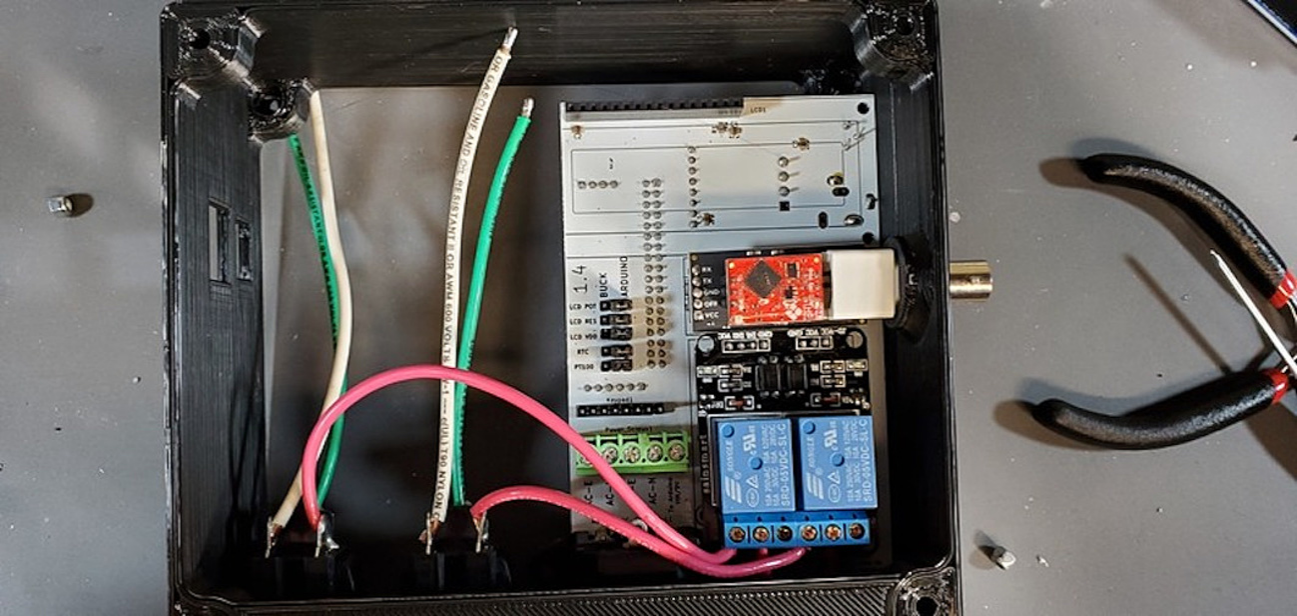
AC wires attached to relay.

Repeat the process with the red wire from the right AC outlet (Fig. 74).

**Fig. 75:**
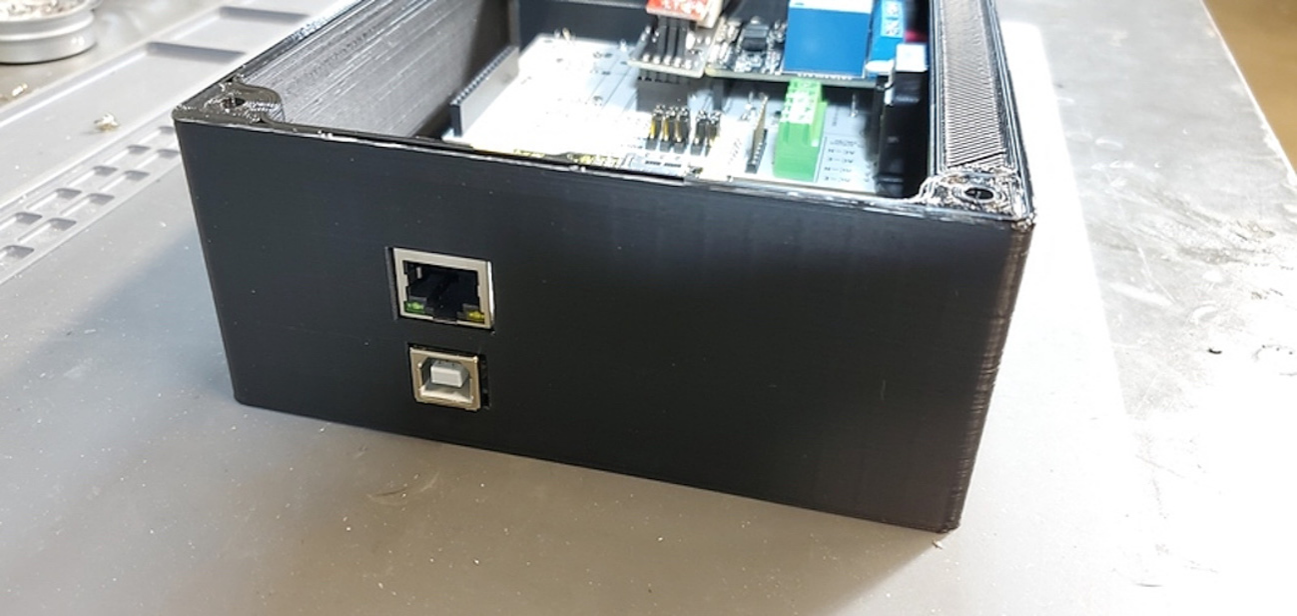
Ethernet and USB outlet placement.

Attach the Ethernet shield (P2) to the Arduino (P1) and turn it over. Turn over the housing so that you are looking at the back of the custom PCB with the buck converters, RTC, and MAX31865 visible. Position the boards at an angle and slide the USB and Ethernet outlets into their respective openings in the housing skirt (Fig. 75).

**Fig. 76:**
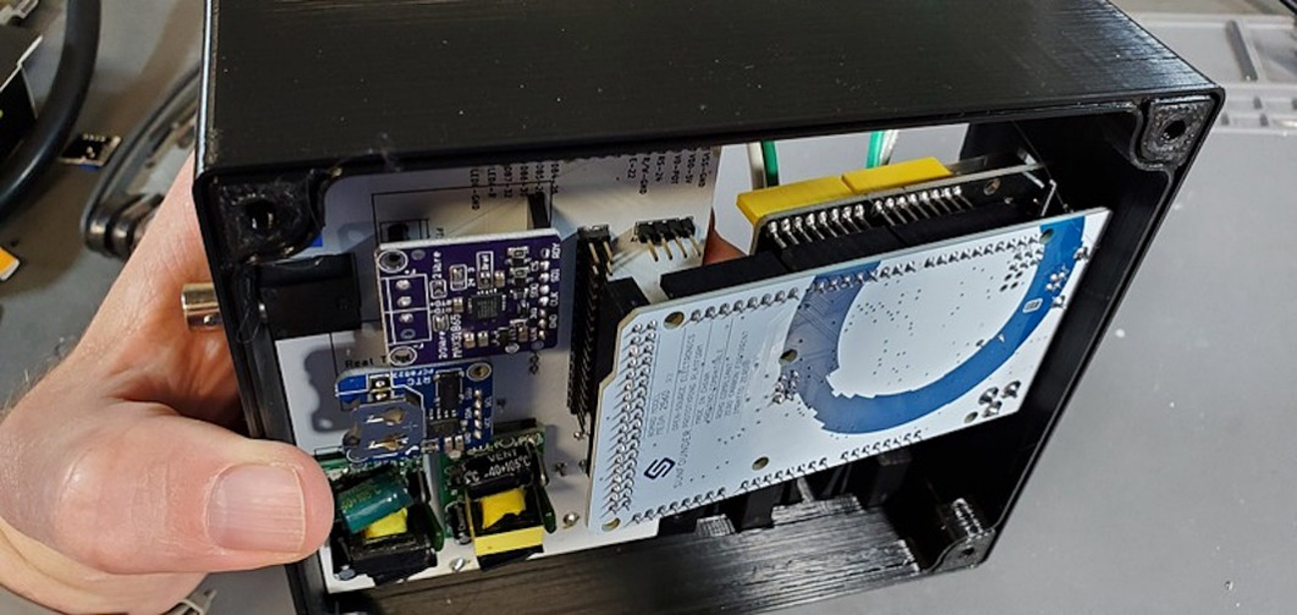
View of Arduino placement from bottom.

**Fig. 77:**
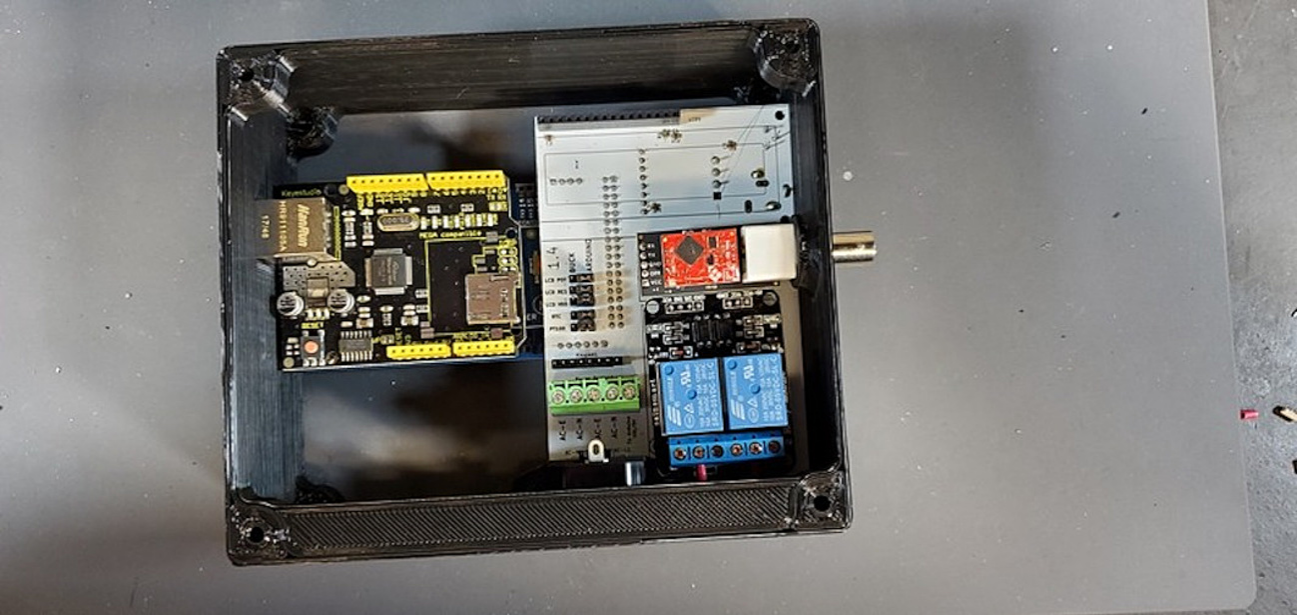
View of Arduino placement from above.

Align the female header pins on the Arduino to match the male header pins on the PCB (Fig. 76) and push them together (Fig. 77).

**Fig. 78:**
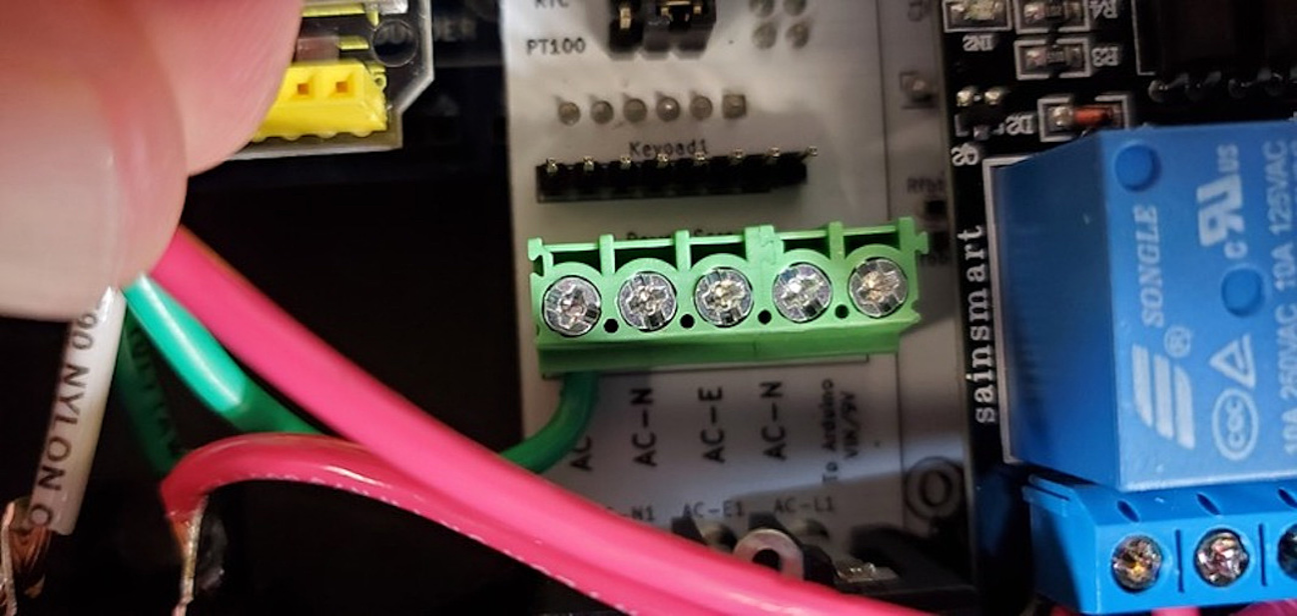
Green wire attached to power terminal screws. (For interpretation of the references to color in this figure legend, the reader is referred to the web version of this article.)

**Fig. 79:**
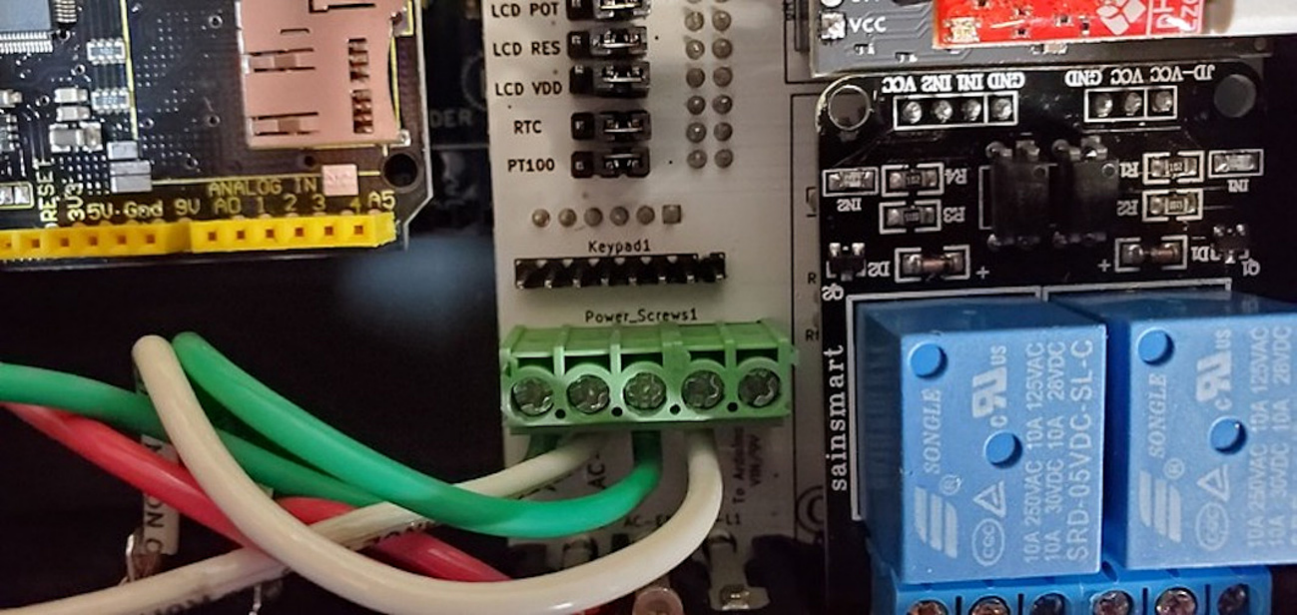
Green and white wires attached to power terminal screws. (For interpretation of the references to color in this figure legend, the reader is referred to the web version of this article.)

Attach the green and white wires of the AC outlet to the large green screw terminal block on the PCB. The green wires should be attached to terminals labeled ‘AC-E’ (Fig. 78) and the white wires should be attached to the terminals labeled ‘AC-N’. Like how the red wires were attached to the 2-relay module screw terminal block, this works best if the tinned ends of the wires are first bent to a 90-degree angle to the wire (Fig. 79).

**Fig. 80:**
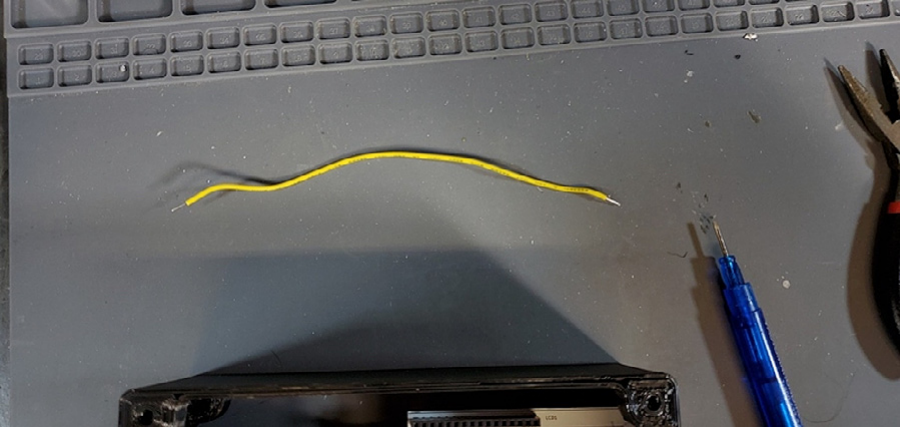
VIN wire for Arduino.

Cut about 10 cm of 22-gauge wire and strip about 0.5 cm of insulation from each end (Fig. 80).

**Fig. 81:**
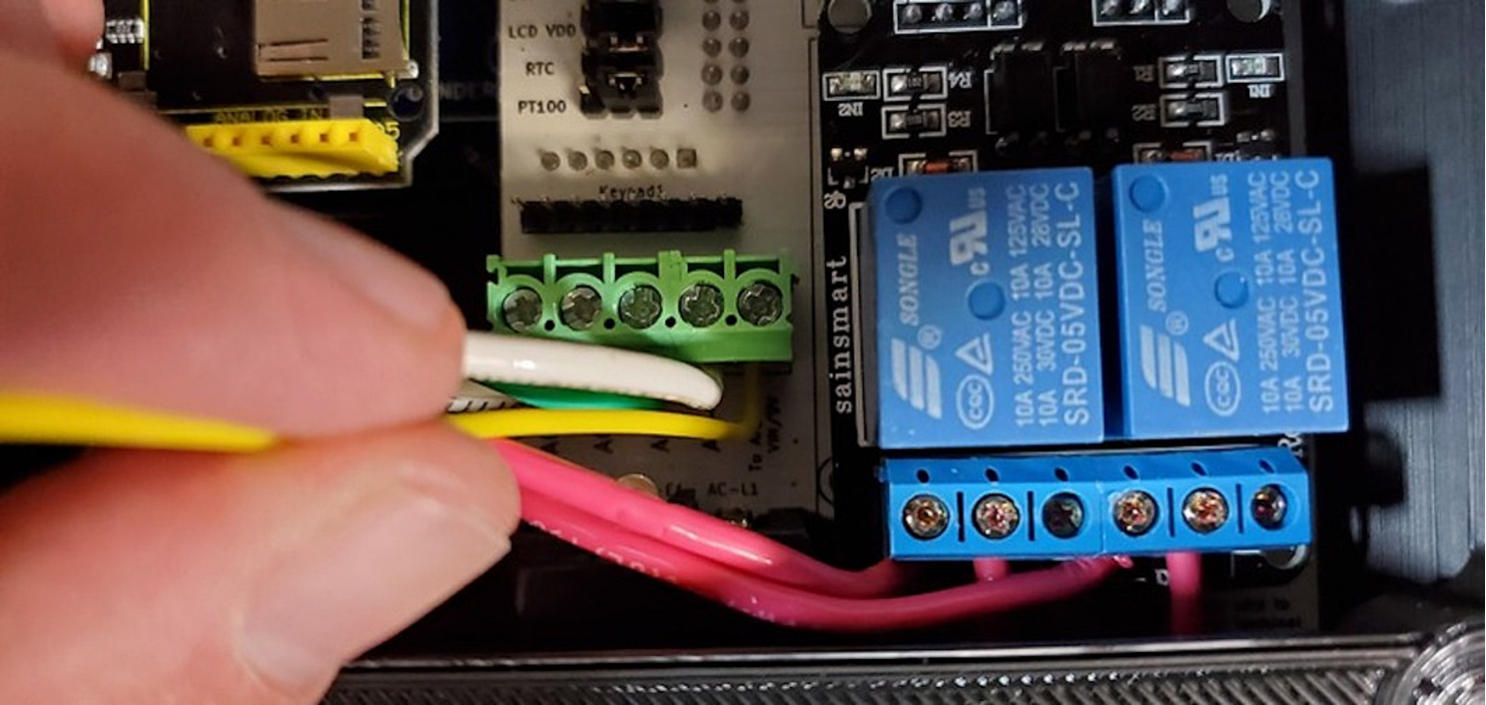
VIN wire attached to green terminal block. (For interpretation of the references to color in this figure legend, the reader is referred to the web version of this article.)

Bend one end about 90 degrees and connect it to the green terminal block in the open position labeled “To Arduino VIN/9V” (Fig. 81).

**Fig. 82:**
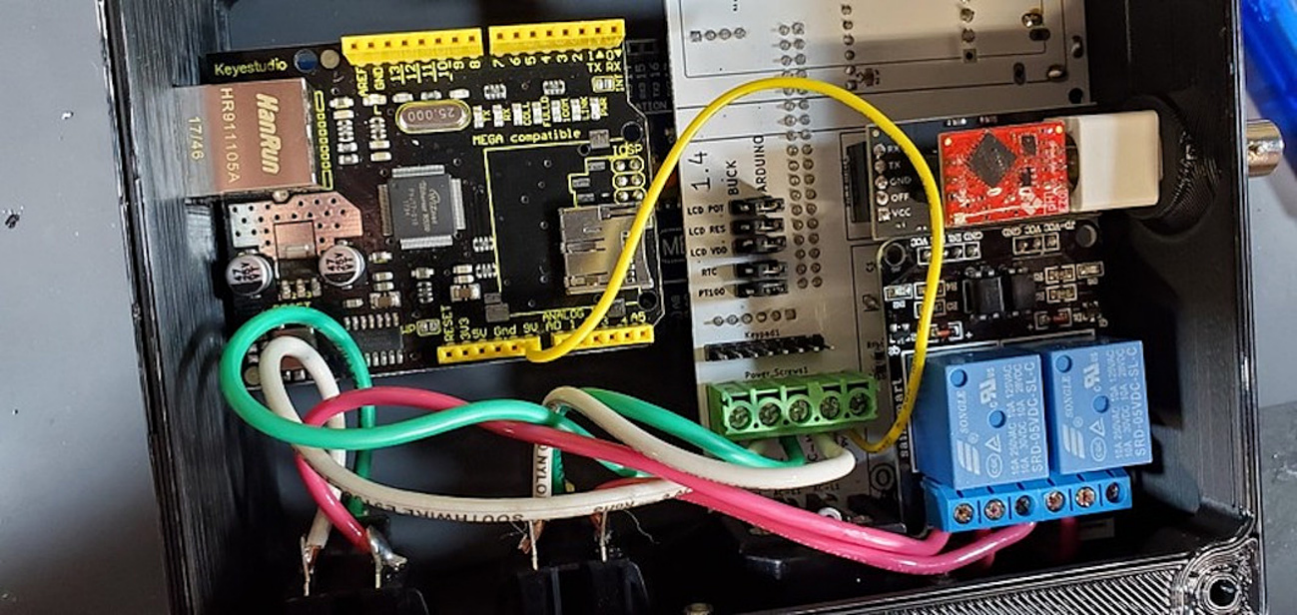
VIN wire attached to the Arduino.

Insert the other end into the Arduino VIN pin on the Ethernet shield. Sometimes this is also labeled “9V”, and is generally between the ground pins, and A0 pin (Fig. 82).

**Fig. 83:**
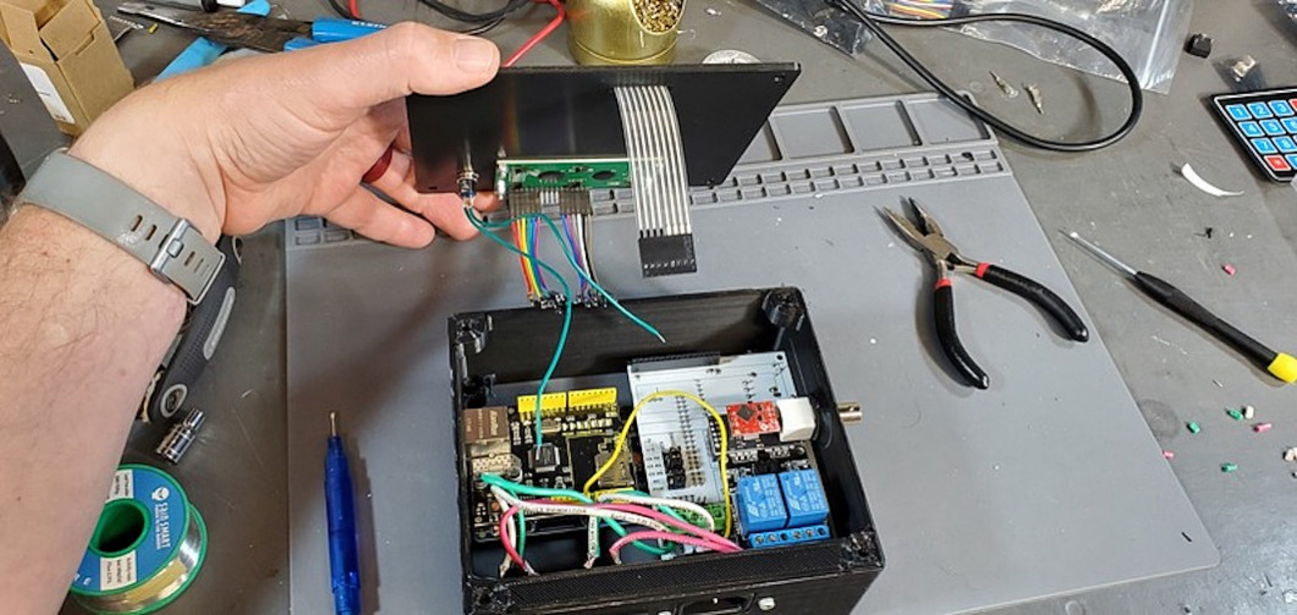
Faceplate positioning over housing skirt.

Position the housing skirt face up with the AC outlets and plug facing you and the BNC connector to the right. Lower the faceplate (top of the housing) toward the housing skirt with the reset button toward the top left (Fig. 83).

**Fig. 84:**
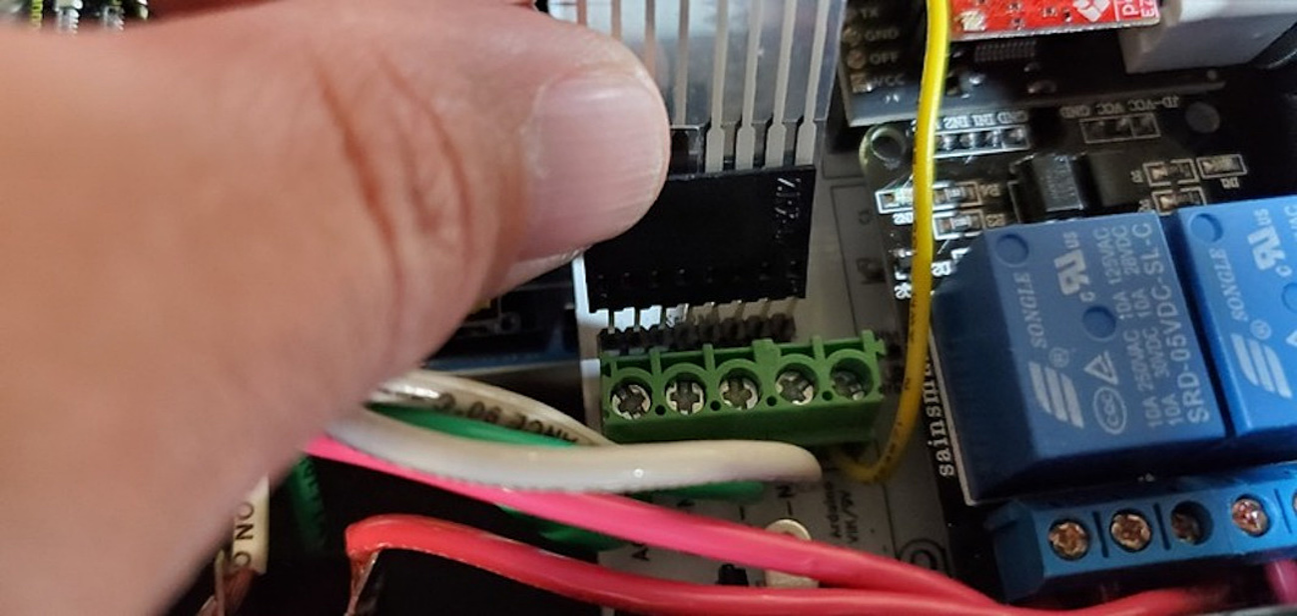
Keypad cable attached to header pins.

Attach the keypad cable from the faceplate to the keypad header pins on the custom PCB (Fig. 84).

**Fig. 85:**
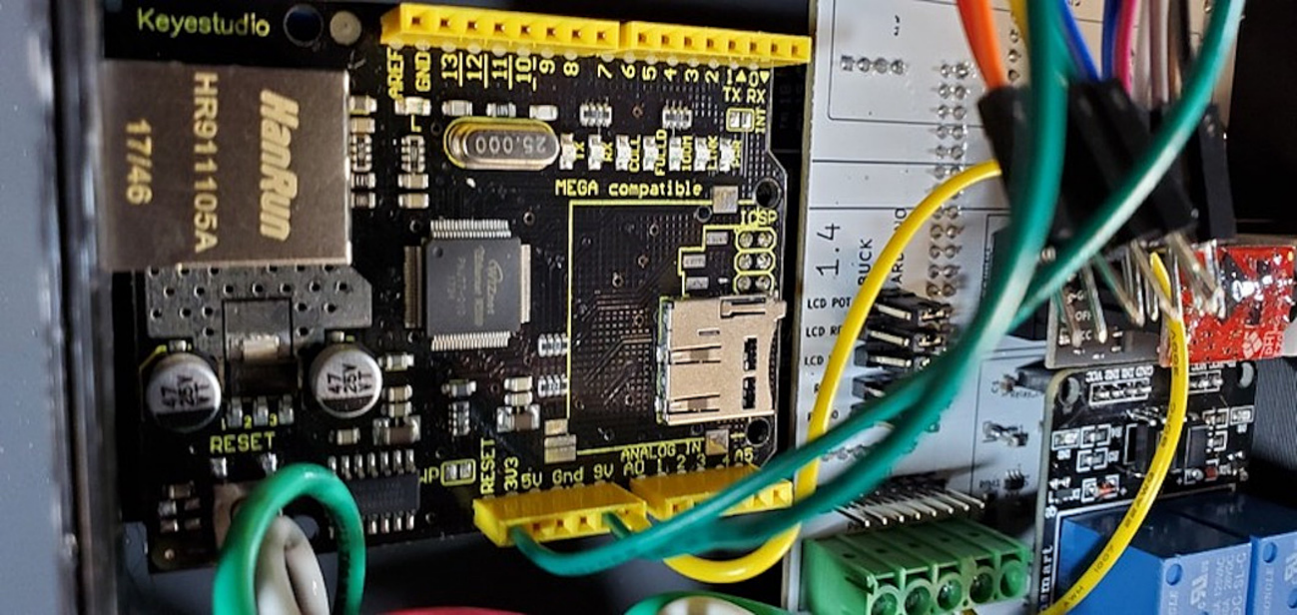
Reset wires attached.

Put one of the wires from the reset button into a ground header on the ethernet shield and the other wire into the reset header on the ethernet shield. Which wire goes into which is not important; they are reversible (Fig. 85).

**Fig. 86:**
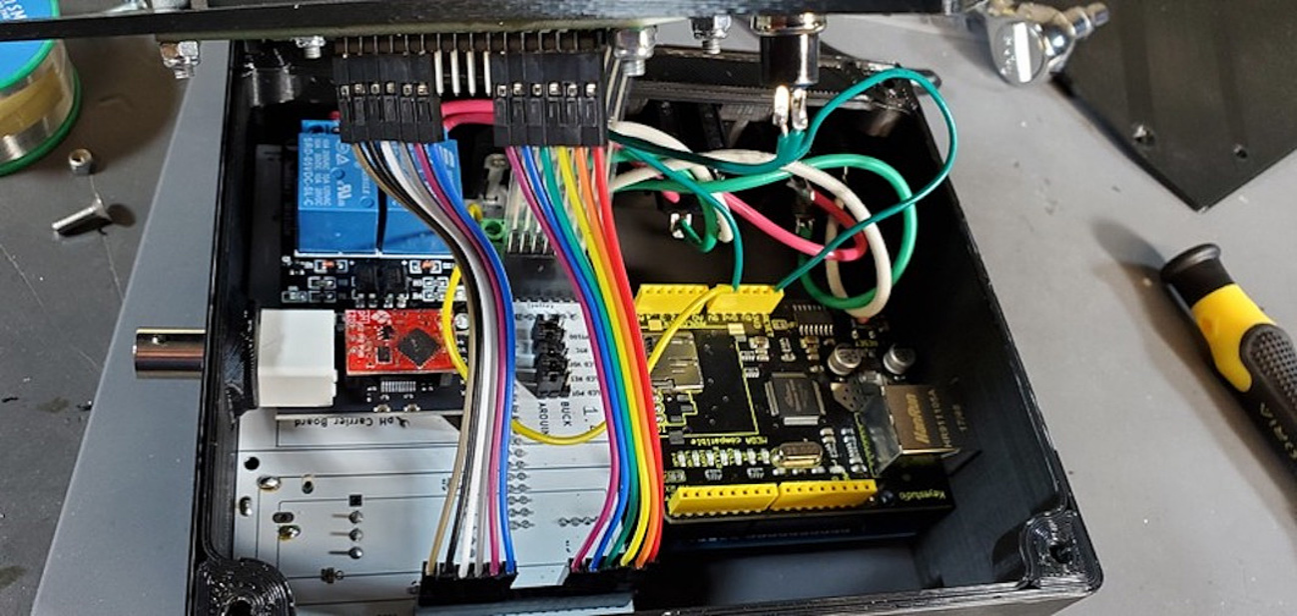
LCD wires attached to custom PCB.

Attach the LCD jumper cables to the LCD header on the PCB. Only the outside 6 LCD pins should have attached wires, and these wires should go in the 6 outside-most headers, in the same order they occur on the LCD (Fig. 86).

**Fig. 87:**
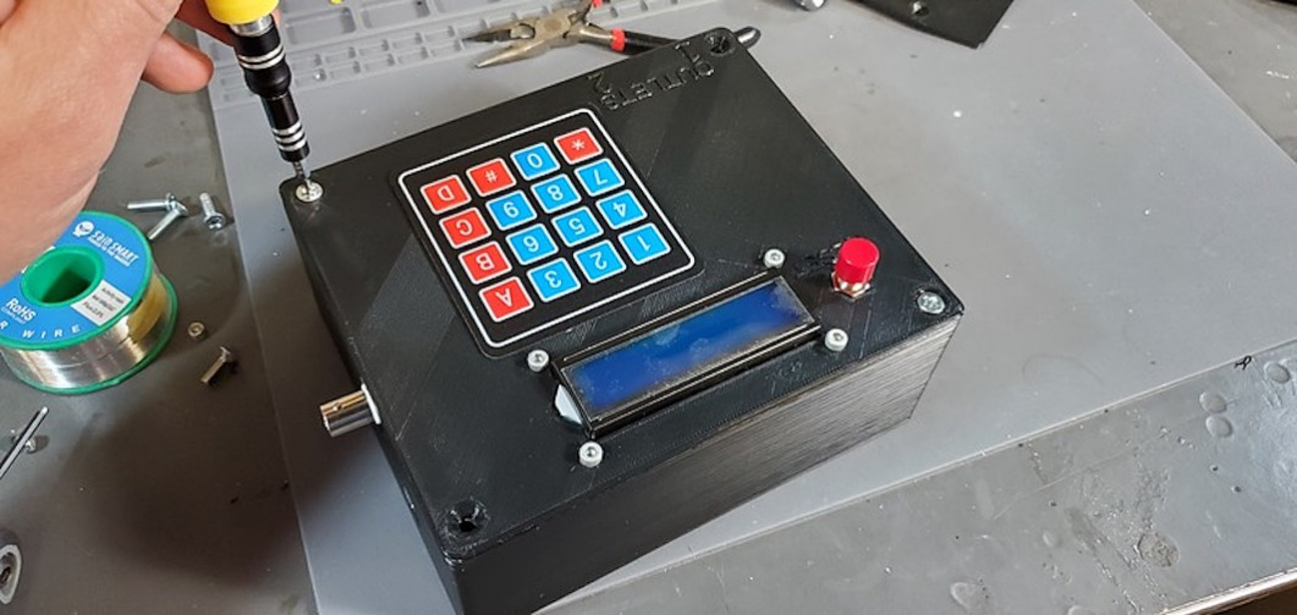
Faceplate attached to housing skirt.

Carefully place the faceplate down on the housing skirt so that no wires are pinched between those two parts and then screw down the faceplate with four M4 flat head bolts (Fig. 87).

After installing software into the device (see section 5.12 below), but before closing the backplate, you will need to adjust the contrast on the LCD screen before you can likely see anything displayed on it.

**Fig. 88:**
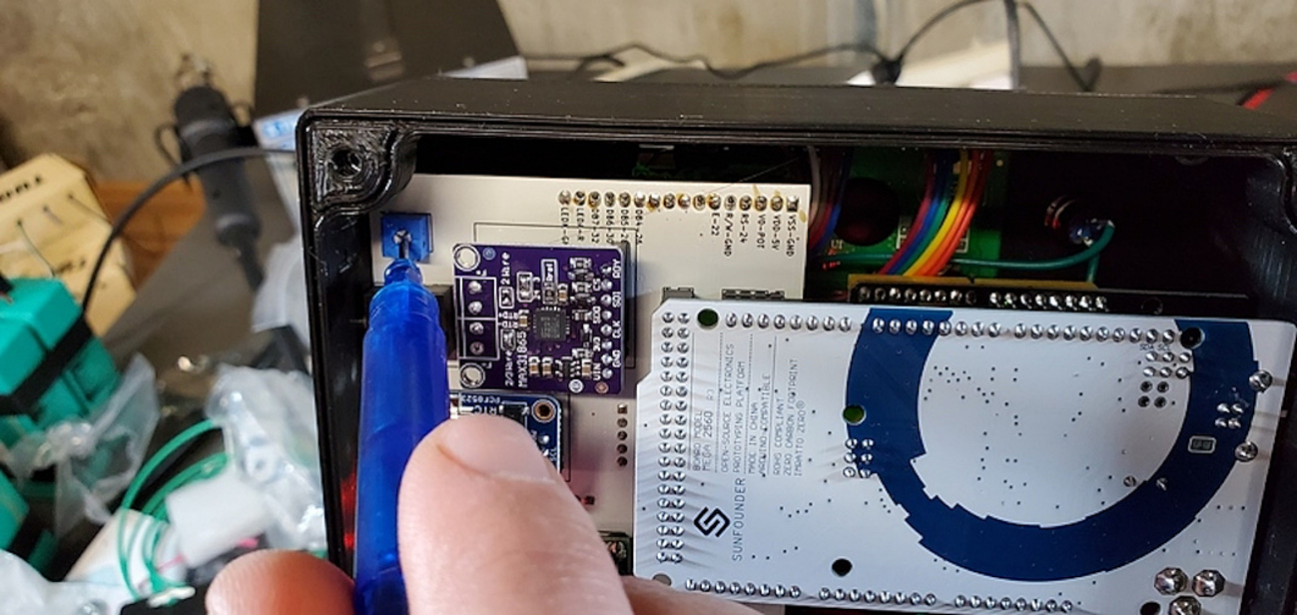
Adjusting LCD contrast.

Use a standard AC power cable to connect the device to a wall outlet. Then, when the device comes on, CAREFULLY, WITHOUT TOUCHING ANY OTHER INTERNAL PART OF THE DEVICE, use the LCD potentiometer mounted on the back of the PCB to adjust the contrast of the LCD screen (Fig. 88).

**Fig. 89:**
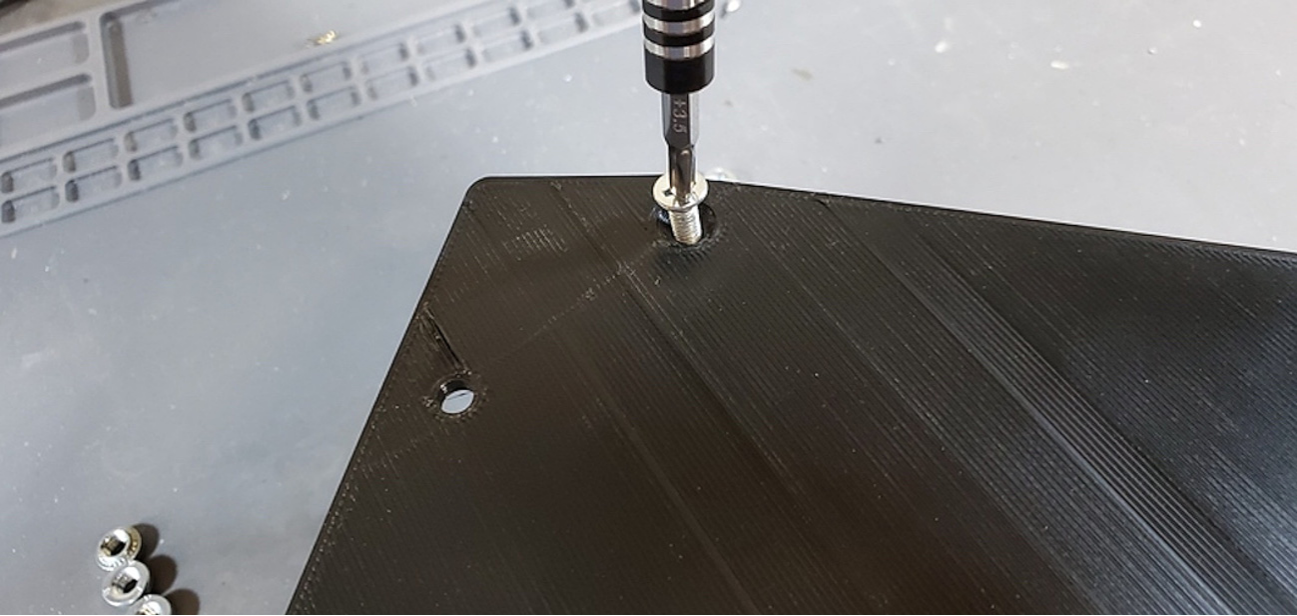
Attaching backplate to housing skirt.

Attach the backplate with 4 M4 bolts (Fig. 89).

**Fig. 90:**
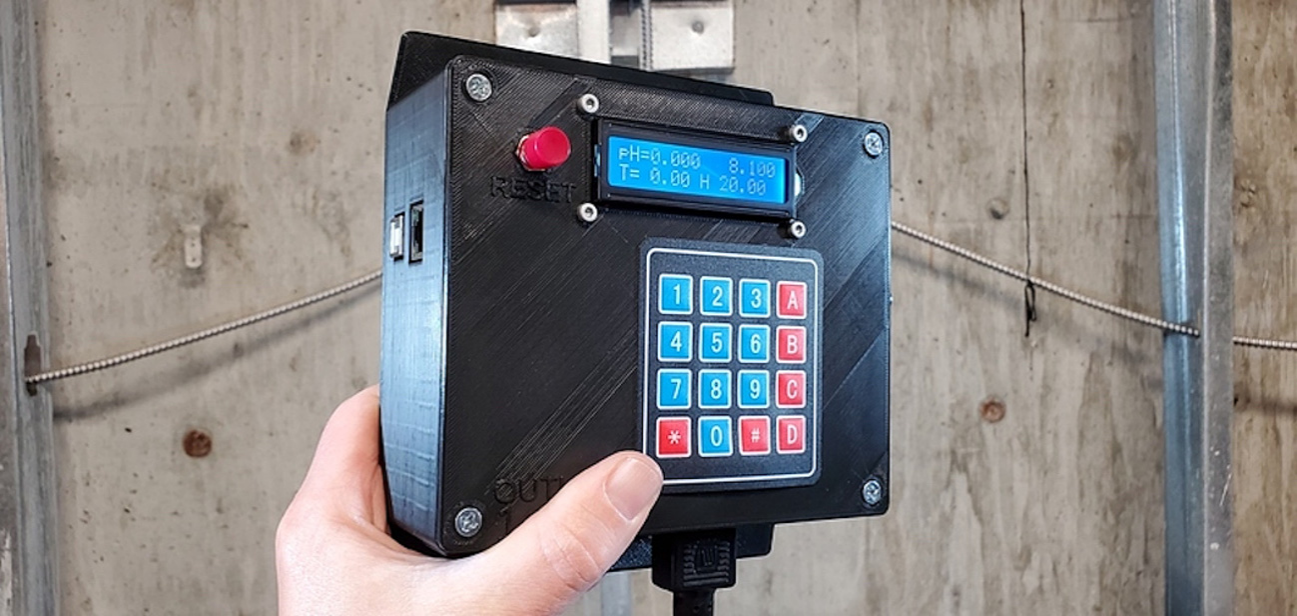
Assembled device.

Device assembly is completed (Fig. 90).

### PT100 temperature sensor preparation

#### Materials Needed:

• 3-Wire PT100 temperature sensor (P4)
• 3.5 mm TRS (P25)
• Epoxy
• Wire Strippers
• Wire Cutters

**Fig. 91:**
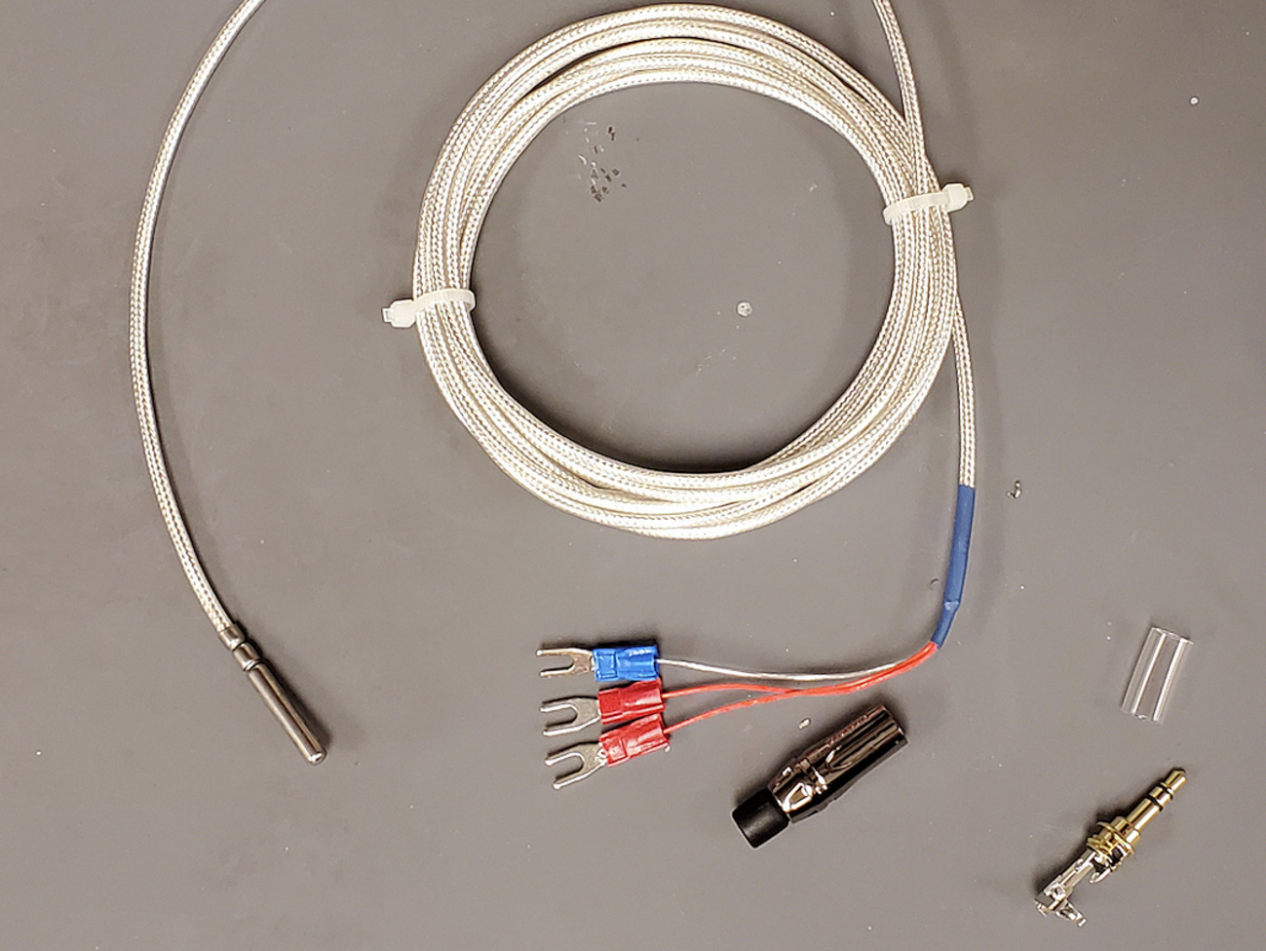
Temperature probe and parts.

The 3-wire PT100 temperature sensor (P4) comes with three stud spade terminal connectors crimped onto the three wires. We will replace the three connectors with one 3.5 mm TRS jack (Fig. 91).

**Fig. 92:**
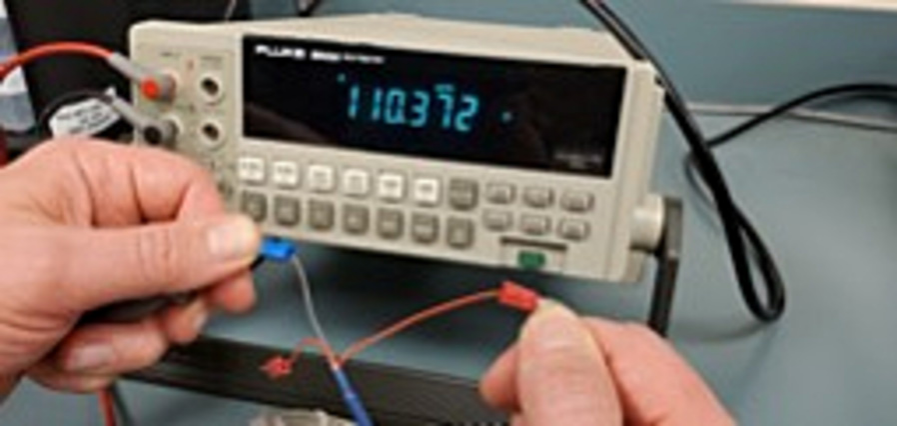
Identify wires on sensor.

The temperature sensor has two wires that are shorted together at the end of the probe, and those two wires are connected to the third through a platinum thread with relatively high resistance (about 100 O at 0C). First determine which wires are shorted together using a multimeter. They are typically given the same color and will have around 1 O of resistance between them. Also make sure that the third wire shows approximately 100 O of resistance to the other two wires (Fig. 92).

**Fig. 93:**
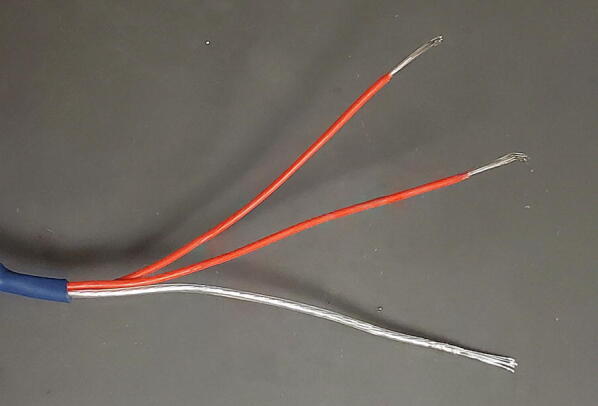
Stripped wires.

Next, cut the stud spade terminal connectors from the ends of the wires and strip about 5 mm of insulation from the ends of the wires (Fig. 93).

**Fig. 94:**
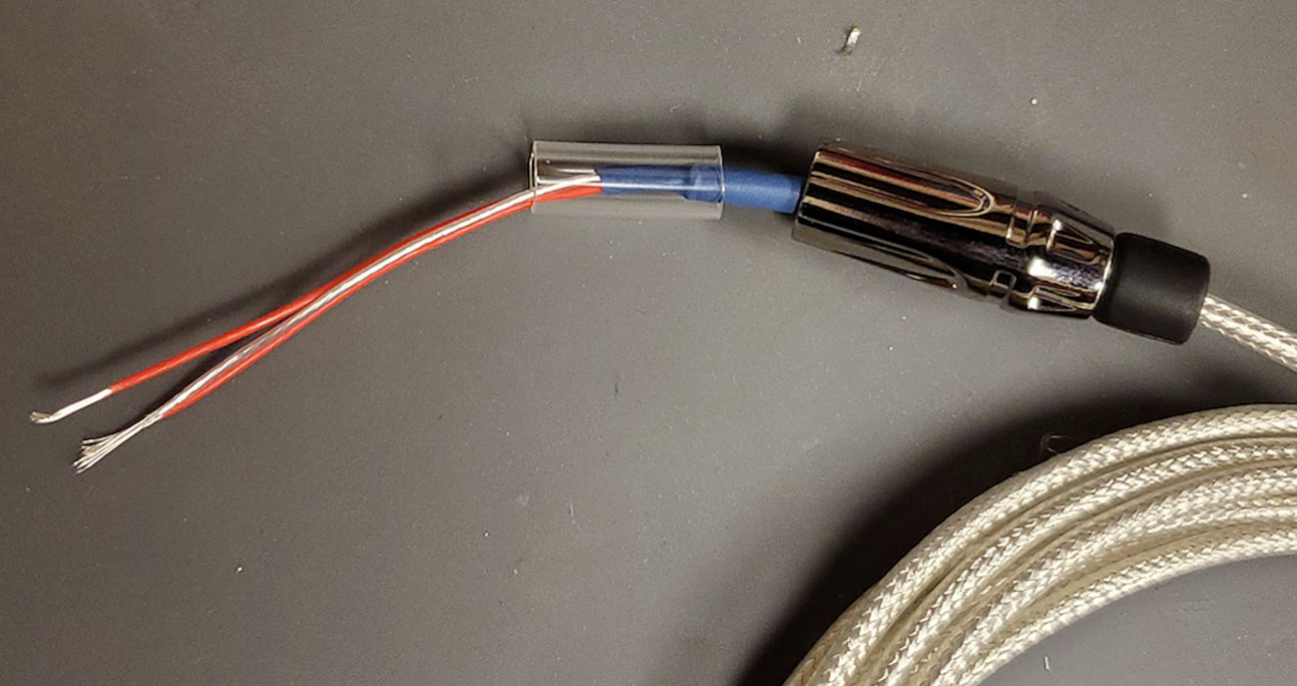
TRS jack housing.

Thread the 3.5 mm TRS jack housing and the plastic interior sleeve onto the temperature sensor wires from the side of the cut wires as shown (Fig. 94).

**Fig. 95:**
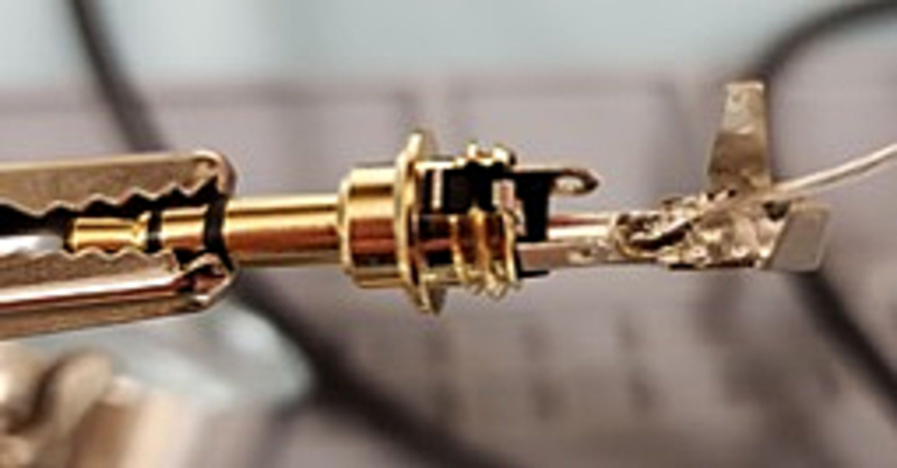
Center post.

Solder the wire that is not shorted to either other wire to the central post of the 3.5 mm TRS jack. It is generally easiest to first add some solder to the post first, and then reheat it to add the wire (Fig. 95).

**Fig. 96:**
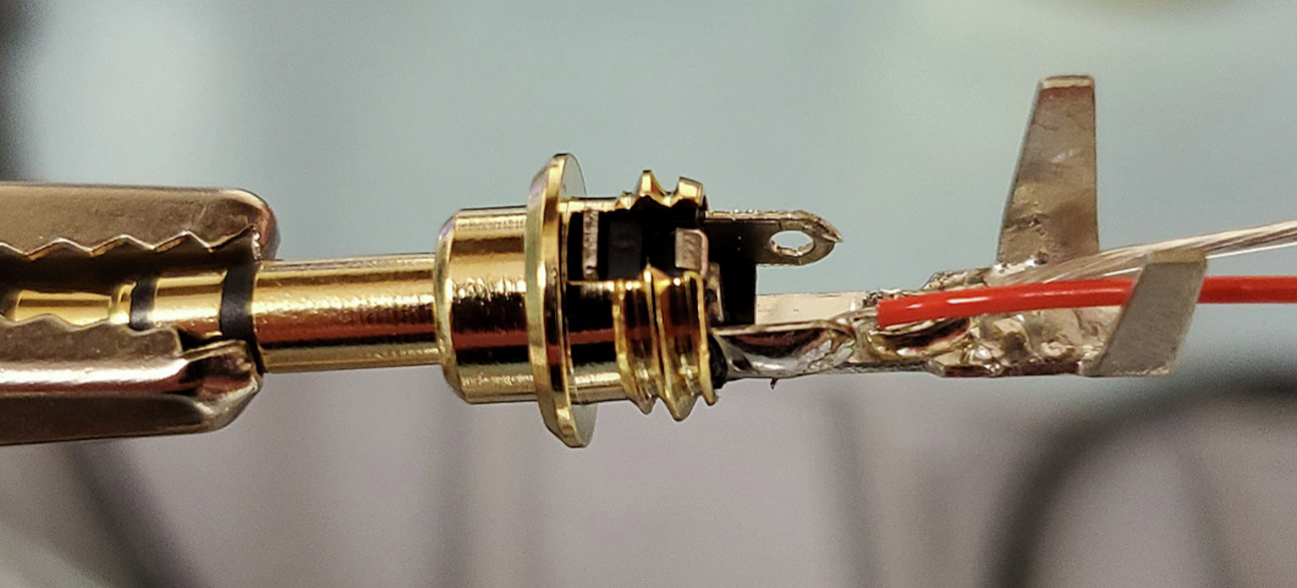
Lateral post.

Next, solder the two wires that are shorted together to the two lateral posts on the 3.5 mm TRS jack. It works best to thread the bare wire through the holes on these posts and solder on the outer side of the post. (Figure 96 shows one wire soldered in place; you will do both.)

**Fig. 97:**
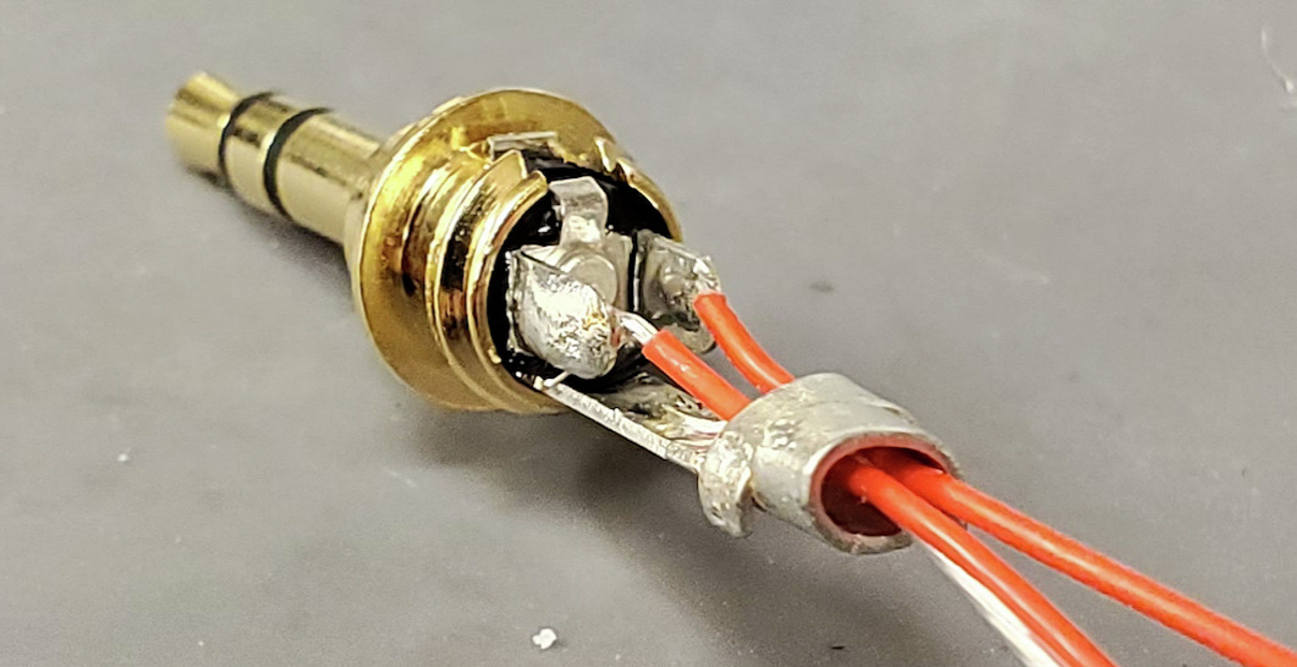
Tabs on central post.

Next, using pliers, bend the two upright tabs on the end of the central post over to cover the wires. Be careful not to crimp these tabs tightly onto the wires because it can cut the insulation and cause a short (Fig. 97).

**Fig. 98:**
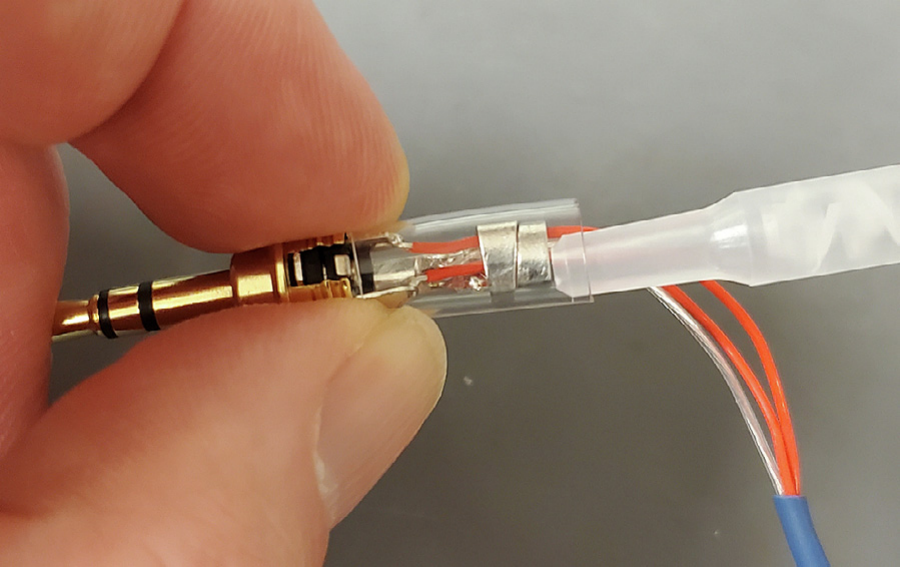
Epoxy in plastic sleeve.

Next, slide the plastic sleeve over the central and lateral posts and fill with epoxy (Fig. 98).

**Fig. 99:**
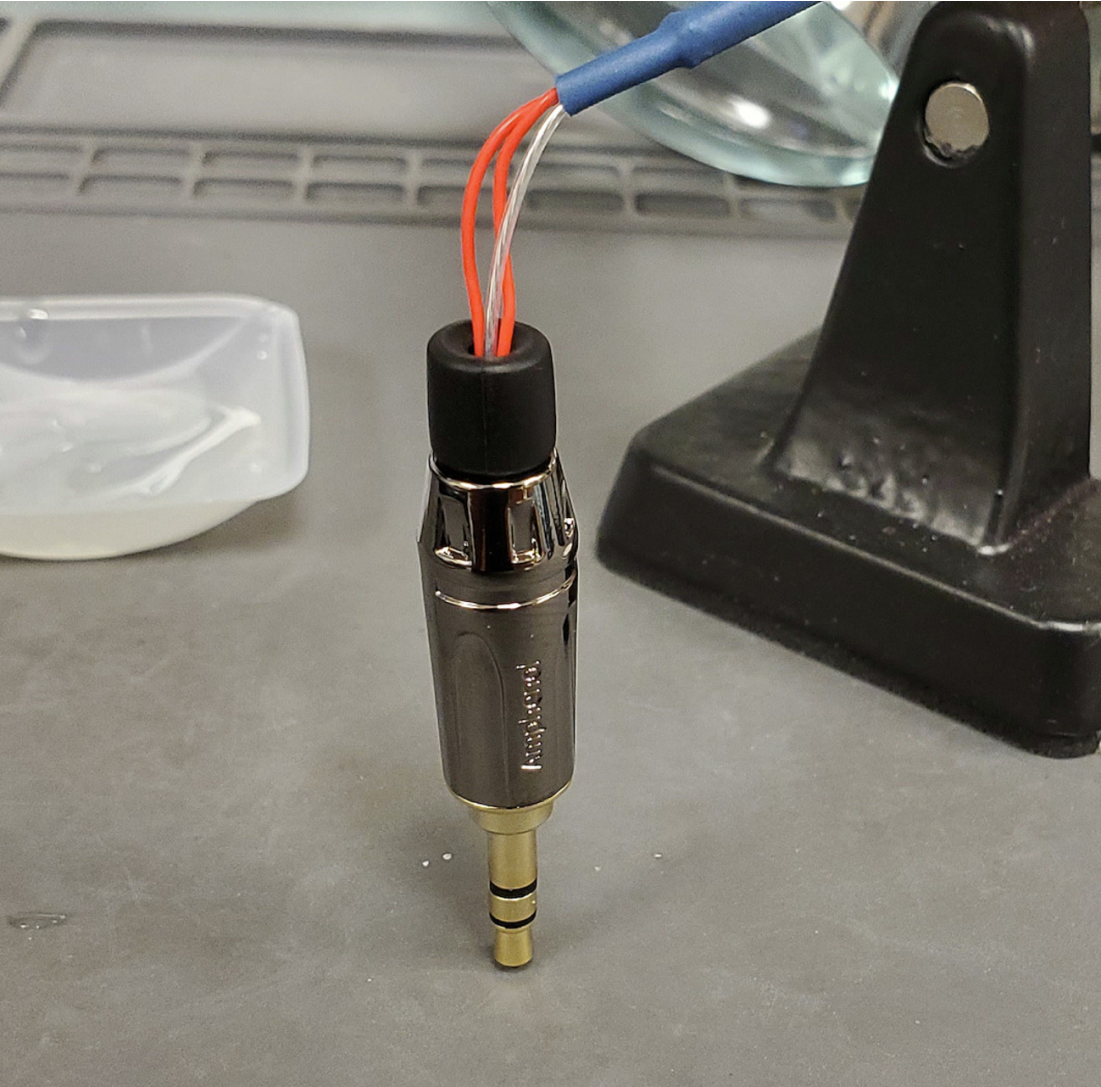
Finished assembly with epoxy curing.

Finally, slide the 3.5 mm TRS jack housing over the assembly and screw down onto the tip to secure in place. Place the jack upright with the tip pointing down to allow the epoxy to cure (Fig. 99).

### Google docs integration

• The Tank Controller devices can report current pH and Temperature readings for the tank to a Google Sheet. This will allow viewing of the current tank status from anywhere you have an internet connection. Google Docs integration, however, is optional and the basic functionality of the device is not affected if you do not complete these steps. To perform the following steps, you will need to be signed in with a Google account.
• Go to the following link to find the Tank Controller Monitor template: (https://docs.google.com/spreadsheets/d/1IrcRgtypfAYEB23MBk3Bh1gWOA9KFftv4Z9MHpWA5ns/edit?usp = sharing) Make a copy of this template for yourself by clicking on “File” in the menu, then “Make a copy”. This will bring up the “Copy document” dialog box. In the box labeled “Name”, the default name will be “Copy of Tank Controller Monitor Template”. Instead, give the document any name that you wish. Then click “OK”. Next find the spreadsheet ID of your copy of the Tank Controller Monitor spreadsheet and copy it. You can find it in the URL address of the sheet; it is the seemingly random string of letters and numbers following the “/spreadsheets/d/” portion of the URL and continues until there is another forward slash.
• Next you will set up the script to handle the incoming data. Navigate to the url: script.google.com. Click the “New project” button in the upper left of the page to start a new script. This will bring you to a code window. Into the code window paste this code found at the following URL: (https://docs.google.com/document/d/1oyupNPuR41Uw8On4gPO-X4F8IePKwD16_Qx1jdiEuqA/edit?usp = sharing). Then replace the section that says “\<enter spreadsheet ID here\>” with your spreadsheet ID. Next, in upper right click the “Deploy” button, then “new deployment”. In the window that pops up, click the cog next to “Select type”, then select “Web app”. Then under “Configuration”, type in anything that makes sense under “Description”. Under “Execute as” select “Me”. Then under “Who has access”, select “Anyone” (not “Anyone with Google account”). Then click “Deploy”. When you click “Deploy”, another window will appear that says, “The Web App requires you to authorize access to your data.”. Click “Authorize access” and select your Google account in the next window. Next a window will say “Google hasn't verified this app”. Click “Advanced” in the lower left and corner and “Go to Untitled project (unsafe)”. In the next window click “Allow”. Another window will show up that will say “Deployment successfully updated.”. Under that it will say “Web app”, and under that “URL.”
• Copy that URL by clicking the “Copy” button below it and save it somewhere you can find it again such as in a text document or a Word document. You will need this URL when setting up PushingBox in the next section of the instructions.
• To test if the script is working paste the URL you just saved with “?tankid = 1&tempData = 23&pHdata = 4.555″ added to the end of it into a new browser tab. Does ”ok“ show up in the browser? If so, check your Tank Controller Monitor spreadsheet. You should see the data show up under ”Raw_Data“ tab that report Tank 1 with a temperature of 23 and a pH of 4.555. The data should also appear for Tank 1 under the Dashboard tab.
• To setup the PushingBox function that will pass data from the Tank Controller to Google, navigate to < https://www.pushingbox.com/> and click “Login with Google” in the upper right-hand corner. Complete the login with your Google login credentials. Next, click on “My Services” in the menu towards the top of the page, then click the “Add a Service” button on the next page. Now click on “Select this service” next to “Custom URL”. It will be at or near the bottom of the options. Give this service a name in the top box labeled “Name of your CustomURL configuration:”. It doesn't really matter what the name is, as long as you can recognize it. Now, paste google script macro “Current web app URL” into “Root URL” (without the “?tankid = 1&tempData = 23&pHdata = 4.555″ you added for testing). Leave method as GET and click the ”Submit“ button.
• Now click on “My Scenarios”, then in the box under “Create a scenario or add a device” put in some name of your choosing (I use “Tank ID, Temp and pH Push”), and click “Add”. Now, click the “Add an Action'' button. Then, click ”Add an action to this service“ next to the service you made in the previous step. In the box under ”Data“ copy and paste in the text: ”?tankid=$tankid$&tempData=$tempData$&pHdata=$pHdata$“ (without the quotes). The click ”Submit“. Now, copy DeviceID that should be right under the scenario name. It should start with ”v“. Now, copy that DeviceID into Tank Controller Arduino code on line 7 where it says PushingBoxIdentifier. You must have the quotes around the DeviceID.
• Once you flash this code onto your Tank Controller Arduino, it should start to upload temperature and pH data to the Google sheet at the interval at which your Google Sheet interval is set for in the device.

### Software installation

• First, install the Arduino Desktop IDE (https://www.arduino.cc/en/software).
o Download and unzip the appropriate executable for your platform (Linux, macOS, or Windows).
o Copy the directory or package to an appropriate place for applications on your platform.
o Follow the Getting Started Guide (https://www.arduino.cc/en/Guide/ArduinoMega2560) to confirm that you can install and run the “Blink” software.
• From the Arduino IDE select the menu command ‘Tools -> Manage Libraries‘. Search for ‘TankController‘, click the ‘Install‘ button, and close the Library Manager.
• From the Arduino IDE select the menu command ‘File -> Examples -> TankController -> TankController‘.
o If you have a PushingBox Identifier (from section 5.13 above), add it where indicated on line 7.
• Click the “Upload” button (a right-facing arrow) or select the “Sketch / Upload” menu.
• If you do not have an Ethernet cable attached, you can shorten the startup time by pressing any key to skip the DHCP request; release the key when you see the idle screen (with current values and targets for pH and temperature).

## Operation instructions

### Device setup

• Connect standard 3-prong power cable (P48) to the device and plug into a standard power outlet.
• Connect an ethernet cable to the device. (This is optional. You can press and hold any button during the device startup to forgo the network connection attempts and avoid the timeout waiting for a response).
• The Open Acidification TankController works with most pH glass electrodes with a BNC connector but will not work with other sensor types such as ISFET or optical pH sensors. Attach a compatible pH electrode to the BMC port and place the sensor into the aquarium or header tank to be controlled.
• Attach the previously prepared PT100 sensor to the 3.5 mm jack on the TankController and place the end of the temperature sensor into the aquarium or header tank to be controlled.
• Plug a temperature control device into the left electrical outlet on the bottom of the TankController. This could be an aquarium chiller or an aquarium heater.
• Plug a CO2 addition device into the right electrical outlet. This CO2 addition device is typically a solenoid-activated CO2 regulator but could also be a flow valve between a header tank and the aquarium.

### Software usage

#### Software menus

Because of the limitations of our input and output devices (a 4x4 keypad and a 16x2 LCD), the user interface is designed to be relatively simple. The initial (or idle) screen shows the current values and targets for pH and temperature. The general format of the idle screen is as follows:

On the first line we have information on the tank pH. First is the current value (here 7.825). Next is a ‘B’ to indicate that the device has turned on the CO_2_ bubbler (or a blank if not). The last value on the first line is the target pH (here 8.100). On the second line we have information about the tank temperature. First is the current value (here 19.25 degrees Celsius). Next is a character to indicate the state of the temperature adjustment. It is one of four values:

• ‘h’ to indicate that the device is configured to heat but the heater is off.
• ‘H’ to indicate that the heater is on.
• ‘c’ to indicate that the device is configured to cool but the chiller is off.
• ‘C’ to indicate that the chiller is on.

The final value on the second line is the target temperature in degrees Celsius. From this screen you can select a menu to view settings or a menu to change settings.

#### Top-level menu

The idle screen is the first of three top-level screens:

• Current status (Idle)
• View Settings
• Change Settings

The device uses the keypad for menu selection and data entry. For menu navigation, consider the 10-digit keypad on the left with bolded keys having meanings of up, left, right, and down as follows:



In this configuration the ‘2′ key rotates up through the current menu options, the ‘8′ key rotates down through the current menu options, the ‘4′ key returns to the previous menu level, and the ‘6′ key selects (enters) the menu currently displayed. So, pressing '8′ (down) from the idle screen will take you to the View Settings screen:



Note the “^2″ for ”up“ and the ”v8″ for “down”. This is a reminder of how to go up and down through the top-level menu screens. From here, pressing '2′ will go “up” to the idle screen and pressing '8′ will take you “down” to the Change Settings screen:



From here pressing '2′ will go “up” to the View Settings screen and pressing '8′ will take you “down” and loop back to the top to show the idle screen.

#### View Settings menu

When on a menu option you can select it by pressing '6′ (right). In the case of the “View settings” menu, you will be taken to a new sequence of menus (the exact menu options and their order will depend on the version of the software installed):

• View time
• View PID
• View pH slope
• View tank ID
• View log file
• View Google mins
• View IP and MAC
• View version
• View free memory

As with the top-level menu, you can move down (8) and up (2) and you can select an item (6). In addition, you can return to the top-level menu by pressing '4′ (left). Selecting a menu item (6 > ) will take you to the state where you are viewing the requested information. In most cases you can exit from this state by pressing any key. If you don't press a key, then after a minute the device returns to the idle screen.

#### Change Settings menu

You can select the Change Settings menu by rotating through the top-level menus (‘2′ or ‘8′) till “Change settings” is visible and then pressing '6' (right) to be taken to a new set of menu options. As before, you can move up (2), down (8), left (4), and right (6).

• Set pH target
• Set temperature
• pH calibration
• Clear pH calibration
• Temp calibration
• Set KP
• Set KI
• Set KD
• PID on/off
• Set chill/heat
• Set Google mins
• Set date/time
• Set Tank ID

### Common software tasks and shortcuts.

• In data entry screens, several keys provide functions to the users in addition to number entry. The “*” (star) button can be used as a decimal place, “A” is used for “accept the entered value”, “B” is used for “backspace”, “C” is used for “clear the current input” and “D” aborts the current data entry and returns the user to the idle screen.
• Setpoints for pH and temperature can be directly accessed from the idle screen by pressing “A” or “B” as shortcuts, respectively.
• When setting pH or temperature setpoints, after entering the setpoint value and accepting the user will be asked for a ramp time in hours. Over the specified time the device will perform a linear setpoint ramp from the current measured value to the new setpoint. The default value is 0, which if accepted will immediately set the new setpoint to the value entered. (Of course, the change in the tank will not be instantaneous!)
• When controlling temperature, you must set if the temperature control device will heat or chill the water being controlled for the device to work properly and not result in a positive feedback loop and runaway temperature. You can set this parameter in the “Change Settings” menu under “Set chill/heat”.
• The TankController pH meter can be calibrated using a 1-point, 2-point or 3-point calibration routine. Calibration of pH can be accomplished by selecting “pH calibration” in the “Change Settings” menu and following the on-screen prompts. To use a 1-point calibration, simply press “D” after completing calibration to the first buffer, to use a 2-point calibration press “D” after calibration to the second buffer and to using a 3-point calibration the process will automatically return to you the idle screen after calibrating to the third buffer. When performing a 2-point calibration you must calibrate to the higher pH buffer first, then the lower pH buffer. When performing a 3-point calibration you must calibrate the midpoint pH buffer first, then the lowest pH buffer, and finally the highest pH buffer.
• While the default pH control PID settings have worked moderately well for most seawater control systems we have tested it on, tuning the PID for the specific systems is essential for optimal pH control. PID constant can be adjusted using the “set KP”, “set KI” and “set KD” options in the “Change Settings” menu. A full description of tuning a PID is beyond the scope of this paper. However, very good pH control can be achieved by only adjusting the KP value. In short, if you find that pH unacceptably overshoots the setpoint, you can reduce the KP value. If you find that pH approaches the setpoint too slowly, you can increase the KP value. While a PID autotune algorithm has not been integrated into the system at this time, it is something that is planned in future software versions.

## Validation and characterization

The Open Acidification Tank Controller has already been used in two studies that have resulted in peer-reviewed publications: one investigating the impact of ocean acidification on immune system function in *Octopus rubescens* [18], and the other investigating the effect of ocean acidification on the routine metabolic rate and oxygen supply capacity in *Muusoctopus leioderma* [19].

Performance of pH setpoint holding, ramping, and sine wave functionality of the Open Acidification Tank Controller was tested in a 935 L saltwater holding tank. A solenoid-controlled CO_2_ regulator was plugged into the Tank Controller and attached to a 5-pound CO_2_ gas cylinder. While any glass pH probe using a BNC connector can be used the Tank Controller, for this test we used an relatively inexpensive probe, the Atlas Scientific consumer grade pH probe (https://atlas-scientific.com/probes/consumer-grade-ph-probe/), costing less than $50. This is the same probe that was used in the previously published work using this device [18], [19]. This probe has a published response time of 95% in 4 s. With this pH probe, we calibrated using a one-point calibration daily. In our experience, these probes can typically last for 12 months of continuous use before needing to be replaced.

**Fig. 100:**
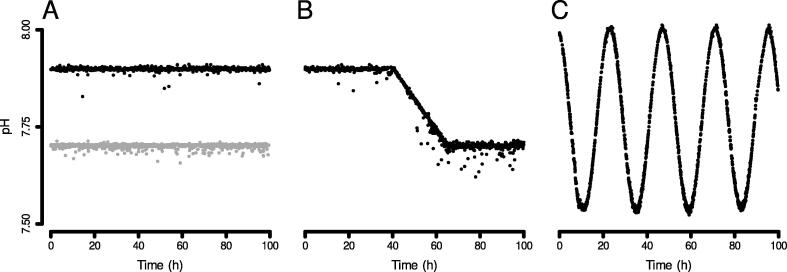
Plots of steady-state pH hold (A) with a setpoint of 7.9 (black points) or 7.7 (gray points), a 24-hour ramp between 7.9 and 7.7 (B), and sine wave with a mean value of 7.75, amplitude of 0.25, and period of 24 h (C).

This device has been found to work well with the CO_2_ regulators by AQUATEK, AZT Plus, MOD Complete, and FZONE; but the CO_2_ regulator made by MANATEE causes resetting issues with the Tank Controller. The regulator was connected to an air stone bubbler in the holding tank by a standard aquarium air line. All pH measurements are the reported values from the Tank Controller. Saltwater had a measured salinity of approximately 35 psu and was held constant at a temperature of 23C by the Tank Controller. When the pH setpoint was set to 7.9, the tank had a measured pH of 7.899 ± 0.005 (mean ± SD) over the course of 100 hr (Fig. 100A, black points). When set to a pH setpoint of 7.7, the tank had a measured pH of 7.701 ± 0.005 (Fig. 100A, gray points). The Tank Controller also performed a 24-hour pH ramp from 7.9 to 7.7 (Fig. 100B), and a pH sine wave with a mean value of 7.75, amplitude of 0.25, and period of 24 h (Fig. 100C). This final function (sine wave pH control) is completely unique to ocean acidification pH tank controllers to our knowledge.

The level of control achieved by the Open Acidification Tank Controller, both in this study and other published studies using the device, is comparable to that demonstrated by commercially available devices (Table 1).

**Table 1:** Survey of standard deviations reported for ocean acidification experiments reported using various pH-stat devices. System-measured pH SD is the standard deviations of pH measurements taken of the pH-controlled aquarium by the control device. Independent pH SD is the standard deviation of pH measurements taken of the pH-controlled aquarium by a method other than using the control device’s pH measurements.

Device	Commercial/Open Source	System-measured pH SD	Independent pH SD	Citation
Open Acidification Tank Controller	Open Source	0.005		This study
Open Acidification Tank Controller	Open Source		0.02–0.04	[18]
Open Acidification Tank Controller	Open Source		0.05–0.06*	[19]
American Marine Pinpoint	Commercial	0.05		[20]
American Marine Pinpoint	Commercial	0.04–0.16		[21]
American Marine Pinpoint	Commercial	0.04–0.06		[13]
Neptune Systems Apex	Commercial	0.07*		[22]
Neptune Systems Apex	Commercial		0.05*	[23]
Digital Aquatics Reefkeeper Elite (modified)	Commercial	0.01		[11]
Omega Engineering PHCN-901	Commercial	0.01		[11]
Loligo Systems	Commercial	0.02–0.03		[24]
Loligo Systems	Commercial	0.03–0.05		[25]
Loligo Systems	Commercial		0.01–0.09	[26]
GHL ProfiLux	Commercial		0.2	[27]
GHL ProfiLux	Commercial		0.02–0.04	[28]
GHL ProfiLux	Commercial	0.01		[29]
Arduino Aquarium pH Control System (AAPCS)	Open Source	0.05–0.07^a^		[15]
Unnamed	Open Source	0.006		[16]

* Standard error reported in the publication, and here converted to standard deviation.

a Range reported instead of standard deviation.

## Ethics statements

The design and construction of the hardware and software of the Open Acidification Tank Controller did not involve any human subjects or animal experiments.

## CRediT authorship contribution statement

**Kirt L Onthank:** Conceptualization, Methodology, Software, Validation, Formal analysis, Investigation, Resources, Data curation, Writing – original draft, Writing – review & editing, Visualization, Supervision, Project administration, Funding acquisition. **James Foster:** Software, Resources, Writing – review & editing, Supervision. **E. Preston Carman Jr:** Software. **John E. Foster:** Software. **Monica Culler-Juarez:** Visualization, Investigation, Validation. **Eliam Calvo:** Software. **Wesley Duerksen:** Software. **Trevor Natiuk:** Software. **Lucas Saca:** Software.

## Declaration of Competing Interest

The authors declare that they have no known competing financial interests or personal relationships that could have appeared to influence the work reported in this paper.
